# Synthesis and Biological Evaluation of Novel Aminochalcones as Potential Anticancer and Antimicrobial Agents

**DOI:** 10.3390/molecules24224129

**Published:** 2019-11-15

**Authors:** Joanna Kozłowska, Bartłomiej Potaniec, Dagmara Baczyńska, Barbara Żarowska, Mirosław Anioł

**Affiliations:** 1 Department of Chemistry, Wrocław University of Environmental and Life Sciences, Norwida 25, 50-375 Wrocław, Poland; b.potaniec@gmail.com (B.P.); miroslaw.aniol@upwr.edu.pl (M.A.); 2 ŁUKASIEWICZ Research Network - PORT Polish Center for Technology Development, Stabłowicka 147, 54-066 Wrocław, Poland; 3 Department of Molecular and Cellular Biology, Faculty of Pharmacy with Division of Laboratory Diagnostics, Wroclaw Medical University, Borowska 211A, 50-556 Wrocław, Poland; dagmara.baczynska@umed.wroc.pl; 4 Department of Biotechnology and Food Microbiology, Wrocław University of Environmental and Life Sciences, Chełmońskiego 37, 51-630 Wrocław, Poland; barbara.zarowska@upwr.edu.pl

**Keywords:** aminochalcone, anticancer activity, antimicrobial activity

## Abstract

A series of 18 aminochalcone derivatives were obtained in yields of 21.5–88.6% by applying the classical Claisen-Schmidt reaction. Compounds **4**–**9**, **14** and **16**–**18** with 4-ethyl, 4-carboxy-, 4-benzyloxy- and 4-benzyloxy-3-methoxy groups were novel, not previously described in the scientific literature. To determine the biological properties of the synthesized compounds, anticancer and antimicrobial activity assays were performed. Antiproliferative potential was evaluated on four different human colon cancer cell lines—HT-29, LS180, LoVo and LoVo/DX —using the SRB assay and compared with green monkey kidney fibroblasts COS7. Anticancer activity was described as the IC_50_ value. The best results were observed for 2′-aminochalcone (**1**), 3′-aminochalcone (**2**) and 4′-aminochalcone (**3**) (IC_50_ = 1.43–1.98 µg·mL^−1^) against the HT-29 cell line and for amino-nitrochalcones **10**–**12** (IC_50_ = 2.77–3.42 µg·mL^−1^) against the LoVo and LoVo/DX cell lines. Moreover, the antimicrobial activity of all derivatives was evaluated on two strains of bacteria: *Escherichia coli* ATCC10536 and *Staphylococcus aureus* DSM799, the yeast strain *Candida albicans* DSM1386 and three strains of fungi: *Alternaria alternata* CBS1526, *Fusarium linii* KB-F1 and *Aspergillus niger* DSM1957. In the case of *E.* *coli* ATCC10536 almost all derivatives hindered the bacterial growth (∆OD = 0). Furthermore, the best results were observed in the presence of 4′-aminochalcone (**3**), that completely limited the growth of all tested strains at the concentration range of 0.25–0.5 mg·mL^−1^. The strongest bacteriostatic activity was exhibited by novel 3′-amino-4-benzyloxychalcone (**14**), that prevented the growth of *E. coli* ATCC10536 with MIC = 0.0625 mg·mL^−1^.

## 1. Introduction

Chalcones (1,3-diphenyl-2-propene-1-ones) belong to a large group of bioactive flavonoids. They are known as secondary metabolites of plants, which protect them from damage caused by microorganisms, insects and animals. The chemical structure of these compounds is based on the presence of two aromatic rings—A and B—connected with the three carbon chain containing an α,β-unsaturated bond and a carbonyl group [1]. Chalcones maintain an unflagging interest among scientists due to their broad spectrum of biological activities, including antibacterial [2], antifungal [3], anticancer [4,5,6], antioxidant [7] and anti-inflammatory properties [8]. Their biocapability is correlated with the different electron-donor and electron-acceptor groups attached to the aromatic rings [9].

The literature describes chalcones containing a single amino group as promising agents with antitumor [10], antimicrobial [11,12] and antioxidant activity [13]. The DPPH assay performed on 2′-amino- and 4′-aminochalcones proved their ability to scavenge free radicals. The mentioned activity was satisfactory and comparable to that of ascorbic acid, a reference compound known for its high antioxidant properties [14,15]. Additionally, the presence of less than five hydrogen donors and no more than 10 hydrogen bond acceptors in the chalcones’ chemical structure, molecular weight lower than 500 Da and the octanol-water partition coefficient (logP) not greater than 5, is consistent with Lipinski’s rule of five and indicates the possible applicability of aminochalcones as drug candidates [16]. Molecular modeling techniques confirm the potential of monosubstituted chalcones and their usefulness as anti-inflammatory agents. Interesting results were obtained for 2′-aminochalcones, which docking score was on a similar level to that of the widely used drug ibuprofen [17]. Moreover, 4′-aminochalcone (**3**), 4′-amino-4-fluorochalcone and 4′-amino-4-methyl- chalcone exhibited strong inhibitory activity against myeloperoxidase (MPO)—an enzyme, involved in numerous inflammatory diseases. The IC_50_ values of these aminochalcones against MPO were comparable to that of 5-fluorotryptamine—a potent inhibitor of this enzyme [18]. Furthermore, the position of the amino group attached to the aromatic ring has a significant influence on the biological properties of the compounds. Trein et al. tested the activity of 2′-aminochalcone (**1**), 3′-aminochalcone (**2**) and 4′-aminochalcone (**3**) against the parasitic protozoan *Trichomonas vaginalis*, which contributes to serious reproduction disorders, cervical cancer and a predisposition to prostate cancer. The scientists observed the best results for compound **2**, compared to a popular antibiotic—metronidazole [19].

Besides, 2′-aminochalcones substituted at the C-4′, C-5′, C-2, C-3 and C-4 positions have demonstrated cytotoxic activity against epidermoid carcinoma of the nasopharynx (KB), breast cancer (MCF-7), lung carcinoma (A-549) and ovarian cancer (1A9) cell lines [20]. Also, 2′-aminochalcone (**1**) was described as the most potent inductor of apoptosis in TRAIL-resistant human colon cancer (HT-29) cells [4]. Santos et al. reported that chalcones with hydrophobic substituents on ring B such as 4′-amino-4-fluorochalcone, 4′-amino-4-chlorochalcone and 2′-amino-4-methylchalcone were the strongest inhibitors of canine malignant histiocytic cell line (DH82) proliferation with IC_50_ values of 34.4, 31.4 and 38.2 mM, respectively. The authors proved that the mechanism of action is based on apoptosis rather than necrosis compared to doxorubicin [21]. Moreover, 4′-amino-4-fluorochalcone and 4′-amino-2-chlorochalcone were also described as promising agents against human gastric cancer cell line (SGC7901) with MIC values of 7.44 µM and 11.15 µM, respectively. It is noteworthy that their cytotoxic effects were higher than that of the reference—5-fluorouracil (MIC = 29.19 µM) [12]. The anticancer activity of 4′-amino-1-naphthyl- chalcone and 4′-amino-4-methyl-1-naphthylchalcone described by Seba et al. proved the ability of these derivatives to inhibit the migration and invasion of osteosarcoma cells, especially in p53-expressing cells (U2OS) [22]. Moreover, Santos et al. showed that aminochalcones containing a fluorine atom at the C-2 position and a pyridyl group at the C-3 position induced apoptosis due to upregulated p53 protein expression in the breast cancer cell lines MCF-7 (ER) and MDA-MB-231 (TNBC) [23]. The studies of Pati et al. proved that incorporation of an amino moiety into the ring B of the methoxychalcones may increase their cytotoxicity against a murine melanoma cell line (B16) with the effect depending on the location of the substituent [10].

The search for novel antibacterial and antifungal candidates, especially of natural origin, maintains an unabated interest in the scientific community. Lin et al. described the high potential of 3′-amino-2-iodochalcone to inhibit the growth of *Mycobacterium tuberculosis*, a Gram-positive pathogenic bacterium which is an tuberculosis agent [24]. Moreover, 4′-aminochalcones, especially those with methoxy substituents attached to the ring B, exhibited antimicrobial activity against *Escherichia coli*, *Staphylococcus aureus*, *Candida albicans* and *Trichophyton mentagrophytes* [11,25]. Furthermore, the presence of a -NO_2_ group attached to the C-4 position enhances by 2–3 times the antifungal activity against *Microsporum canis*, *Trichophyton rubrum* and *Epidermophyton floccosum* in comparison to unsubstituted chalcone [26]. Additionally, 4′-aminochalcones with fluorine, chlorine or bromine atoms at the C-2, C-3 and C-4 positions in the B ring inhibited the growth of *Bacillus pumilis*, *Bacillus subtilis* and *Proteus vulgaris*. Likewise, strong fungistatic activity of these derivatives was observed against *Aspergillus niger*, *C. albicans* and *Rhizopus oryzae*. Both antibacterial and antifungal properties of aminochalcones were comparable with the appropriate standards—amikacin and fluconazole [27].

In this paper, we describe the chemical synthesis of a library of 18 aminochalcones, including 10 novel derivatives, which have not been previously described in scientific reports. The next step was the evaluation of the biological properties of all synthesized compounds. Cytotoxicity tests were performed on four types of human colon cancer cell line—HT-29, LS180, LoVo and LoVo/DX and normal green monkey fibroblast line COS7. Antitumor activity was expressed as the IC_50_ value and compared with standard compounds—cisplatin and doxorubicin. Additionally, the antimicrobial activity was determined against two strains of bacteria *Escherichia coli* ATCC10536 and *Staphylococcus aureus* DSM799, the strain of yeast *Candida albicans* DSM1386 and three strains of fungi—*Alternaria alternata* CBS1526, *Fusarium linii* KB-F1 and *Aspergillus niger* DSM1957 and expressed as microbial growth curves. For compounds which completely hindered the growth of microorganisms (∆OD = 0), the minimal inhibitory concentrations (MIC) values were evaluated.

## 2. Results and Discussion

**Scheme 1:**
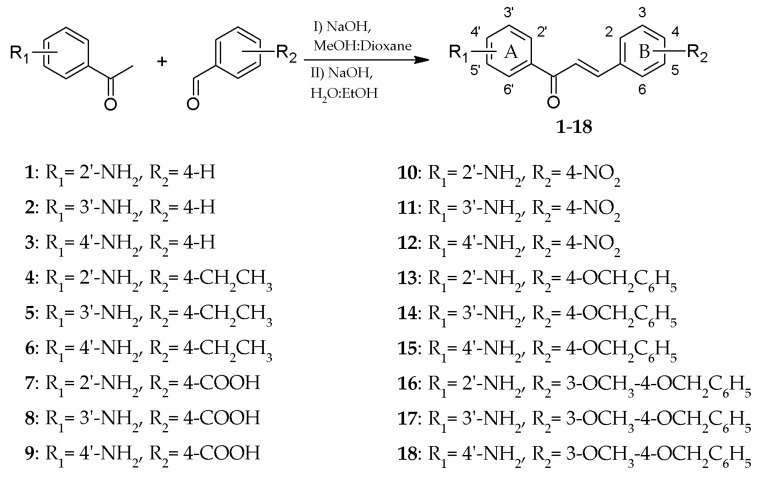
Synthesis of aminochalcones **1**–**18**.

Aminochalcones were obtained as the result of base-catalyzed condensation reactions of 2′-aminoacetophenone, 3′-aminoacetophenone and 4′-aminoacetophenone with appropriate benzaldehydes containing 4-ethyl, 4-nitro, 4-carboxy, 4-benzyloxy and 4-benzyloxy-3-methoxy groups. Compounds **1**–**6** and **10**–**18** were obtained according to modified protocol described by Amir et al. [28]. Reactions were performed on a magnetic stirrer using a mixture of methanol and 1,4-dioxane 1:1 (*v*:*v*) as solvent in the presence of sodium hydroxide (Scheme 1). After complete conversion of the substrates, the reaction mixtures were poured into ice water and the precipitated crystals were collected and purified using liquid column chromatography.

In the case of amino-carboxy derivatives **7**–**9**, the reactions were performed in mixture of ethanol and water 2.5:1 (*v*/*v*) in the ice-bath at the time of addition of 4-formylbenzoic acid. After the completion of the reaction, 1 M hydrochloric acid was added until the product crystals precipitated [29]. The reaction progress and the purity of synthesized compounds were analyzed using thin layer chromatography (TLC) and high performance liquid chromatography (HPLC). As a result of our research, the 18 aminochalcones were obtained with the yields of 21.5–88.6%. Compounds **4**–**9**, **14** and **16**–**18** have never been synthesized earlier, according to scientific reports.

The structures of all compounds were determined by ^1^H and ^13^C nuclear magnetic resonance (NMR), high resolution mass spectrometry (HRMS) and Fourier-transform infrared spectroscopy (FTIR). Analysis of the ^1^H-NMR spectra confirmed the presence of the α,β-unsaturated bonds in each derivative. Characteristic signals from H-α and H-β appeared as doublets with coupling constants *J* = 15.5–15.7 Hz. Furthermore, a peak in the 185.39–191.69 ppm range in the ^13^C-NMR spectra indicated the presence of the carbonyl group. Moreover, two signals in the 120.83–127.58 ppm and 138.63–144.50 ppm regions were assigned to C-α and C-β, respectively.

Lipiński’s rule of five (known also as Pfizer’s rule of five) allows one to predict if a compound has chemical and physical properties that would likely make it an orally active drug. In relation to this principle, we calculated the molecular weight (MW), partition coefficient (logP), number of hydrogen bond acceptors (nON) and number of hydrogen bond donors (nOHNH) of all synthesized derivatives (Table 1). According to the rule of five, all aminochalcones **1**–**18** were characterized by parameters consistent with Lipiński’s rule of five, which suggest that they could be taken into consideration as potential medicines.

The next step of our research was the evaluation of the biological properties of all synthesized aminochalcones. It is worth mentioning that there is no correlation between anticancer and antimicrobial activities. However, as a result of our work, we present the biological potential of the synthesized aminochalcones **1**–**18** concerning their antiproliferative and also antimicrobial activity.

Anticancer activity was evaluated on four different human colon cancer cell lines: HT-29, LS180, LoVo and LoVo/DX. Due to problems with the mortality of cells isolated from healthy tissues, which were characterized by a low proliferative potential and poor survivability, green monkey fibroblasts COS7 were selected as a normal cell line to determine the toxicity of aminochalcones. The cytotoxic effect of the compounds was investigated using the SRB assay and indicated as IC_50_ values in comparison with two reference drugs—cisplatin (**19**) and doxorubicin (**20**) [30]. Santos et al.’s research on the molecular mechanism of death of a canine malignant histiocytic cell line (DH82) proved that the action of 4′-amino-4-fluorochalcone, 4′-amino-4-chlorochalcone and 2′-amino-4- methylchalcone is based on apoptosis [21]. Furthermore, Mai et al. described 2′-aminochalcone (**1**) as the most potent and selective agent against TRAIL-resistant breast (MCF-7), ovarian (Caov-3), lung (A549), liver (HepG2), colorectal (HT-29), nasopharyngeal (CNE-1), erythromyeloblastoid (K-562) and T-lymphoblastoid (CEM-SS) cancer cells. The IC_50_ for compound **1** when tested on the HT-29 cell line was calculated as 4.39 µM (0.98 µg·mL^−1^). The authors also determined the molecular mechanism of action of 2′-aminochalcone (**1**) against the HT-29 cell line, which, like in the case of the DH82 cell line, is based on apoptosis [4]. In our investigation, 2′-aminochalcone (**1**) was the strongest inhibitor of HT-29 cell line proliferation (IC_50_ = 1.43 µg·mL^−1^) (Table 2), exhibiting almost 12 times higher activity than the reference compound cisplatin (**19**, IC_50_ = 16.73 µg·mL^−1^) and four times lower in comparison to doxorubicin (**20**, IC_50_ = 0.33 µg·mL^−1^). Furthermore, it was cytotoxic against the COS7 cell line with an IC_50_ value of 2.64 µg·mL^−1^, which indicated its poor selectivity against cancer cells, similar to that of cisplatin.

Another scientific reports described compound **3** as the most active against the human breast cancer cell line T47D with an IC_50_ value of 5.28 µg·mL^−1^ [31]. Furthermore, Wu et al.’s studies proved that 4′-amino-2-chlorochalcone demonstrated the strongest anticancer activity on the HT-29 cell line with an IC_50_ value of 26.25 µM [12]. In our research, 4′-aminochalcone (**3**) exhibited the highest anticancer activity on the HT-29 cell line (IC_50_ = 1.98 µg·mL^−1^) among the 4′-aminochalcones **3**, **6**, **9**, **12**, **15**, **18**. Similar activity was observed for 3′-aminochalcone (**2**) against all tested human colon cancer cell lines (IC_50_ values in range of 1.60–2.13 µg·mL^−1^). Moreover, compounds with ethyl, nitro and benzyloxy groups attached at the C-4 position and 4-benzyloxy-3-methoxy moieties on ring B also represented a group of inhibitors stronger than cisplatin against the HT-29 cell line. In the case of amino-carboxy derivatives **7**–**9**, the location of the amino group had a significant impact on the IC_50_ value. The activity decreased in the following order: 2′-amino-4-carboxychalcone (**7**) > 3′-amino-4-carboxychalcone (**8**) > 4′-amino-4-carboxychalcone (**9**). A similar trend was observed on other tested cell lines.

In the case of 2′-amino-4-nitrochalcone (**10**), the scientific literature reports it as a weaker antitumor agent in comparison to 2′-aminochalcone (**1**) against HT-29 [4]. In our research a similar effect was observed. Incorporation of the electron-withdrawing -NO_2_ group at the C-4 position resulted in about two times less cytotoxic effect on the HT-29 cell line. However, in the case of the LS180, LoVo and LoVo/DX cell lines, the activities of all investigated amino-nitrochalcones were similar to that of compounds **1**–**3** and comparable to cisplatin (**19**). Unfortunately, they were also toxic against monkey fibroblasts COS7 with IC_50_ values in range of 1.79–3.10 µg·mL^−1^. Only for 2′-amino-4-benzyloxychalcone (**13**) was it not possible to determine the antitumor properties in the tested concentration range for all tested cell lines.

Antimicrobial activity assays were performed in two stages. First, screening procedure was carried out on two strains of bacteria—*E. coli* ATCC10536 and *S. aureus* DSM799, strain of yeast *C. albicans* DSM1386 and three strains of fungi—*A. alternata* CBS1526, *F. linii* KB-F1 and *A. niger* DSM1957. Chosen method allowed to present the microbial growth curves and calculate the duration of the lag-phase and increase of optical density (∆OD) in the presence of 18 aminochalcones at the concentration of 0.1% (*w*/*v*) (Table 3). In all cases of ∆OD = 0, minimal inhibitory concentration (MIC) values were evaluated (Table 4) and compared with reference substances: oxytetracycline (against bacteria), cycloheximide (against yeast) and nystatin (against fungi).

Prasad et al. described the antimicrobial potential of 4′-aminochalcones with halogen atoms and methoxy groups attached at various positions of ring B. These compounds effectively inhibited the growth of *B. subtilis*, *B. pumilus*, *S. aureus*, *E. coli* and *P. vulgaris* [27]. In our investigations, almost all aminochalcones (compounds **2**–**5**, **7**–**18**) prevented the growth of *E. coli* ATCC10536 at the tested concentration. Suwito et al. described 4′-aminochalcone (**3**) as the strongest inhibitor of *E. coli* ATCC25923 growth, comparable with two reference standards—sulfamerazine and sulfadiazine. Furthermore, this derivative was also active against *S. aureus* ATCC25922 and *C. albicans* ATCC1023 and described by the authors as the best wide spectrum antimicrobial agent candidate [31]. In our research, compound **3** was two times stronger against Gram-positive bacteria *S. aureus* DSM799 than Gram-negative *E. coli* ATCC10536, confirming the known phenomenon of higher susceptibility of Gram-negative strains to flavonoids [32]. Moreover, the activity of 4′-aminochalcone (**3**) against all tested microorganisms was expressed with MIC values of 0.25–0.5 mg·mL^−1^ (Table 4).

In the case of *S. aureus* DSM799 complete growth inhibition was observed in the presence of 2′-, 3′-, 4′-aminochalcones **1**–**3**, and aminochalcones with carboxy (**7**–**8**), nitro (**11**–**12**), benzyloxy (**13**) and 4-benzyloxy-3-methoxy (**16**) moieties (∆OD = 0). Among them, the new compounds 2′-amino-4-carboxychalcone (**7**) and 3′-amino-4-carboxychalcone (**8**) exhibited just as strong activity as the reference substance oxytetracycline, with a MIC value of 0.125 mg·mL^−1^. Moreover, six aminochalcones (**2**, **3**, **7**, **10**, **11** and **13**) prevented the growth of *C. albicans* DSM1386. Additionally, compound **7** showed four times higher activity in comparison to cycloheximide and was the best inhibitor of growth of this strain with a MIC value of 0.125 mg·mL^−1^. Only 3′-amino-4- benzyloxychalcone (**14**) did not affect to the growth of *C. albicans* DSM1386, but in contrast limited the growth of *S. aureus* DSM799, *A. alternata* CBS1526, *F. linii* KB-F1 and *A. niger* DSM1957 by half. Moreover, compound **14** prevented the growth of *E. coli* ATCC10536 with an MIC value of 0.0625 mg·mL^−1^, which was the lowest concentration among all obtained results. This proved that 3′-amino-4-benzyloxychalcone (**14**) could be a selective antibacterial agent against this bacterial strain, eight times more effective than the reference substance—oxytetracycline.

In the case of *A. alternata* CBS1526, the strongest inhibition was observed for 2′-, 3′-, 4′-aminochalcones **1**–**3**, 4′-amino-4-ethylchalcone (**6**), 2′-amino-4-carboxychalcone (**7**) and 3′-amino- 4-benzyloxy-3-methoxychalcone (**17**). The lowest MIC values were determined for compounds **2**, **3** and **7** (MIC = 0.5 mg·mL^−1^), which were two times lower than for nystatin.

Zhang et al. investigated the antifungal activities of chalcones with 2′-substituents, including 2′-aminochalcones, against the dermatophyte *Trichophyton rubrum*. Among them, 2′-amino-3-nitro- chalcone and 2′-amino-4-nitrochalcone (**10**) showed the highest activity with MIC_80_ values of 0.5 µg·mL^−1^, which were only two times weaker than the standard—fluconazole (MIC_80_ = 0.25 µg·mL^−1^) [33]. In our studies, 4′-aminochalcone (**3**), amino-nitrochalcones **10**–**12** and 2′-amino-4- benzyloxy-3-methoxychalcone (**16**) prevented the growth of *F. linii* KB-F1 (∆OD = 0). Moreover, among all tested aminochalcones **10**–**12** with -NO_2_ moieties attached at the C-4 position of ring B, the best results were obtained in the presence of 2′-amino-4-nitrochalcone (**10**) and 3′-amino-4- nitrochalcone (**11**), with MIC values of 0.5 mg·mL^−1^ for both derivatives. However, the minimal inhibitory concentration of 4′-amino-4-nitrochalcone (**12**) was two times higher in comparison to compounds **10** and **11**. These results proved that the position of the -NH_2_ group attached at ring A has a significant impact on the biological activity of aminochalcones. Also amino-carboxychalcones **7**–**9** hindered the growth of *F. linii* KB-F1 by 2–4 fold in comparison to the untreated control. Interestingly, the double extended lag-phase was observed only for 2′-amino-4-carboxychalcone (**7**).

In the case of *A. niger* DSM1957, five compounds (**1**, **3**, **7**, **12** and **17**) caused a complete growth inhibition. Their activity was stronger than that of the reference substance (∆OD = 0.74). Among them, 2′-aminochalcone (**1**) and 2′-amino-4-carboxychalcone (**7**) had the higher activity, with MIC values of 0.125 mg·mL^−1^, which indicated they were two times more effective than nystatin. Incubations with other aminochalcones resulted in elongation of the lag-phase by up to 40 h. Simultaneously, in comparison to the control culture, even a 4-fold decrease of optical density value was observed.

## 3. Materials and Methods

### 3.1. Chemicals

Reagents for synthesis: 2′-aminoacetophenone, 3′-aminoacetophenone, 4′-aminoacetophenone, benzaldehyde, 4-ethylbenzaldehyde, 4-formylbenzoic acid, 4-nitrobenzaldehyde, 4-(benzyloxy)- benzaldehyde, 4-(benzyloxy)-3-methoxy-benzaldehyde and sodium hydroxide were purchased from Sigma Aldrich (St. Louis, MO, USA).

### 3.2. Analysis

The progress of reactions was followed by thin layer chromatography (TLC) on silica gel-coated aluminum sheets with a fluorescent indicator (DC-Alufolien, Kieselgel 60 F_254_; Merck, Darmstadt, Germany). The TLC plates were sprayed with a solution of 1% Ce(SO_4_)_2_ and 2% H_3_[P(Mo_3_O_10_)_4_] in 5% H_2_SO_4_ and heated to visualize synthesized compounds. The crude products were purified by liquid column chromatography filled with silica gel (Kieselgel 60, 230–400 mesh, Merck). The purity of the compounds was analysed by high performance liquid chromatography (HPLC) on a Waters 2690 system (Milford, MA, USA) equipped a with Waters 996 Photodiode Array Detector. The analysed samples were dissolved in methanol and were separated on a reverse-phase C-18 column (Phenomenex, Torrance, CA, USA, Kinetex 5u XB-C18 100A, 250 mm × 4.6 mm), thermostated at 28 °C. The analysed samples were kept at 12 °C. The mobile phase consisted of two eluents: A—1% HCOOH in H_2_O and B—1% HCOOH in MeCN. Elution gradient was started from 55% of eluent A to 45% of eluent B over 15 min and a flow rate was amounted 1.5 mL·min^−1^.

The structures of the obtained products were determined by ^1^H and ^13^C nuclear magnetic resonance (NMR). Compounds for NMR analysis were dissolved in deuterated solvents: acetone-d_6_ or dimethyl sulfoxide-d_6_. The spectra were recorded on a an Avance^TM^600 MHz spectrometer (Bruker, Billerica, MA, USA) (Supplementary Materials). The high-resolution ESI-MS spectra were measured on a Bruker ESI-Q-TOF Maxis Impact Mass Spectrometer. The direct infusion ESI-MS parameters were: the mass spectrometer was operated in positive ion mode with the potential between the spray needle and the orifice of 3.5 kV, a nebulizer pressure of 0.4 bar, and a drying gas flow rate of 3.0 L·min^−1^ at 200 °C. The sample flow rate was 3.0 µL·min^−1^. Ionization mass spectra were collected in a range of *m*/*z* 50–1250. Melting points (uncorrected) were determined on a Boetius apparatus (Jena, Germany). Infrared spectra were determined using a Tensor 27 FTIR spectrometer (Bruker) with ATR accessory with diamond crystal in the wavelength range 400–4000 cm^−1^ (Supplementary Materials). Partition coefficient (logP) was calculated by using the JSME Molecular Editor, ^©^Molinspiration Cheminformatics software (Novartis Institutes for BioMedical Research Inc. and Bruno Bienfait).

### 3.3. Synthesis of Aminochalcones ***1***–***8***

#### 3.3.1. Method I (Compounds **1**–**6**, **10**–**18**)

In a round bottomed flask 0.24 g (6.00 mmol) of sodium hydroxide was dissolved in 10 mL methanol and 10 mL dioxane. After that, an appropriate aminoacetophenone (3.67 mmol) and the corresponding benzaldehyde (3.67 mmol) were added. Reactions were performed in room temperature on a magnetic stirrer until complete conversion of the substrates. After that, the reaction mixtures were poured into ice water. Precipitated crystals were collected and purified by liquid column chromatography on silica gel using mixtures of hexane-acetone, hexane-ethyl acetate or hexane-ethyl acetate-methylene chloride as eluents. Compounds **4** and **6** were synthesized at 2.5 times smaller scale.

#### 3.3.2. Method II (Compounds **7**–**9**)

To 0.24 g (6.00 mmol) of sodium hydroxide dissolved in water (25 mL) and ethanol (10 mL), 4-formylbenzoic acid (3.67 mmol) was added. The resulting mixture was stirred in the ice-bath for 15 min and after that the corresponding aminoacetophenone (3.67 mmol) was added. The reaction was continued on a magnetic stirrer in room temperature until complete conversion of the substrates. After that, 1 M hydrochloric acid was added dropwise to the reaction mixtures until pH 6–7 was reached. Precipitated crystals were filtered on a Büchner funnel, washed with distilled water and dried under vacuum. Crude products **8** and **9** were purified by liquid column chromatography using a mixture of chloroform-methanol as eluent.

Spectroscopic data of all synthesized aminochalcones is described below:

*2′-Aminochalcone* (**1**), yield 71.4% (0.59 g), yellow solid, m.p. 68–69 °C, lit. 70–71 °C [34]. FTIR-ATR (cm^−1^): 3377.97, 3325.85, 3282.72, 1644.58, 1613.85, 1574.34, 1540.18, 1495.37, 1481.22, 1445.84, 1339.54, 1300.52, 1286.16, 1261.81, 1205.41, 1153.70, 1081.03, 1030.55, 1010.82, 997.22, 974.44, 854.19, 773.64, 736.01, 689.67, 660.81, 573.21, 555.57, 525.29, 509.08, 479.08, 427.87; ^1^H-NMR (600 MHz, acetone-*d*_6_) δ (ppm): 8.06 (dd, *J* = 8.2, 1.5 Hz, 1H, H-6′), 7.93 (d, *J* = 15.5 Hz, 1H, H-α), 7.82–7.79 (m, 2H, H-2, H-6), 7.72 (d, *J* = 15.5 Hz, 1H, H-β), 7.47–7.39 (m, 3H, H-3, H-4, H-5), 7.30–7.26 (m, 1H, H-4′), 7.13 (s, 2H, -NH_2_), 6.85 (dd, *J* = 8.4, 1.2 Hz, 1H, H-3′), 6.67–6.61 (m, 1H, H-5′); ^13^C-NMR (150 MHz, acetone-*d*_6_) δ (ppm): 191.61 (C=O), 153.05 (C-2′), 142.98 (C-β), 136.38 (C-1), 134.99 (C-4′), 132.01 (C-6′), 130.80 (C-4), 129.72 (C-3, C-5), 129.23 (C-2, C-6), 124.08 (C-α), 119.15 (C-1′), 117.90 (C-3′), 115.71 (C-5′); HR ESI-MS *m*/*z* calculated for C_15_H_14_NO [M + H]^+^ 224.1070, found [M + H]^+^ 224.1069.

*3′-Aminochalcone* (**2**), yield 53.4% (0.44 g), yellow solid, m.p. 121–124 °C, lit. 115–118 °C [35]. FTIR-ATR (cm^−1^): 3467.58, 3365.18, 2923.54, 1655.93, 1619.34, 1585.37, 1489.68, 1454.03, 1331.41, 1306.82, 1288.12, 1183.40, 1101.75, 1074.94, 1041.05, 992.90, 977.83, 918.50, 887.18, 874.56, 860.34, 758.17, 719.43, 688.09, 677.35, 563.60, 511.04, 488.38; ^1^H-NMR (600 MHz, acetone-*d*_6_) δ (ppm): 7.83–7.78 (m, 2H, H-2, H-6), 7.76 (d, *J* = 15.7 Hz, 1H, H-β), 7.73 (d, *J* = 15.7 Hz, 1H, H-α), 7.49–7.42 (m, 3H, H-3, H-4, H-5), 7.43–7.38 (m, 1H, H-2′), 7.38 (ddd, *J* = 7.6, 1.7, 1.0 Hz, 1H, H-6′), 7.26–7.20 (m, 1H, H-5′), 6.94 (ddd, *J* = 7.9, 2.4, 1.0 Hz, 1H, H-4′), 4.91 (s, 2H, -NH_2_); ^13^C-NMR (150 MHz, acetone-*d*_6_) δ (ppm): 190.25 (C=O), 149.81 (C-3′), 144.11 (C-β), 140.03 (C-1′), 136.15 (C-1), 131.13 (C-4), 130.04 (C-5′), 129.79 (C-3, C-5), 129.34 (C-2, C-6), 123.34 (C-α), 119.55 (C-4′), 117.84 (C-6′), 114.46 (C-2′); HR ESI-MS *m*/*z* calculated for C_15_H_14_NO [M + H]^+^ 224.1070, found [M + H]^+^ 224.1072.

*4′-Aminochalcone* (**3**), yield 65.5% (0.54 g), yellow solid, m.p. 92–94 °C, lit. 90–92 °C [25]. FTIR-ATR (cm^−1^): 3328.68, 3218.16, 1625.81, 1602.80, 1575.64, 1541.66, 1516.96, 1494.00, 1446.84, 1339.18, 1302.12, 1286.15, 1227.37, 1172.18, 1134.22, 1030.61, 1018.78, 997.56, 971.61, 829.45, 766.37, 733.12, 694.83, 673.12, 638.13, 619.11, 559.57, 521.60, 501.64, 479.66, 421.47; ^1^H-NMR (600 MHz, acetone-*d*_6_) δ (ppm): 8.01–7.94 (m, 2H, H-2′, H-6′), 7.84 (d, *J* = 15.6 Hz, 1H, H-α), 7.80–7.77 (m, 2H, H-2, H-6), 7.71 (d, *J* = 15.6 Hz, 1H, H-β), 7.47–7.37 (m, 3H, H-3, H-4, H-5), 6.80–6.72 (m, 2H, H-3′, H-5′), 5.57 (s, 2H, -NH_2_); ^13^C-NMR (150 MHz, acetone-*d*_6_) δ (ppm): 187.03 (C=O), 154.30 (C-4′), 142.52 (C-β), 136.51 (C-1), 131.79 (C-2′, C-6′), 130.67 (C-4), 129.69 (C-3, C-5), 129.14 (C-2, C-6), 127.76 (C-1′), 123.14 (C-α), 114.01 (C-3′, C-5′); HR ESI-MS *m*/*z* calculated for C_15_H_14_NO [M + H]^+^ 224.1070, found [M + H]^+^ 224.1074.

*2′-Amino-4-ethylchalcone* (**4**), yield 72.8% (0.27 g), yellow solid, m.p. 34–36 °C. FTIR-ATR (cm^−1^): 3484.35, 3334.87, 2962.18, 2927.8, 2868.20, 1636.56, 1611.41, 1563.57, 1533.53, 1510.28, 1478.79, 1441.51, 1418.43, 1348.67, 1332.47, 1287.79, 1261.38, 1206.76, 1181.12, 1156.39, 1061.64, 1000.90, 990.92, 972.77, 867.49, 841.81, 826.64, 772.55, 744.57, 682.33, 655.83, 565.38, 526.32, 501.57; ^1^H-NMR (600 MHz, acetone-*d*_6_) δ (ppm): 8.05 (dd, *J* = 8.2, 1.3 Hz, 1H, H-6′), 7.88 (d, *J* = 15.5 Hz, 1H, H-α), 7.73–7.69 (m, 3H, H-2, H-6, H-β), 7.32–7.24 (m, 3H, H-3, H-5, H-4′), 7.11 (s, 2H, -NH_2_), 6.84 (dd, *J* = 8.3, 1.3 Hz, 1H, H-3′), 6.66–6.60 (m, 1H, H-5′), 2.67 (q, *J* = 7.6 Hz, 2H, -CH_2_-CH_3_), 1.23 (t, *J* = 7.6 Hz, 3H, -CH_3_); ^13^C-NMR (150 MHz, acetone-*d*_6_) δ (ppm): 191.68 (C=O), 153.00 (C-2′), 147.49 (C-4), 143.10 (C-β), 134.89 (C-4′), 133.91 (C-1), 131.94 (C-6′), 129.39 (C-2, C-6), 129.24 (C-3, C-5), 123.08 (C-α), 119.26 (C-1′), 117.89 (C-3′), 115.70 (C-5′), 29.32 (-CH_2_-CH_3_), 15.84 (-CH_3_); HR ESI-MS *m*/*z* calculated for C_17_H_18_NO [M + H]^+^ 252.1383, found [M + H]^+^ 252.1388.

*3′-Amino-4-ethylchalcone* (**5**), yield 47.9% (0.45 g), yellow solid, m.p. 69–71 °C. FTIR-ATR (cm^−1^): 3447.32, 3358.78, 2964.59, 2929.94, 1660.30, 1646.31, 1620.74, 1578.10, 1508.68, 1488.31, 1455.59, 1416.74, 1329.17, 1289.15, 1204.55, 1178.87, 1043.10, 992.38, 976.26, 951.87, 921.46, 869.62, 822.57, 776.37, 742.39, 717.24, 675.97, 653.54, 551.26, 534.59, 489.12, 423.57; ^1^H-NMR (600 MHz, acetone-*d*_6_) δ (ppm): 7.75–7.68 (m, 4H, H-2, H-6, H-α, H-β), 7.43–7.39 (m, 1H, H-2′), 7.40–7.34 (m, 1H, H-6′), 7.32–7.27 (m, 2H, H-3, H-5), 7.23 (t, *J* = 7.8 Hz, 1H, H-5′), 6.96–6.91 (m, 1H, H-4′), 4.91 (s, 2H, -NH_2_), 2.67 (q, *J* = 7.6 Hz, 2H, -CH_2_-CH_3_), 1.22 (t, *J* = 7.6 Hz, 3H, -CH_3_); ^13^C-NMR (150 MHz, acetone-*d*_6_) δ (ppm): 190.27 (C=O), 149.75 (C-3′), 147.85 (C-4), 144.22 (C-β), 140.14 (C-1′), 133.64 (C-1), 130.00 (C-5′), 129.47 (C-2, C-6), 129.28 (C-3, C-5), 122.34 (C-α), 119.46 (C-4′), 117.80 (C-6′), 114.45 (C-2′), 29.32 (-CH_2_-CH_3_), 15.80 (-CH_3_); HR ESI-MS *m*/*z* calculated for C_17_H_18_NO [M + H]^+^ 252.1383, found [M + H]^+^ 252.1391.

*4′-Amino-4-ethylchalcone* (**6**), yield 70.8% (0.26 g), yellow solid, m.p. 125–125 °C. FTIR-ATR (cm^−1^): 3465.95, 3334.06, 3217.87, 2961.76, 2928.20, 1628.78, 1596.43, 1579.38, 1556.72, 1509.02, 1457.23, 1437.83, 1417.82, 1336.26, 1303.40, 1288.36, 1224.09, 1171.45, 1119.89, 1018.06, 992.87, 814.32, 752.79, 731.09, 680.00, 636.54, 608.50, 550.12, 497.23, 419.01; ^1^H-NMR (600 MHz, acetone-*d*_6_) δ (ppm): 8.00–7.94 (m, 2H, H-2′, H-6′), 7.79 (d, *J* = 15.5 Hz, 1H, H-α), 7.72–7.67 (m, 3H, H-2, H-6, H-β), 7.32–7.25 (m, 2H, H-3, H-5), 6.79–6.71 (m, 2H, H-3′, H-5′), 5.55 (s, 2H, -NH_2_), 2.67 (q, *J* = 7.6 Hz, 2H, -CH_2_-CH_3_), 1.22 (t, *J* = 7.6 Hz, 3H, -CH_3_); ^13^C-NMR (150 MHz, acetone-*d*_6_) δ (ppm): 187.08 (C=O), 154.21 (C-4′), 147.32 (C-4), 142.60 (C-β), 134.04 (C-1), 131.72 (C-2′, C-6′), 129.29 (C-2, C-6), 129.21 (C-3, C-5), 127.89 (C-1′), 122.16 (C-α), 114.01 (C-3′, C-5′), 29.31 (-CH_2_-CH_3_), 15.84 (-CH_3_); HR ESI-MS *m*/*z* calculated for C_17_H_18_NO [M + H]^+^ 252.1383, found [M + H]^+^ 252.1391.

*2′-Amino-4-carboxychalcone* (**7**), yield 70.0% (0.69 g), yellow solid, m.p. 188–191 °C. FTIR-ATR (cm^−1^): 2980.74, 2883.94, 1688.08, 1649.77, 1610.72, 1576.61, 1540.98, 1508.41, 1483.29, 1422.81, 1321.34, 1287.94, 1263.36, 1211.48, 1159.09, 1126.15, 1112.59, 1013.62, 980.03, 964.25, 930.50, 852.74, 782.57, 751.37, 700.21, 664.52, 624.92, 548.47, 524.65, 485.60, 444.08, 419.91; ^1^H-NMR (600 MHz, DMSO-*d*_6_) δ (ppm): 13.10 (s, 1H, -COOH), 8.10 (dd, *J* = 8.3, 1.5 Hz, 1H, H-6′), 8.06 (d, *J* = 15.5 Hz, 1H, H-α), 8.01–7.94 (m, 4H, H-2, H-3, H-5, H-6), 7.66 (d, *J* = 15.5 Hz, 1H, H-β), 7.44 (s, 2H, -NH_2_), 7.32–7.26 (m, 1H, H-4′), 6.81 (dd, *J* = 8.4, 1.2 Hz, 1H, H-3′), 6.62–6.56 (m, 1H, H-5′); ^13^C-NMR (150 MHz, DMSO-*d*_6_) δ (ppm): 190.27 (C=O), 166.93 (-COOH), 152.20 (C-2′), 140.49 (C-β), 139.22 (C-1), 134.51 (C-4′), 131.65 (C-4), 131.53 (C-6′), 129.71 (C-3, C-5), 128.63 (C-2, C-6), 125.65 (C-α), 117.32 (C-1′), 116.94 (C-3′), 114.50 (C-5′); HR ESI-MS *m*/*z* calculated for C_16_H_14_NO_3_ [M + H]^+^ 268.0968, found [M + H]^+^ 268.0975.

*3′-Amino-4-carboxychalcone* (**8**), yield 27.2% (0.27 g), yellow solid, m.p. decomposition > 200 °C. FTIR-ATR (cm^−1^): 2922.41, 1676.92, 1656.20, 1591.50, 1566.88, 1541.38, 1508.17, 1489.29, 1455.63, 1419.46, 1324.25, 1295.40, 1181.20, 1039.90, 992.10, 962.87, 880.80, 851.20, 799.93, 773.12, 757.12, 728.41, 692.02, 676.85, 620.45, 551.66, 503.42, 486.51, 456.88, 419.46; ^1^H-NMR (600 MHz, DMSO-*d*_6_) δ (ppm): 7.99 (d, *J* = 8.4 Hz, 2H, H-3, H-5), 7.96–7.92 (m, 2H, H-2, H-6), 7.88 (d, *J* = 15.7 Hz, 1H, H-α), 7.72 (d, *J* = 15.7 Hz, 1H, H-β), 7.37–7.32 (m, 1H, H-6′), 7.28 (t, *J* = 2.0 Hz, 1H, H-2′), 7.21 (t, *J* = 7.8 Hz, 1H, H-5′), 6.86 (ddd, *J* = 8.0, 2.4, 0.9 Hz, 1H, H-4′), 5.49 (s, 2H, -NH_2_); ^13^C-NMR (150 MHz, DMSO-*d*_6_) δ (ppm): 189.54 (C=O), 167.29 (-COOH), 149.21 (C-3′), 142.03 (C-β), 138.47 (C-1′), 138.23 (C-1), 133.15 (C-4), 129.74 (C-3, C-5), 129.26 (C-5′), 128.65 (C-2, C-6), 124.38 (C-α), 118.84 (C-4′), 116.50 (C-6′), 112.99 (C-2′); HR ESI-MS *m*/*z* calculated for C_16_H_14_NO_3_ [M + H]^+^ 268.0968, found [M + H]^+^ 268.0971.

*4′-Amino-4-carboxychalcone* (**9**), yield 78.8% (0.78 g), yellow solid, m.p. decomposition > 200 °C. FTIR-ATR (cm^−1^): 1652.41, 1636.15, 1606.86, 1582.74, 1558.26, 1541.17, 1522.22, 1508.32, 1417.78, 1397.72, 1362.63, 1339.42, 1317.64, 1284.60, 1226.77, 1175.68, 1077.07, 1024.67, 973.97, 827.35, 779.52, 699.74, 664.81, 636.17, 594.70, 539.31, 498.02, 443.93, 418.55; ^1^H-NMR (600 MHz, DMSO-*d*_6_) δ (ppm): 8.00 (d, *J* = 8.0 Hz, 2H, H-3, H-5), 7.97–7.90 (m, 3H, H-2′, H-6′, H-α), 7.87 (d, *J* = 8.0 Hz, 2H, H-2, H-6), 7.63 (d, *J* = 15.5 Hz, 1H, H-β), 6.64 (d, *J* = 8.5 Hz, 2H, H-3′, H-5′), 6.26 (s, 2H, -NH_2_); ^13^C-NMR (150 MHz, DMSO-*d*_6_) δ (ppm): 185.74 (C=O), 154.11 (-COOH), 140.60 (C-β), 137.71 (C-4′), 131.23 (C-1, C-2′, C-6′), 129.77 (C-1′, C-3, C-5), 128.10 (C-2, C-6), 125.17 (C-4), 123.70 (C-α), 112.78 (C-3′, C-5′); HR ESI-MS *m*/*z* calculated for C_16_H_14_NO_3_ [M + H]^+^ 268.0968, found [M + H]^+^ 268.0972.

*2′-Amino-4-nitrochalcone* (**10**), yield 77.9% (0.77 g), orange solid, m.p. 114–117 °C. FTIR-ATR (cm^−1^): 3458.66, 3336.15, 1646.30, 1615.73, 1577.18, 1542.17, 1509.30, 1480.24, 1447.16, 1338.57, 1282.27, 1260.70, 1205.19, 1167.95, 1108.82, 1005.11, 985.97, 852.37, 771.81, 740.52, 676.50, 660.28, 633.89, 563.69, 538.21, 524.51, 481.80, 440.76; ^1^H-NMR (600 MHz, DMSO-*d*_6_) δ (ppm): 8.28–8.23 (m, 2H, H-3, H-5), 8.17–8.09 (m, 4H, H-2, H-6, H-6′, H-α), 7.69 (d, *J* = 15.5 Hz, 1H, H-β), 7.48 (s, 2H, -NH_2_), 7.33–7.27 (m, 1H, H-4′), 6.82 (dd, *J* = 8.5, 1.2 Hz, 1H, H-3′), 6.63–6.57 (m, 1H, H-5′); ^13^C-NMR (150 MHz, DMSO-*d*_6_) δ (ppm): 189.98 (C=O), 152.30 (C-2′), 147.73 (C-4), 141.64 (C-1), 139.07 (C-β), 134.65 (C-4′), 131.60 (C-6′), 129.56 (C-2, C-6), 127.58 (C-α), 123.88 (C-3, C-5), 117.16 (C-1′), 116.94 (C-3′), 114.47 (C-5′); HR ESI-MS *m*/*z* calculated for C_15_H_13_N_2_O_3_ [M + H]^+^ 269.0921, found [M + H]^+^ 269.0926.

*3′-Amino-4-nitrochalcone* (**11**), yield 55.1% (0.55 g), orange solid, m.p. 166–168 °C. FTIR-ATR (cm^−1^): 3425.98, 3353.4, 1658.08, 1578.12, 1541.87, 1508.46, 1488.46, 1455.75, 1412.60, 1332.24, 1316.38, 1289.71, 1186.59, 1106.99, 1076.88, 1040.63, 979.55, 968.98, 943.39, 920.05, 881.78, 869.10, 835.22, 789.52, 767.13, 749.70, 715.47, 678.11, 620.66, 533.29, 476.30, 451.08, 420.64; ^1^H-NMR (600 MHz, DMSO-*d*_6_) δ (ppm): 8.30–8.25 (m, 2H, H-3, H-5), 8.15–8.11 (m, 2H, H-2, H-6), 7.99 (d, *J* = 15.7 Hz, 1H, H-α), 7.76 (d, *J* = 15.7 Hz, 1H, H-β), 7.38 (ddd, *J* = 7.6, 1.7, 0.9 Hz, 1H, H-6′), 7.30–7.26 (m, 1H, H-2′), 7.22 (t, *J* = 7.8 Hz, 1H, H-5′), 6.87 (ddd, *J* = 8.0, 2.4, 0.9 Hz, 1H, H-4′), 5.40 (s, 2H, -NH_2_); ^13^C-NMR (150 MHz, DMSO-*d*_6_) δ (ppm): 189.40 (C=O), 149.24 (C-3′), 147.99 (C-4), 141.30 (C-1), 140.44 (C-β), 137.98 (C-1′), 129.71 (C-2, C-6), 129.26 (C-5′), 126.49 (C-α), 123.94 (C-3, C-5), 119.04 (C-4′), 116.65 (C-6′), 112.89 (C-2′); HR ESI-MS *m*/*z* calculated for C_15_H_13_N_2_O_3_ [M + H]^+^ 269.0921, found [M + H]^+^ 269.0927.

*4′-Amino-4-nitrochalcone* (**12**), yield 71.0% (0.70 g), orange solid, m.p. 205–207 °C, lit. 206–207 °C [36]. FTIR-ATR (cm^−1^): 3467.21, 3377.82, 1638.35, 1601.18, 1581.43, 1560.34, 1508.85, 1442.14, 1413.84, 1344.40, 1316.83, 1289.13, 1233.98, 1180.20, 1135.16, 1110.55, 1017.59, 1003.98, 987.79, 967.72, 853.58, 829.10, 780.21, 758.82, 734.39, 697.42, 666.20, 634.07, 599.08, 541.89, 479.30, 412.79; ^1^H-NMR (600 MHz, DMSO-*d*_6_) δ (ppm): 8.27–8.23 (m, 2H, H-3, H-5), 8.14–8.10 (m, 2H, H-2, H-6), 8.06 (d, *J* = 15.6 Hz, 1H, H-α), 7.98–7.93 (m, 2H, H-2′, H-6′), 7.69 (d, *J* = 15.6 Hz, 1H, H-β), 6.67–6.61 (m, 2H, H-3′, H-5′), 6.25 (s, 2H, -NH_2_); ^13^C-NMR (150 MHz, DMSO-*d*_6_) δ (ppm): 185.39 (C=O), 154.25 (C-4′), 147.68 (C-4), 141.81 (C-1), 138.63 (C-β), 131.42 (C-2′, C-6′), 129.46 (C-2, C-6), 126.67 (C-α), 124.99 (C-1′), 123.89 (C-3, C-5), 112.77 (C-3′, C-5′); HR ESI-MS *m*/*z* calculated for C_15_H_13_N_2_O_3_ [M + H]^+^ 269.0921, found [M + H]^+^ 269.0929.

*2′-Amino-4-benzyloxychalcone* (**13**), yield 88.6% (1.08 g), yellow solid, m.p. 89–91 °C. FTIR-ATR (cm^−1^): 1646.91, 1612.83, 1570.88, 1541.84, 1508.20, 1483.15, 1454.26, 1419.95, 1379.69, 1350.66, 1337.33, 1288.39, 1265.74, 1243.87, 1207.27, 1174.49, 1155.63, 1115.90, 1079.11, 1006.35, 993.30, 916.28, 861.16, 848.14, 833.92, 809.90, 765.23, 735.03, 694.17, 679.03, 658.83, 637.27, 609.25, 515.69, 419.10; ^1^H-NMR (600 MHz, acetone-*d*_6_) δ (ppm): 8.05 (dd, *J* = 8.1, 1.5 Hz, 1H, H-6′), 7.80 (d, *J* = 15.4 Hz, 1H, H-α), 7.78–7.74 (m, 2H, H-2, H-6), 7.70 (d, *J* = 15.4 Hz, 1H, H-β), 7.53–7.45 (m, 2H, H-2″, H-6″), 7.43–7.37 (m, 2H, H-3″, H-5″), 7.37–7.30 (m, 1H, H-4″), 7.30–7.23 (m, 1H, H-4′), 7.21–6.90 (m, 4H, H-3, H-5, -NH_2_), 6.83 (dd, *J* = 8.4, 1.1 Hz, 1H, H-3′), 6.67–6.58 (m, 1H, H-5′), 5.19 (s, 2H, -O-CH_2_-); ^13^C-NMR (150 MHz, acetone-*d*_6_) δ (ppm): 191.66 (C=O), 161.42 (C-4), 152.93 (C-2′), 142.89 (C-β), 137.98 (C-1″), 134.77 (C-4′), 131.88 (C-6′), 130.99 (C-2, C-6), 129.31 (C-3″, C-5″), 129.21 (C-1), 128.73 (C-4″), 128.47 (C-2″, C-6″), 121.72 (C-α), 119.39 (C-1′), 117.87 (C-3′), 116.05 (C-3, C-5), 115.68 (C-5′), 70.56 (-O-CH_2_-); HR ESI-MS *m*/*z* calculated for C_22_H_20_NO_2_ [M + H]^+^ 330.1489, found [M + H]^+^ 330.1501.

*3′-Amino-4-benzyloxychalcone* (**14**), yield 71.2% (0.87 g), yellow solid, m.p. 115–120 °C. FTIR-ATR (cm^−1^): 3450.90, 3359.11, 2970.58, 1739.03, 1655.55, 1628.42, 1588.24, 1571.05, 1509.14, 1490.46, 1455.41, 1421.22, 1380.61, 1339.93, 1311.31, 1288.24, 1252.87, 1205.51, 1170.44, 1079.94, 1038.87, 1013.49, 986.95, 919.05, 873.84, 837.55, 805.06, 785.36, 763.25, 744.73, 734.80, 700.21, 677.19, 636.81, 610.87, 519.12, 433.36; ^1^H-NMR (600 MHz, acetone-*d*_6_) δ (ppm): 7.78–7.73 (m, 2H, H-2, H-6), 7.72 (d, *J* = 15.6 Hz, 1H, H-β), 7.62 (d, *J* = 15.6 Hz, 1H, H-α), 7.52–7.46 (m, 2H, H-2″, H-6″), 7.44–7.38 (m, 3H, H-2′, H-3″, H-5″), 7.37 (ddd, *J* = 7.6, 1.7, 1.0 Hz, 1H, H-6′), 7.35–7.32 (m, 1H, H-4″), 7.22 (t, *J* = 7.7 Hz, 1H, H-5′), 7.12–7.06 (m, 2H, H-3, H-5), 6.93 (ddd, *J* = 7.9, 2.4, 1.0 Hz, 1H, H-4′), 5.19 (s, 2H, -O-CH_2_-), 4.89 (s, 2H, -NH_2_); ^13^C-NMR (150 MHz, acetone-*d*_6_) δ (ppm): 190.17 (C=O), 161.64 (C-4), 149.71 (C-3′), 143.99 (C-β), 140.30 (C-1′), 137.92 (C-1″), 131.11 (C-2, C-6), 129.97 (C-5′), 129.32 (C-3″, C-5″), 128.94 (C-1), 128.75 (C-4″), 128.48 (C-2″,C-6″), 121.00 (C-α), 119.34 (C-4′), 117.76 (C-6′), 116.10 (C-3, C-5), 114.44 (C-2′), 70.57 (-O-CH_2_-); HR ESI-MS *m*/*z* calculated for C_22_H_20_NO_2_ [M + H]^+^ 330.1489, found [M + H]^+^ 330.1494.

*4′-Amino-4-benzyloxychalcone* (**15**), yield 21.5% (0.26 g), yellow solid, m.p. 128–131 °C. FTIR-ATR (cm^−1^): 3471.33, 3375.40, 3336.84, 1646.24, 1620.54, 1597.14, 1583.39, 1570.48, 1557.08, 1508.12, 1453.58, 1438.61, 1421.51, 1382.48, 1348.70, 1313.09, 1290.93, 1257.82, 1220.24, 1170.16, 1133.07, 1080.19, 1025.84, 989.25, 818.93, 734.80, 696.30, 680.81, 637.97, 625.64, 597.14, 540.67, 517.95, 494.69, 412.69; ^1^H-NMR (600 MHz, acetone-*d*_6_) δ (ppm): 7.98–7.94 (m, 2H, H-2′, H-6′), 7.77–7.73 (m, 2H, H-2, H-6), 7.72 (d, *J* = 15.5 Hz, 1H, H-α), 7.68 (d, *J* = 15.5 Hz, 1H, H-β), 7.52–7.47 (m, 2H, H-2″, H-6″), 7.43–7.37 (m, 2H, H-3″, H-5″), 7.37–7.30 (m, 1H, H-4″), 7.11–7.05 (m, 2H, H-3, H-5), 6.77–6.71 (m, 2H, H-3′, H-5′), 5.52 (s, 2H, -NH_2_), 5.18 (s, 2H, -O-CH_2_-); ^13^C-NMR (150 MHz, acetone-*d*_6_) δ (ppm): 187.03 (C=O), 161.31 (C-4), 154.09 (C-4′), 142.36 (C-β), 138.01 (C-1″), 131.65 (C-2′, C-6′), 130.86 (C-2, C-6), 129.35 (C-1), 129.31 (C-3″, C-5″), 128.73 (C-4″), 128.47 (C-2″, C-6″), 128.02 (C-1′), 120.83 (C-α), 116.03 (C-3, C-5), 113.99 (C-3′, C-5′), 70.55 (-O-CH_2_-); HR ESI-MS *m*/*z* calculated for C_22_H_20_NO_2_ [M + H]^+^ 330.1489, found [M + H]^+^ 330.1494.

*2′-Amino-4-benzyloxy-3-methoxychalcone* (**16**), yield 73.4% (0.98 g), yellow solid, m.p. 94–96 °C. FTIR-ATR (cm^−1^): 1748.36, 1643.61, 1610.51, 1573.59, 1541.47, 1507.85, 1475.96, 1449.99, 1438.81, 1418.98, 1396.47, 1380.93, 1305.43, 1255.22, 1229.51, 1203.47, 1154.88, 1143.52, 1076.24, 1033.12, 1023.47, 1003.5, 967.44, 904.14, 868.01, 846.46, 835.22, 800.7, 764.94, 732.05, 693.46, 660.11, 630.13, 615.46, 597.6, 552.26, 519.17, 501.56, 466.17, 419.96; ^1^H-NMR (600 MHz, acetone-*d*_6_) δ (ppm): 8.02 (dd, *J* = 8.2, 1.5 Hz, 1H, H-6′), 7.82 (d, *J* = 15.4 Hz, 1H, H-α), 7.69 (d, *J* = 15.4 Hz, 1H, H-β), 7.53–7.48 (m, 3H, H-2, H-2″, H-6″), 7.43–7.36 (m, 2H, H-3″, H-5″), 7.36–7.31 (m, 1H, H-4″), 7.30 (ddd, *J* = 8.2, 2.0, 0.6 Hz, 1H, H-6), 7.29–7.23 (m, 1H, H-4′), 7.13–7.05 (m, 3H, H-5, -NH_2_), 6.83 (dd, *J* = 8.3, 1.2 Hz, 1H, H-3′), 6.64–6.58 (m, 1H, H-5′), 5.18 (s, 2H, -O-CH_2_-), 3.91 (s, 3H, -O-CH_3_); ^13^C-NMR (150 MHz, acetone-*d*_6_) δ 191.69 (C=O), 152.92 (C-4), 151.34 (C-3), 150.96 (C-2′), 143.39 (C-β), 138.13 (C-1″), 134.75 (C-4′), 131.88 (C-6′), 129.67 (C-1), 129.25 (C-3″, C-5″), 128.68 (C-4″), 128.51 (C-2″, C-6″), 123.71 (C-6), 121.85 (C-α), 119.40 (C-1′), 117.86 (C-3′), 115.64 (C-5′), 114.36 (C-5), 111.86 (C-2), 71.19 (-O-CH_2_-), 56.28 (-O-CH_3_); HR ESI-MS *m*/*z* calculated for C_23_H_22_NO_3_ [M + H]^+^ 360.1594, found [M + H]^+^ 360.1601.

*3′-Amino-4-benzyloxy-3-methoxychalcone* (**17**), yield 56.4% (0.75 g), yellow solid, m.p. 38 °C. FTIR-ATR (cm^−1^): 3446.89, 3359.02, 3031.48, 2935.80, 1738.77, 1652.27, 1622.82, 1570.76, 1505.91, 1453.29, 1419.23, 1379.82, 1311.17, 1250.32, 1186.02, 1161.21, 1134.98, 1078.16, 1023.51, 991.84, 914.30, 843.29, 787.07, 734.08, 695.78, 679.72, 663.21, 626.50, 586.66, 550.42, 468.48, 425.64; ^1^H-NMR (600 MHz, acetone-*d*_6_) δ (ppm): 7.70 (d, *J* = 15.6 Hz, 1H, H-β), 7.64 (d, *J* = 15.6 Hz, 1H, H-α), 7.52–7.49 (m, 2H, H-2″, H-6″), 7.49 (d, *J* = 2.1 Hz, 1H, H-2), 7.43–7.36 (m, 3H, H-2′, H-3″, H-5″), 7.36–7.31 (m, 2H, H-6′, H-4″), 7.30 (ddd, *J* = 8.2, 2.1, 0.5 Hz, 1H, H-6), 7.21 (t, *J* = 7.8 Hz, 1H, H-5′), 7.09 (d, *J* = 8.3 Hz, 1H, H-5), 6.92 (ddd, *J* = 8.0, 2.4, 1.0 Hz, 1H, H-4′), 5.18 (s, 2H, -O-CH_2_-), 4.89 (s, 2H, -NH_2_), 3.91 (s, 3H, -O-CH_3_); ^13^C-NMR (150 MHz, acetone-*d*_6_) δ (ppm): 190.26 (C=O), 151.57 (C-4), 150.96 (C-3), 149.70 (C-3′), 144.50 (C-β), 140.34 (C-1′), 138.07 (C-1″), 129.94 (C-5′), 129.39 (C-1), 129.26 (C-3″, C-5″), 128.70 (C-4″), 128.52 (C-2″, C-6″), 123.84 (C-6), 121.19 (C-α), 119.34 (C-4′), 117.78 (C-6′), 114.40 (C-2′), 114.35 (C-5), 111.95 (C-2), 71.18 (-O-CH_2_-), 56.29 (-O-CH_3_); HR ESI-MS *m*/*z* calculated for C_23_H_22_NO_3_ [M + H]^+^ 360.1594, found [M + H]^+^ 360.1606.

*4′-Amino-4-benzyloxy-3-methoxychalcone* (**18**), yield 51.5% (0.69 g), yellow solid, m.p. 181–184 °C. FTIR-ATR (cm^−1^): 3377.26, 2970.42, 1738.59, 1649.85, 1625.86, 1581.76, 1559.96, 1513.66, 1456.72, 1440.93, 1415.56, 1375.66, 1345.90, 1316.69, 1295.71, 1257.10, 1234.23, 1215.19, 1176.88, 1131.86, 1018.94, 1003.29, 975.16, 922.53, 872.90, 855.89, 829.71, 812.35, 794.53, 747.84, 730.35, 698.60, 614.60, 588.78, 557.84, 504.01, 459.73; ^1^H-NMR (600 MHz, acetone-*d*_6_) δ (ppm): 7.96–7.92 (m, 2H, H-2′, H-6′), 7.73 (d, *J* = 15.5 Hz, 1H, H-α), 7.65 (d, *J* = 15.5 Hz, 1H, H-β), 7.53–7.48 (m, 2H, H-2″, H-6″), 7.49 (d, *J* = 2.0 Hz, 1H, H-2), 7.42–7.37 (m, 2H, H-3″, H-5″), 7.36–7.31 (m, 1H, H-4″), 7.30–7.27 (m, 1H, H-6), 7.09 (d, *J* = 8.3 Hz, 1H, H-5), 6.76–6.70 (m, 2H, H-3′, H-5′), 5.51 (s, 2H, -NH_2_), 5.19 (s, 2H, -O-CH_2_-), 3.91 (s, 3H, -O-CH_3_); ^13^C-NMR (150 MHz, acetone-*d*_6_) δ (ppm): 187.04 (C=O), 154.10 (C-4′), 151.23 (C-4), 150.99 (C-3), 142.84 (C-β), 138.20 (C-1″), 131.65 (C-2′, C-6′), 129.85 (C-1′), 129.27 (C-3″, C-5″), 128.69 (C-4″), 128.53 (C-2″, C-6″), 128.04 (C-1), 123.52 (C-6), 120.98 (C-α), 114.44 (C-5), 113.97 (C-3′, C-5′), 111.84 (C-2), 71.22 (-O-CH_2_-), 56.29 (-O-CH_3_); HR ESI-MS *m*/*z* calculated for C_23_H_22_NO_3_ [M + H]^+^ 360.1594, found [M + H]^+^ 360.1605.

### 3.4. Anticancer Activity

Anticancer activity was evaluated using the sulforhodamine B assay (SRB) on four different human colon cancer cell lines: HT-29, LS180, LoVo and LoVo/DX and green monkey kidney fibroblasts COS7 (ATCC, Manassas, VA, USA). Cell lines HT-29 and COS7 were cultured in α-MEM medium (IIET PAS, Wrocław, Poland), LS180 in OptiMEM medium (IIET PAS), LoVo and LoVo/DX in mixture of α-MEM and OptiMEM medium (1:1 (*v*:*v*)). Each culture medium was supplemented with 10% fetal bovine serum, 2 mM glutamine, 100 μg·mL^−1^ penicillin, 100 μg·mL^−1^ streptomycin and 0.25 μg·mL^−1^ amphotericin (all supplements purchased from Gibco (Paisley, UK). Cells were incubated at 37 °C, 5% CO_2_ in HeraCELL 150i incubator (Thermo Fisher Scientific, Waltham, MA, USA).

For the SRB assay, cells were seeded onto 96-well plates (Sarstedt, Nümbrecht, Germany) at a density of 5·10^3^/well and cultured overnight as described above. Then, the growth medium was gently removed and replaced with fresh medium, supplemented with aminochalcone derivatives in the concentration ranging from 1 µg·mL^−1^ to 100 µg·mL^−1^. All stock solutions were prepared in DMSO at a concentration of 10 mg·mL^−1^. Cells were incubated for 48 h and then the SRB assay was performed as described previously [30]. Each experiment was performed in quadruplicate. The final results were reported as the *IC*_50_ values calculated for three independent experiments. Cisplatin (Acros Organics, Waltham, MA, USA) and doxorubicin (Fisher BioReagents, Waltham, MA, USA) were used as positive controls of the test.

### 3.5. Antimicrobial Activity

#### 3.5.1. Microbial Growth Curve Calculation

The antimicrobial screening tests were performed on two strains of bacteria: *E. coli* ATCC10536 and *S. aureus* DSM799, strain of yeast *C. albicans* DSM1386 and three strains of fungi: *F. linii* KB-F1, *A. alternata* CBS1526 and *A. niger* DSM1957. All microorganisms were obtained from the collection of the Faculty of Biotechnology and Food Microbiology, Wrocław University of Environmental and Life Sciences. The bacteria were cultured in nutrient broth (Biocorp, Warsaw, Poland), yeast and fungi in YM medium (3 g yeast extract, 3 g malt extract, 5 g bacteriological peptone and 10 g of glucose dissolved in 1 L of distilled water). The tests were carried out in 100-well microtiter plates. To each well 280 μL of culture medium, 10 μL of microorganism suspension and 10 μL of aminochalcone (**1**–**18**) dissolved in dimethyl sulfoxide (3% (*w*/*v*)) were added. The final concentration of the tested compound (**1**–**18**) was 0.1% (*w*/*v*). Each experiment were performed in 3 replications. The optical density of the cell suspension was measured on Bioscreen C (Automated Growth Curve Analysis System Lab System, Vantaa, Finland) at 560 nm automatically, at regular intervals of 30 min for 2–3 days. Cell cultures were maintained at 28 °C on a continuous shaker. To prepare the growth curves for each strain, the mean values of the absorbance in a function of time were used. The resulting antimicrobial activity was expressed as the increase of optical density (ΔOD) and was compared to that of the control cultures in the medium supplemented with dimethyl sulfoxide and also with reference substances: oxytetracycline (against bacteria), cycloheximide (against yeast) and nystatin (against fungi) (Sigma Aldrich).

#### 3.5.2. Minimal Inhibitory Concentration (MIC) Evaluation

In all cases of complete growth inhibition in the screening tests (∆OD = 0), the minimal inhibitory concentration (MIC) values were evaluated using the broth method. Sterile 96-well microtiter plates (Sarstedt) with nutrient broth (for bacteria) (Biocorp) or YM medium (for yeast and filamentous fungi) were inoculated with a standardized cell suspension of bacteria and yeast or fungal spores of 10^5^ cell·mL^−1^, and supplemented with aminochalcones dissolved in DMSO. Final concentration of tested compounds in the incubation mixtures ranged from 0.1% to 0.00625% and was achieved with the series of two-fold dilutions. All analysis were conducted in triplicates. The plates were incubated at 30 °C (bacteria and yeast) or 25 °C (filamentous fungi) for 48–72 h. The results were measured with the Spark^®^ Multimode Microplate Reader (Tecan, Männedorf, Switzerland) at a 560 nm wavelength. The minimal inhibitory concentration was defined as the lowest concentration of a tested compound that completely restricted the growth of the microorganism. In order to verify the obtained results, microbial cultures subjected to each concentration of aminochalcone were inoculated onto nutrient agar or YM plates and incubated as described above. The lack of growth on agar plate was a confirmation of the MIC of the tested compound. The resulting MIC values were compared with the reference substances: oxytetracycline (against bacteria), cycloheximide (against yeast) and nystatin (against fungi) (Sigma Aldrich).

## 4. Conclusions

In this paper we describe the synthesis of a library of 18 aminochalcones with different electron-withdrawing and electron-donating substituents at the C-4 position. Among them, 10 compounds were novel, never previously described in the scientific literature.

We evaluated the anticancer and antimicrobial activity of all synthesized derivatives. The best results were observed for compounds **1**–**3** against the HT-29 human colon cancer cell line with activity expressed as the IC_50_ value below 2 µg·mL^−1^. This inhibition of proliferation was almost 12 times stronger in comparison to the reference substance–cisplatin (**19**) (IC_50_ = 16.73 µg·mL^−1^). Similar effects were observed for aminochalcones with nitro groups attached to the C-4 position (compounds **10**–**12**) against the LoVo and LoVo/DX cell lines (IC_50_ < 2 µg·mL^−1^). This proves that the presence of a strong electron withdrawing group -NO_2_ group in the *para* position as well as the aminochalcones without moieties attached to ring B significantly enhances the biological properties. However, these derivatives are characterized by a high toxicity against green monkey fibroblasts COS7. Based on the available knowledge concerning the mechanism of action of 2′-aminochalcone (**1**), we could assume that aminochalcones induce apoptosis rather than necrosis in colon cancer cell lines. However, to confirm this hypothesis with certainty, further research into the molecular mechanism of action is required and it will be taken into account in further studies. In the case of antimicrobial activity, complete inhibition of the growth of all tested microorganisms under 0.1% (*w*/*v*) concentration was observed for 4′-aminochalcone (**3**). Furthermore, sixteen of the synthesized derivatives (compounds **2**–**5**, **7**–**18**) prevented the growth of *E. coli* ATCC10536 and the best results were observed for novel 3′-amino-4-benzyloxychalcone (**14**) with an MIC value of 0.0625 mg·mL^−1^. The presence of an amino group in the *meta* position and the additional aromatic ring in compound **14**, which increases the hydrophobicity of the molecule, may facilitate the penetration into the microorganisms’ cells. It is worth mentioning that the obtained aminochalcones fulfill all Lipinski’s rules, which allows us to qualify them as potential drug candidates. Moreover, strong inhibition of proliferation against different human colon cancer cell lines and prevention of the microbial growth proved their potential applicability as anticancer and also antimicrobial agents.

## Figures and Tables

**Table 1:**
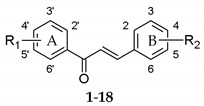
Aminochalcones **1**–**18** and their Lipiński’s rule of five parameters.

No.	Substituent on Ring A	Substituent on Ring B	MW	logP	nON	nOHNH
**1**	2′-NH_2_	4-H	223.28	3.25	2	2
**2**	3′-NH_2_	4-H	223.28	2.86	2	2
**3**	4′-NH_2_	4-H	223.28	2.89	2	2
**4**	2′-NH_2_	4-CH_2_CH_3_	251.33	4.16	2	2
**5**	3′-NH_2_	4-CH_2_CH_3_	251.33	3.78	2	2
**6**	4′-NH_2_	4-CH_2_CH_3_	251.33	3.80	2	2
**7**	2′-NH_2_	4-COOH	267.28	3.16	4	3
**8**	3′-NH_2_	4-COOH	267.28	2.77	4	3
**9**	4′-NH_2_	4-COOH	267.28	2.80	4	3
**10**	2′-NH_2_	4-NO_2_	268.27	3.21	5	2
**11**	3′-NH_2_	4-NO_2_	268.27	2.82	5	2
**12**	4′-NH_2_	4-NO_2_	268.27	2.85	5	2
**13**	2′-NH_2_	4-OCH_2_C_6_H_5_	329.40	4.90	3	2
**14**	3′-NH_2_	4-OCH_2_C_6_H_5_	329.40	4.51	3	2
**15**	4′-NH_2_	4-OCH_2_C_6_H_5_	329.40	4.54	3	2
**16**	2′-NH_2_	3-OMe-4-OCH_2_C_6_H_5_	359.43	4.49	4	2
**17**	3′-NH_2_	3-OMe-4-OCH_2_C_6_H_5_	359.43	4.11	4	2
**18**	4′-NH_2_	3-OMe-4-OCH_2_C_6_H_5_	359.43	4.13	4	2

MW: Molecular Weight; logP: logarithm of octanol-water partition coefficient; nON: number of hydrogen bond acceptors; nOHNH: number of hydrogen bond donors.

**Table 2:** Anticancer activity of aminochalcones **1**–**18** **(IC_50_, [µg·mL^−1^])**.

No.	Aminochalcone	HT-29	LS180	LoVo	LoVo/DX	COS7
**1**	2′-Aminochalcone	1.43 ± 0.16	2.06 ± 0.18	1.56 ± 0.04	1.43 ± 0.02	2.64 ± 0.47
**2**	3′-Aminochalcone	1.60 ± 0.01	2.13 ± 0.18	1.88 ± 0.12	1.97 ± 0.30	3.25 ± 0.66
**3**	4′-Aminochalcone	1.98 ± 0.18	4.68 ± 0.09	2.84 ± 0.09	2.26 ± 0.05	3.85 ± 0.28
**4**	2′-Amino-4-ethylchalcone	3.98 ± 0.03	5.10 ± 0.65	4.77 ± 0.37	4.78 ± 0.19	9.57 ± 0.52
**5**	3′-Amino-4-ethylchalcone	3.43 ± 0.05	4.16 ± 0.12	1.79 ± 0.10	2.35 ± 0.08	4.42 ± 0.27
**6**	4′-Amino-4-ethylchalcone	3.61 ± 0.21	5.20 ± 0.30	4.26 ± 0.19	3.68 ± 0.30	7.16 ± 0.34
**7**	2′-Amino-4-carboxychalcone	4.26 ± 0.13	7.86 ± 0.35	7.68 ± 0.34	11.08 ± 0.44	7.78 ± 0.75
**8**	3′-Amino-4-carboxychalcone	12.32 ± 0.32	9.22 ± 0.72	31.50 ± 0.13	27.44 ± 0.11	15.93 ± 1.04
**9**	4′-Amino-4-carboxychalcone	32.42 ± 0.99	18.37 ± 0.19	73.54 ± 0.98	66.18 ± 1.32	32.00 ± 8.11
**10**	2′-Amino-4-nitrochalcone	3.24 ± 0.27	2.00 ± 0.35	1.11 ± 0.07	0.96 ± 0.12	1.79 ± 0.14
**11**	3′-Amino-4-nitrochalcone	2.77 ± 0.49	2.89 ± 0.53	1.34 ± 0.10	1.36 ± 0.06	2.30 ± 0.45
**12**	4′-Amino-4-nitrochalcone	3.42 ± 0.54	4.64 ± 0.54	1.80 ± 0.10	2.00 ± 0.12	3.10 ± 0.50
**13**	2′-Amino-4-benzyloxychalcone	ND	ND	ND	ND	ND
**14**	3′-Amino-4-benzyloxychalcone	7.54 ± 0.18	6.51 ± 0.62	ND	5.19 ± 0.41	7.61 ± 0.43
**15**	4′-Amino-4-benzyloxychalcone	7.83 ± 0.14	8.23 ± 0.49	7.64 ± 0.53	6.66 ± 0.48	13.39 ± 2.78
**16**	2′-Amino-4-benzyloxy-3-methoxychalcone	4.85 ± 0.08	6.64 ± 0.29	5.77 ± 0.38	4.59 ± 0.21	9.02 ± 0.17
**17**	3′-Amino-4-benzyloxy-3-methoxychalcone	2.90 ± 0.27	3.76 ± 0.38	1.71 ± 0.13	1.77 ± 0.09	3.45 ± 0.22
**18**	4′-Amino-4-benzyloxy-3-methoxychalcone	5.01 ± 0.49	4.59 ± 0.29	3.54 ± 0.30	ND	ND
**19**	Cisplatin	16.73 ± 0.58	1.49 ± 0.13	2.09 ± 0.12	2.03 ± 0.17	3.86 ± 0.62
**20**	Doxorubicin	0.33 ± 0.02	0.05 ± 0.02	0.73 ± 0.18	5.32 ± 1.44	3.55 ± 1.15

ND—not detected in the tested concentration range.

**Table 3:** Antimicrobial activity of aminochalcones **1**–**18**.

Strain		*E. coli*	*S. aureus*	*C. albicans*	*A. alternata*	*F. linii*	*A. niger*
**Control**	Lag-phase [h]	4.0	2.5	3.0	16.5	13.5	11.0
	∆OD	1.61	1.67	1.58	1.86	1.95	2.14
**1**	Lag-phase [h]	10.0	-	24.5	-	18.5	-
	∆OD	0.12	0.0	0.14	0.0	0.73	0.0
**2**	Lag-phase [h]	-	-	-	-	30.0	1.5
	∆OD	0.0	0.0	0.0	0.0	0.23	0.34
**3**	Lag-phase [h]	-	-	-	-	-	-
	∆OD	0.0	0.0	0.0	0.0	0.0	0.0
**4**	Lag-phase [h]	-	32.5	6.0	26.5	7.5	30.0
	∆OD	0.0	0.84	0.98	0.40	1.04	0.60
**5**	Lag-phase [h]	-	29.5	9.5	12.0	8.0	25.5
	∆OD	0.0	0.98	0.61	0.39	0.80	0.36
**6**	Lag-phase [h]	3.5	28.0	9.5	-	9.0	36.5
	∆OD	0.16	0.87	0.44	0.0	0.40	0.41
**7**	Lag-phase [h]	-	-	-	-	26.0	-
	∆OD	0.0	0.0	0.0	0.0	0.37	0.0
**8**	Lag-phase [h]	-	-	23.0	26.0	13.0	39.0
	∆OD	0.0	0.0	0.28	1.14	0.45	1.11
**9**	Lag-phase [h]	-	37.0	8.5	12.5	13.5	35.0
	∆OD	0.0	0.35	0.97	1.12	0.99	0.51
**10**	Lag-phase [h]	-	11.5	-	0.5	-	23.0
	∆OD	0.0	0.15	0.0	0.20	0.0	0.79
**11**	Lag-phase [h]	-	-	-	42.0	-	39.5
	∆OD	0.0	0.0	0.0	0.55	0.0	0.83
**12**	Lag-phase [h]	-	-	12.0	13.0	-	-
	∆OD	0.0	0.0	0.13	0.87	0.0	0.0
**13**	Lag-phase [h]	-	-	-	13.0	16.0	21.5
	∆OD	0.0	0.0	0.0	0.92	0.32	0.78
**14**	Lag-phase [h]	-	33.5	4.5	12.0	6.0	20.5
	∆OD	0.0	0.82	1.42	0.63	0.87	0.98
**15**	Lag-phase [h]	-	8.0	16.0	19.5	11.5	25.0
	∆OD	0.0	0.22	0.37	1.04	0.50	0.57
**16**	Lag-phase [h]	-	-	8.5	8.5	-	40.5
	∆OD	0.0	0.0	0.66	0.64	0.0	0.41
**17**	Lag-phase [h]	-	33.5	9.0	-	11.0	-
	∆OD	0.0	0.36	0.55	0.0	0.24	0.0
**18**	Lag-phase [h]	-	33.5	4.5	21.5	6.5	28.5
	∆OD	0.0	0.26	0.68	0.22	0.41	0.65
**Oxytetracycline**	Lag-phase [h]	-	-	NA	NA	NA	NA
	∆OD	0.0	0.0				
**Cycloheximide**	Lag-phase [h]	NA	NA	-	NA	NA	NA
	∆OD			0.0			
**Nystatin**	Lag-phase [h]	NA	NA	NA	-	10.0	4.5
	∆OD				0.0	0.13	0.74

OD—Optical Density (measured for λ = 560 nm); “-“—not determined in view of ∆OD = 0; NA—not applicable.

**Table 4:** Minimal inhibitory concentrations [mg·mL^−1^] for aminochalcones **1**–**18**.

Compound	Strain					
	*E. coli*	*S. aureus*	*C. albicans*	*A. alternata*	*F. linii*	*A. niger*
**1**	-	0.25	-	1.0	-	0.125
**2**	0.25	0.25	0.5	0.5	-	-
**3**	0.5	0.25	0.5	0.5	0.25	0.5
**4**	0.5	-	-	-	-	-
**5**	1.0	-	-	-	-	-
**6**	-	-	-	1.0	-	-
**7**	0.125	0.125	0.125	0.5	-	0.125
**8**	0.125	0.125	-	-	-	-
**9**	0.125	-	-	-	-	-
**10**	0.25	-	0.5	-	0.5	-
**11**	1.0	0.25	0.25	-	0.5	1.0
**12**	0.25	0.25	-	-	1.0	-
**13**	0.25	0.25	0.5	-	-	-
**14**	0.0625	-	-	-	-	-
**15**	0.125	-	-	-	-	-
**16**	0.5	0.5	-	-	0.5	-
**17**	0.125	-	-	1.0	-	1.0
**18**	0.5	-	-	-	-	-
**Oxytetracycline**	0.5	0.125	NA	NA	NA	NA
**Cycloheximide**	NA	NA	0.5	NA	NA	NA
**Nystatin**	NA	NA	NA	1.0	0.5	0.25

“-“—not determined; NA—not applicable.

## Supplementary Materials

The following are available online. Figure S1: ^1^H-NMR (600 MHz, acetone-*d_6_*) spectrum of 2′-aminochalcone (**1**), Figure S2: ^13^C-NMR (150 MHz, acetone-*d_6_*) spectrum of 2′-aminochalcone (**1**), Figure S3: FTIR-ATR spectrum of 2′-aminochalcone (**1**), Figure S4: ^1^H-NMR (600 MHz, acetone-*d_6_*) spectrum of 3′-aminochalcone (**2**), Figure S5: ^13^C-NMR (150 MHz, acetone-*d_6_*) spectrum of 3′-aminochalcone (**2**), Figure S6: FTIR-ATR spectrum of 3′-aminochalcone (**2**), Figure S7: ^1^H-NMR (600 MHz, acetone-*d_6_*) spectrum of 4′-aminochalcone (**3**), Figure S8: ^13^C-NMR (150 MHz, acetone-*d_6_*) spectrum of 4′-aminochalcone (**3**), Figure S9: FTIR-ATR spectrum of 4′-aminochalcone (**3**), Figure S10: ^1^H-NMR (600 MHz, acetone-*d_6_*) spectrum of 2′-amino-4-ethylchalcone (**4**), Figure S11: ^13^C-NMR (150 MHz, acetone-*d_6_*) spectrum of 2′-amino-4-ethylchalcone (**4**), Figure S12: FTIR-ATR spectrum of 2′-amino-4-ethylchalcone (**4**), Figure S13: ^1^H-NMR (600 MHz, acetone-*d_6_*) spectrum of 3′-amino-4-ethylchalcone (**5**), Figure S14: ^13^C-NMR (150 MHz, acetone-*d_6_*) spectrum of 3′-amino-4-ethylchalcone (**5**), Figure S15: FTIR-ATR spectrum of 3′-amino-4-ethylchalcone (**5**), Figure S16: ^1^H-NMR (600 MHz, acetone-*d_6_*) spectrum of 4′-amino-4-ethylchalcone (**6**), Figure S17: ^13^C-NMR (150 MHz, acetone-*d_6_*) spectrum of 4′-amino-4-ethylchalcone (**6**), Figure S18: FTIR-ATR spectrum of 4′-amino-4-ethylchalcone (**6**), Figure S19: ^1^H-NMR (600 MHz, DMSO-*d_6_*) spectrum of 2′-amino-4-carboxychalcone (**7**), Figure S20: ^13^C-NMR (150 MHz, DMSO-*d_6_*) spectrum of 2′-amino-4-carboxychalcone (**7**), Figure S21: FTIR-ATR spectrum of 2′-amino-4-carboxychalcone (**7**), Figure S22: ^1^H-NMR (600 MHz, DMSO-*d_6_*) spectrum of 3′-amino-4-carboxychalcone (**8**), Figure S23: ^13^C-NMR (150 MHz, DMSO-*d_6_*) spectrum of 3′-amino-4-carboxychalcone (**8**), Figure S24: FTIR-ATR spectrum of 3′-amino-4-carboxychalcone (**8**), Figure S25: ^1^H-NMR (600 MHz, DMSO-*d_6_*) spectrum of 4′-amino-4-carboxychalcone (**9**), Figure S26: ^13^C-NMR (150 MHz, DMSO-*d_6_*) spectrum of 4′-amino-4-carboxychalcone (**9**), Figure S27: FTIR-ATR spectrum of 4′-amino-4-carboxychalcone (**9**), Figure S28: ^1^H-NMR (600 MHz, DMSO-*d_6_*) spectrum of 2′-amino-4-nitrochalcone (**10**), Figure S29: ^13^C-NMR (150 MHz, DMSO-*d_6_*) spectrum of 2′-amino-4-nitrochalcone (**10**), Figure S30: FTIR-ATR spectrum of 2′-amino-4-nitrochalcone (**10**), Figure S31: ^1^H-NMR (600 MHz, DMSO-*d_6_*) spectrum of 3′-amino-4-nitrochalcone (**11**), Figure S32: ^13^C-NMR (150 MHz, DMSO-*d_6_*) spectrum of 3′-amino-4-nitrochalcone (**11**), Figure S33: FTIR-ATR spectrum of 3′-amino-4-nitrochalcone (**11**), Figure S34: ^1^H-NMR (600 MHz, DMSO-*d_6_*) spectrum of 4′-amino-4-nitrochalcone (**12**), Figure S35: ^13^C-NMR (150 MHz, DMSO-*d_6_*) spectrum of 4′-amino-4-nitrochalcone (**12**), Figure S36: FTIR-ATR spectrum of 4′-amino-4-nitrochalcone (**12**), Figure S37: ^1^H-NMR (600 MHz, acetone-*d_6_*) spectrum of 2′-amino-4-benzyloxychalcone (**13**), Figure S38: ^13^C-NMR (150 MHz, acetone-*d_6_*) spectrum of 2′-amino-4-benzyloxychalcone (**13**), Figure S39: FTIR-ATR spectrum of 2′-amino-4-benzyloxychalcone (**13**), Figure S40: ^1^H-NMR (600 MHz, acetone-*d_6_*) spectrum of 3′-amino-4-benzyloxychalcone (**14**), Figure S41: ^13^C-NMR (150 MHz, acetone-*d_6_*) spectrum of 3′-amino-4-benzyloxychalcone (**14**), Figure S42: FTIR-ATR spectrum of 3′-amino-4-benzyloxychalcone (**14**), Figure S43: ^1^H-NMR (600 MHz, acetone-*d_6_*) spectrum of 4′-amino-4-benzyloxychalcone (**15**), Figure S44: ^13^C-NMR (150 MHz, acetone-*d_6_*) spectrum of 4′-amino-4-benzyloxychalcone (**15**), Figure S45: FTIR-ATR spectrum of 4′-amino-4-benzyloxychalcone (**15**), Figure S46: ^1^H-NMR (600 MHz, acetone-*d_6_*) spectrum of 2′-amino-4-benzyloxy-3-methoxychalcone (**16**), Figure S47: ^13^C-NMR (150 MHz, acetone-*d_6_*) spectrum of 2′-amino-4-benzyloxy-3-methoxychalcone (**16**), Figure S48: FTIR-ATR spectrum of 2′-amino-4-benzyloxy-3-methoxychalcone (**16**), Figure S49: ^1^H-NMR (600 MHz, acetone-*d_6_*) spectrum of 3′-amino-4-benzyloxy-3-methoxychalcone (**17**), Figure S50: ^13^C-NMR (150 MHz, acetone-*d_6_*) spectrum of 3′-amino-4-benzyloxy-3-methoxychalcone (**17**), Figure S51: FTIR-ATR spectrum of 3′-amino-4-benzyloxy-3-methoxychalcone (**17**), Figure S52: ^1^H-NMR (600 MHz, acetone-*d_6_*) spectrum of 4′-amino-4-benzyloxy-3-methoxychalcone (**18**), Figure S53: ^13^C-NMR (150 MHz, acetone-*d_6_*) spectrum of 4′-amino-4-benzyloxy-3-methoxychalcone (**18**), Figure S54: FTIR-ATR spectrum of 4′-amino-4-benzyloxy-3-methoxychalcone (**18**).
