# Supplementary material for: Synthesis and Biological Evaluation of Novel Aminochalcones as Potential Anticancer and Antimicrobial Agents
Source: Molecules. 2019 Nov 15;24(22):4129. doi: 10.3390/molecules24224129 (PMC6891756; doi:10.3390/molecules24224129)
Supplement: Supplementary file 1 [file molecules-24-04129-s001.pdf]

Synthesis and Biological Evaluation of Novel Aminochalcones as Potential Anticancer and Antimicrobial Agents

Joanna Kozłowska [1,*], Bartłomiej Potaniec [1,2], Dagmara Baczyńska [3], Barbara Żarowska [4] and Mirosław Anioł [1]

[1] Department of Chemistry, Wrocław University of Environmental and Life Sciences, Norwida 25, 50-375 Wrocław, Poland; b.potaniec@gmail.com (B.P.); miroslaw.aniol@upwr.edu.pl (M.A.)

[2] ŁUKASIEWICZ Research Network - PORT Polish Center for Technology Development, Stabłowicka 147, 54-066 Wrocław, Poland

[3] Department of Molecular and Cellular Biology, Faculty of Pharmacy with Division of Laboratory Diagnostics, Wroclaw Medical University, Borowska 211A, 50-556 Wrocław, Poland; dagmara.baczynska@umed.wroc.pl

[4] Department of Biotechnology and Food Microbiology, Wrocław University of Environmental and Life Sciences, Chełmońskiego 37, 51-630 Wrocław, Poland; barbara.zarowska@upwr.edu.pl

* Correspondence: joannakozlowska3@gmail.com

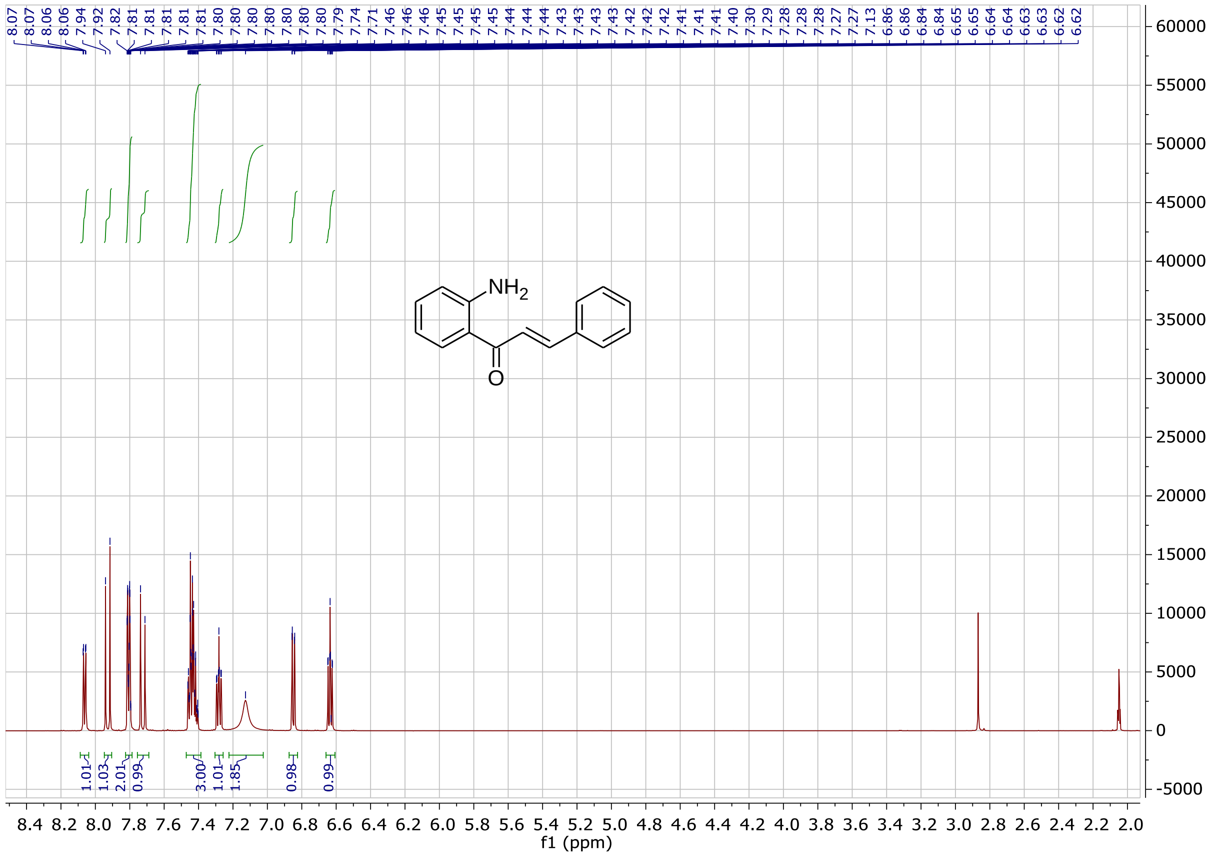

Figure S1. ^{1}H-NMR (600 MHz, acetone-d_6) spectrum of 2′-aminochalcone (**1**)

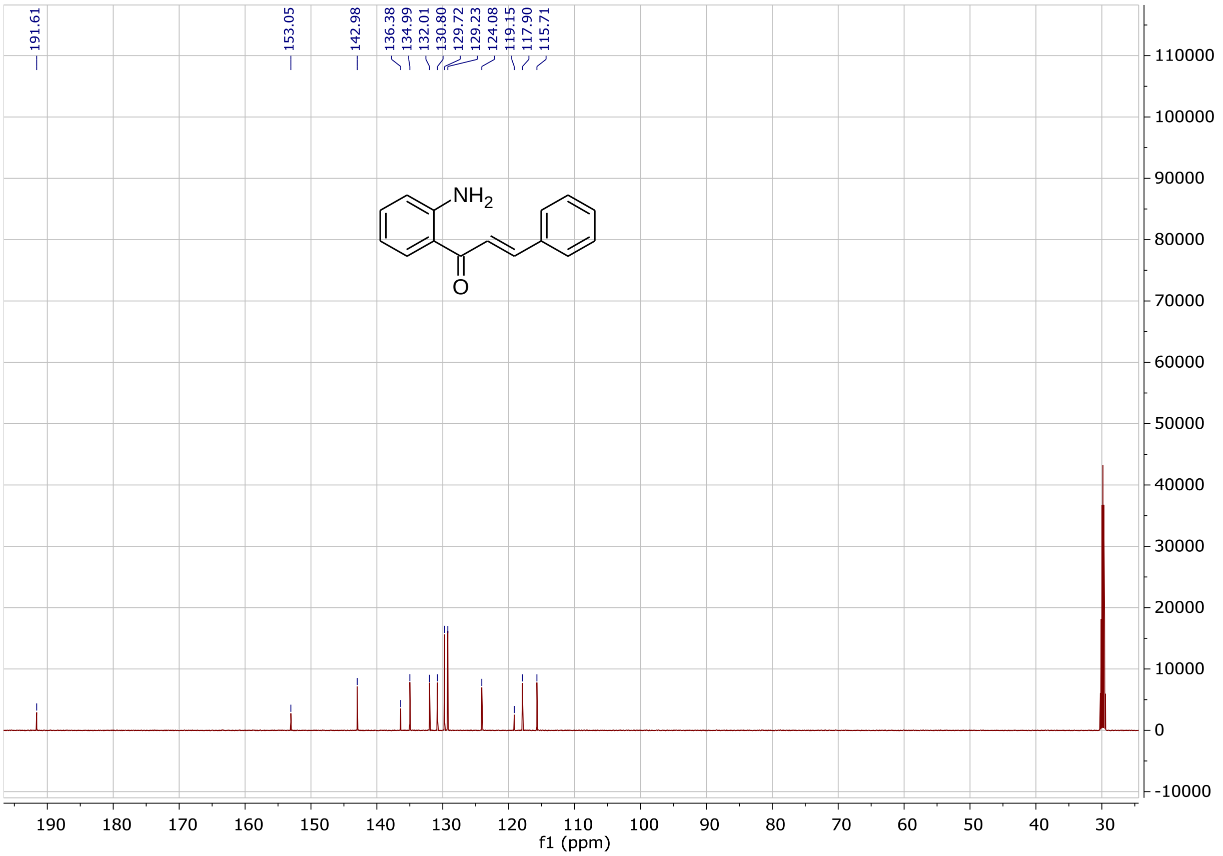

Figure S2. ^{13}C-NMR (150 MHz, acetone-d_6) spectrum of 2′-aminochalcone (**1**)

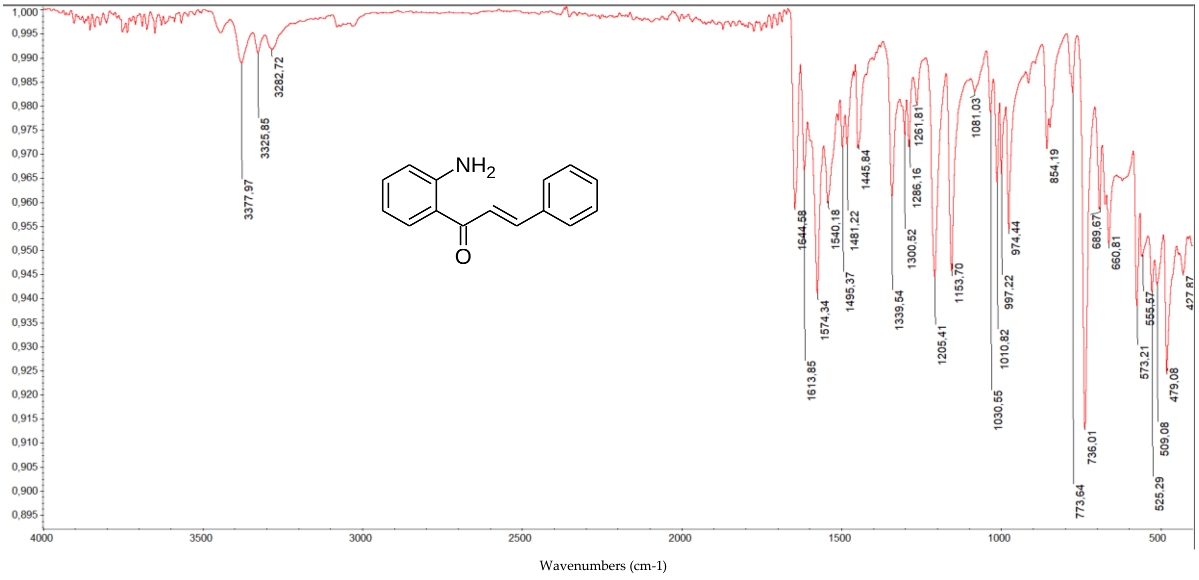

Figure S3. FTIR-ATR spectrum of 2′-aminochalcone (**1**)

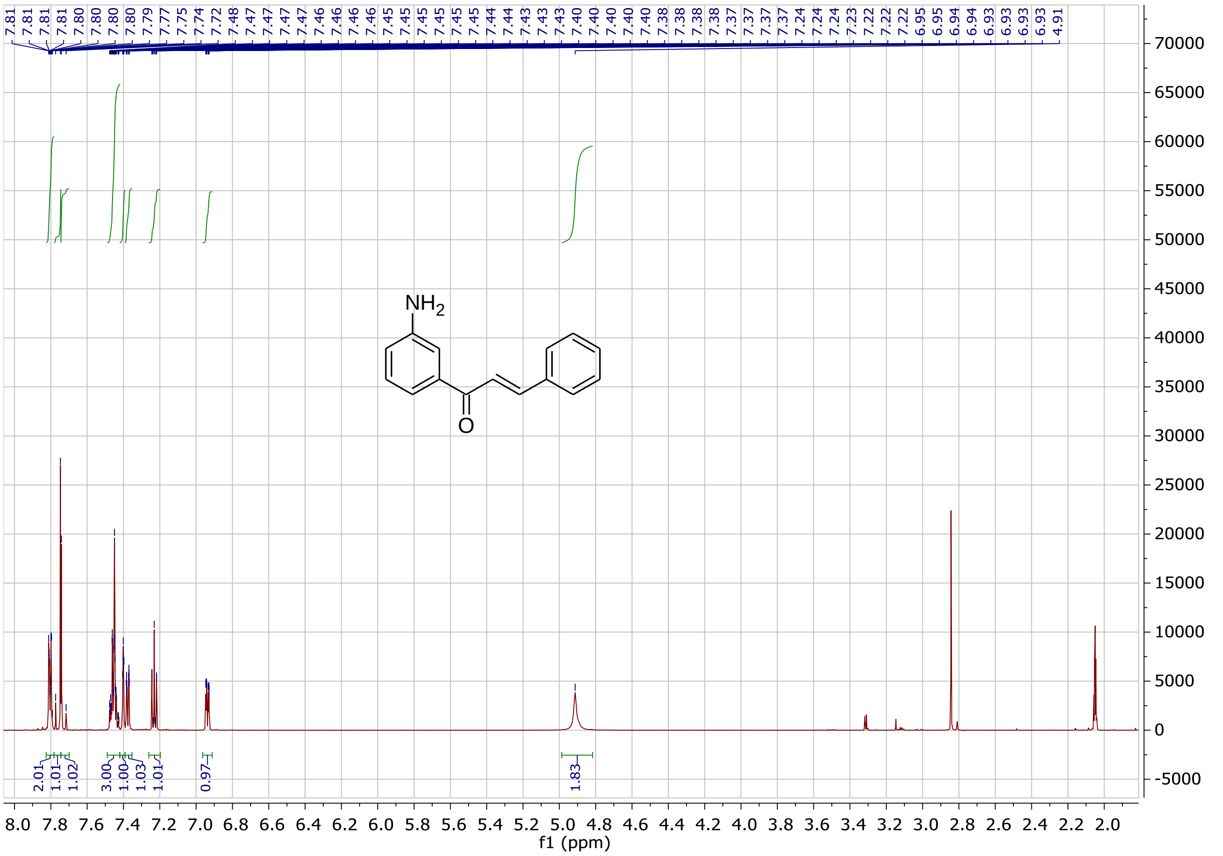

Figure S4. ^{1}H-NMR (600 MHz, acetone-d_6) spectrum of 3′-aminochalcone (**2**)

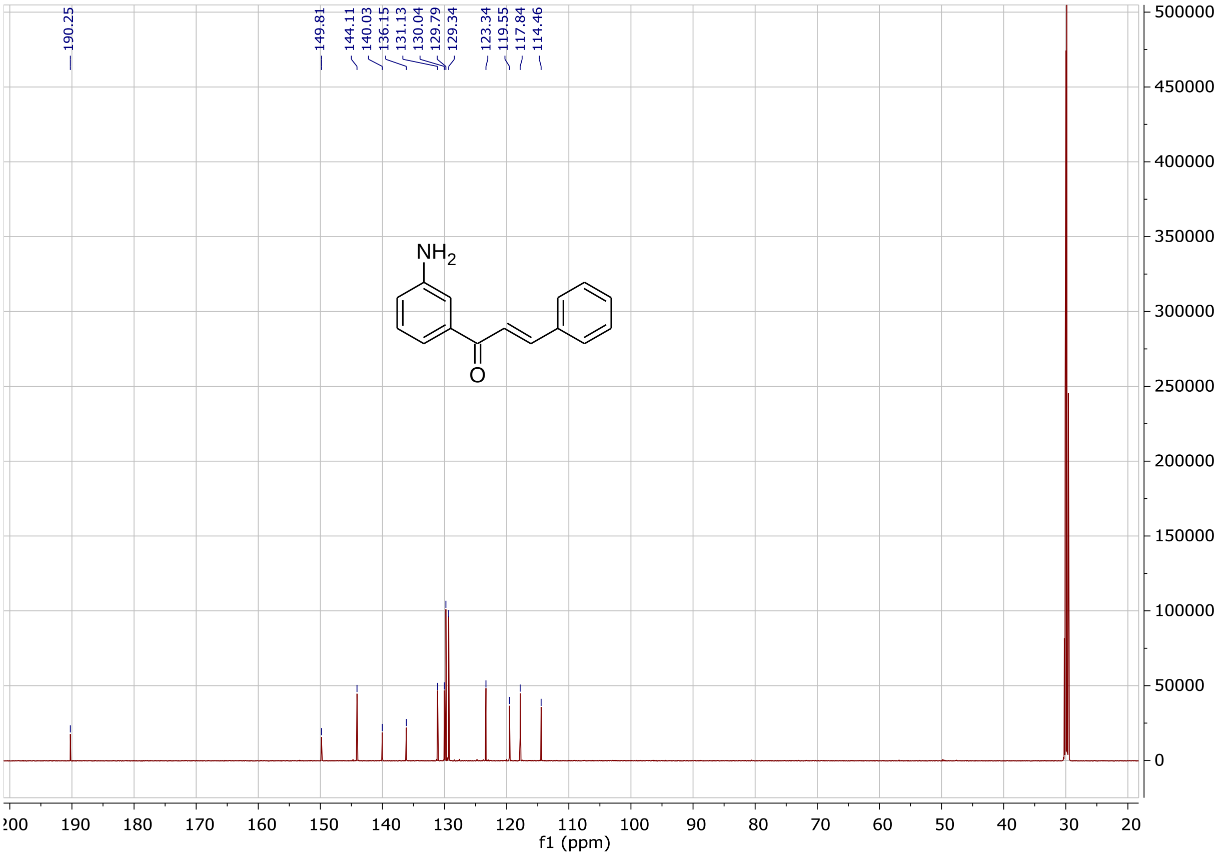

Figure S5. ^{13}C-NMR (150 MHz, acetone-d_6) spectrum of 3′-aminochalcone (**2**)

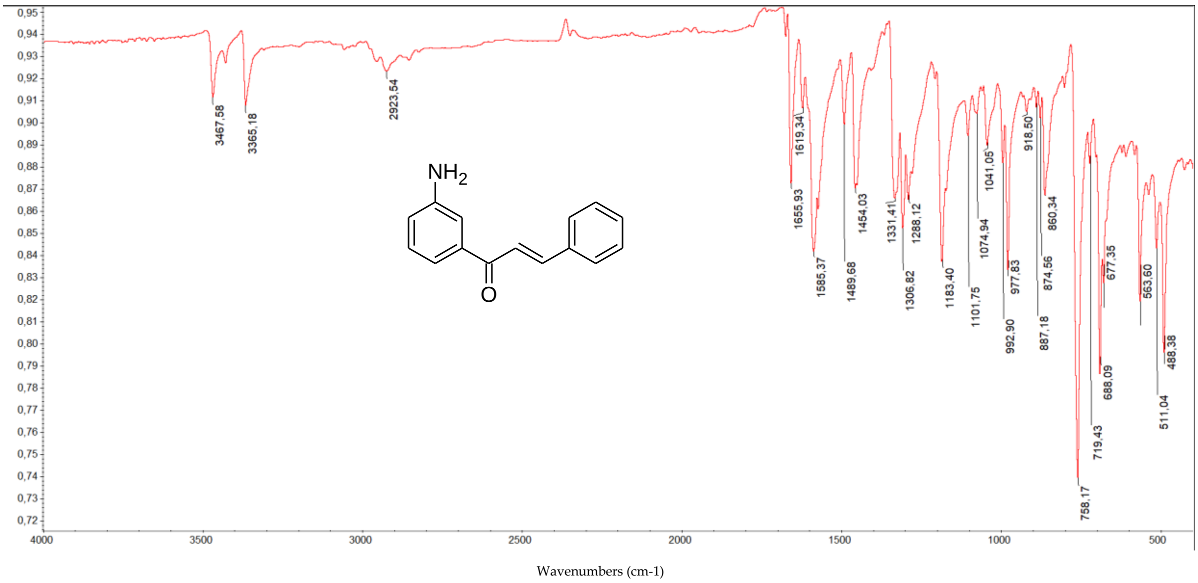

Figure S6. FTIR-ATR spectrum of 3′-aminochalcone (**2**)

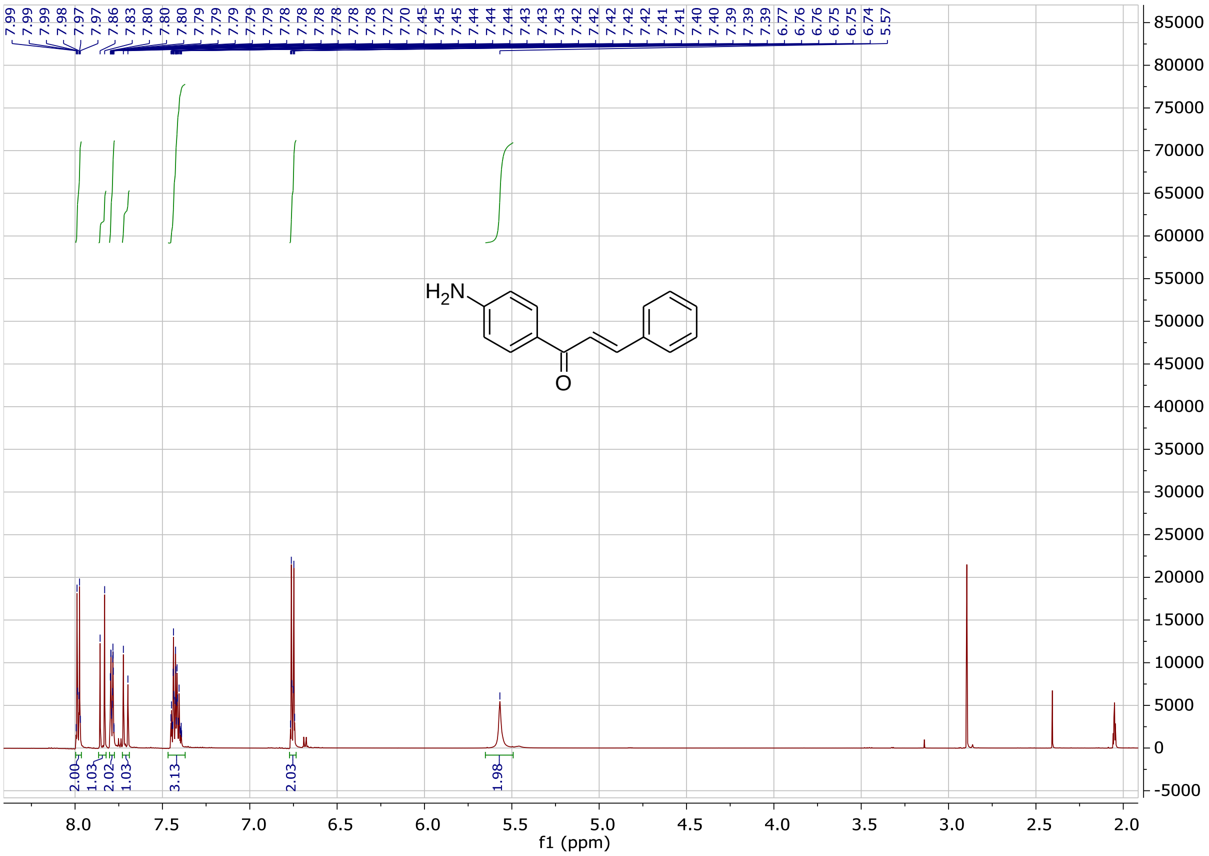

Figure S7. ^{1}H-NMR (600 MHz, acetone-d_6) spectrum of 4′-aminochalcone (**3**)

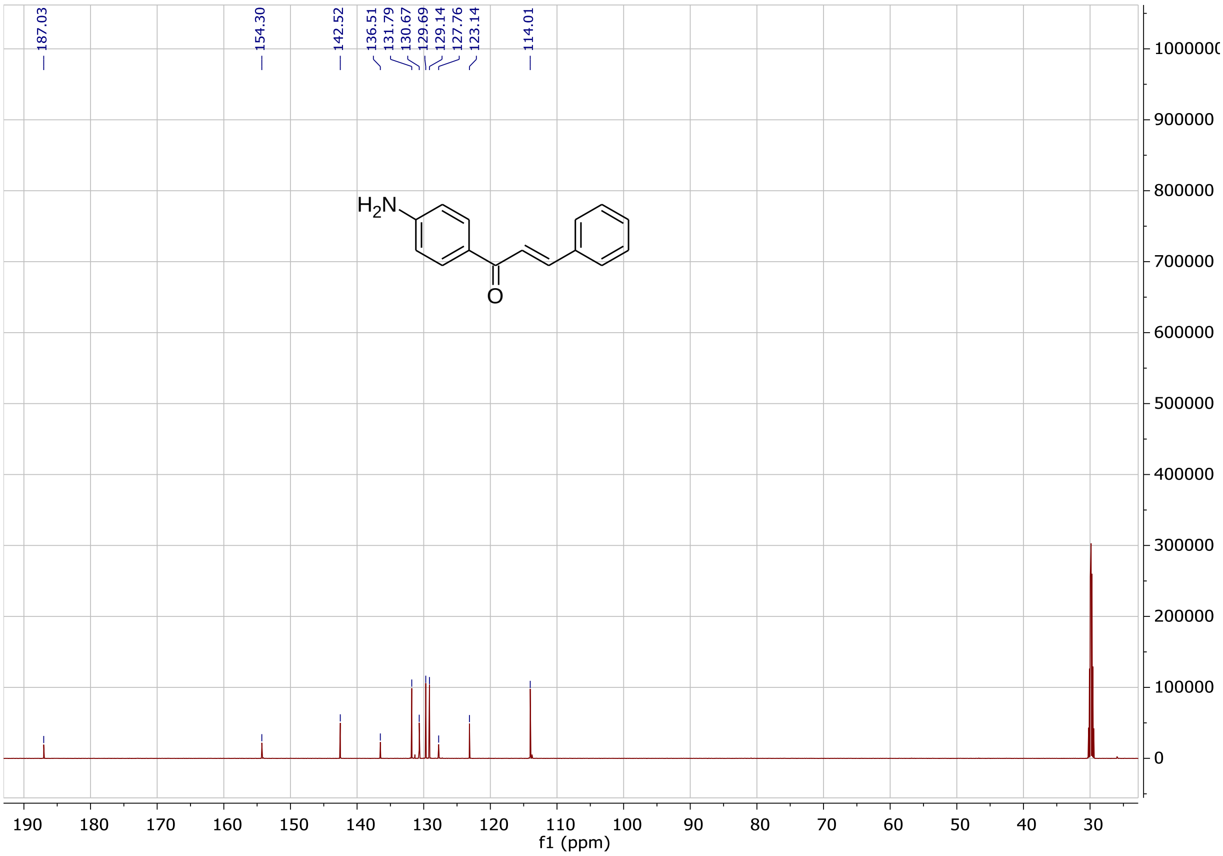

Figure S8. ^{13}C-NMR (150 MHz, acetone-*d*$_6$) spectrum of 4′-aminochalcone (**3**)

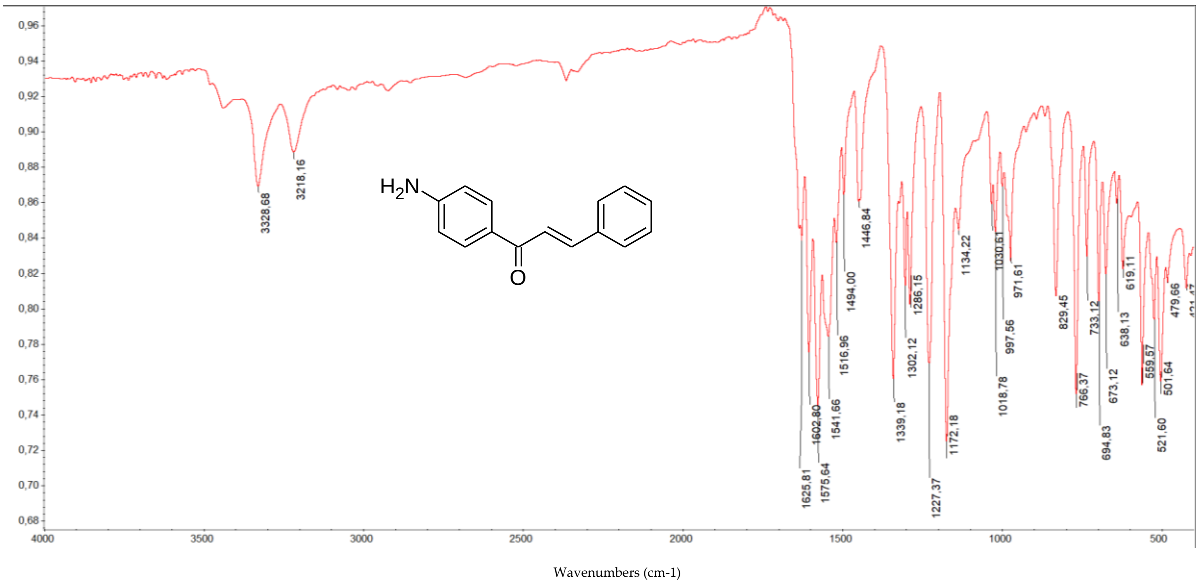

Figure S9. FTIR-ATR spectrum of 4′-aminochalcone (**3**)

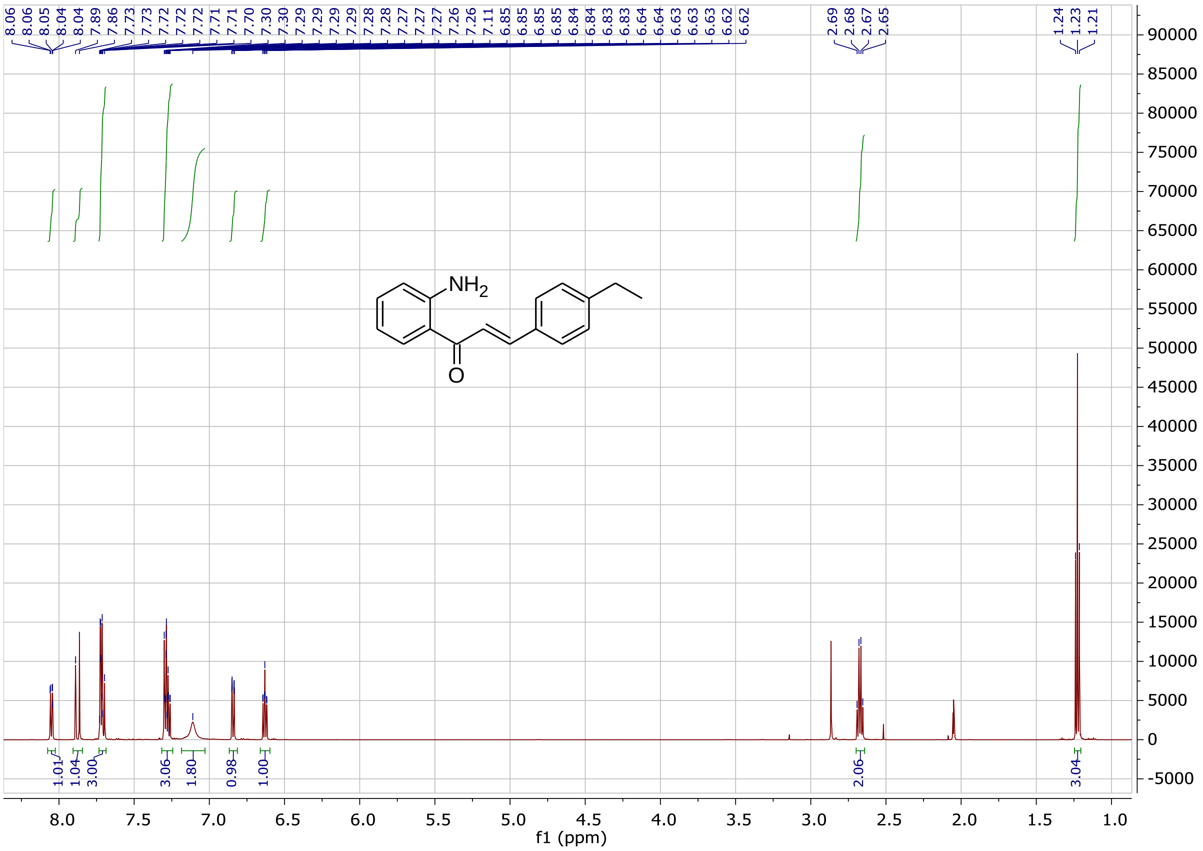

Figure S10. ^{1}H-NMR (600 MHz, acetone-d_6) spectrum of 2′-amino-4-ethylchalcone (**4**)

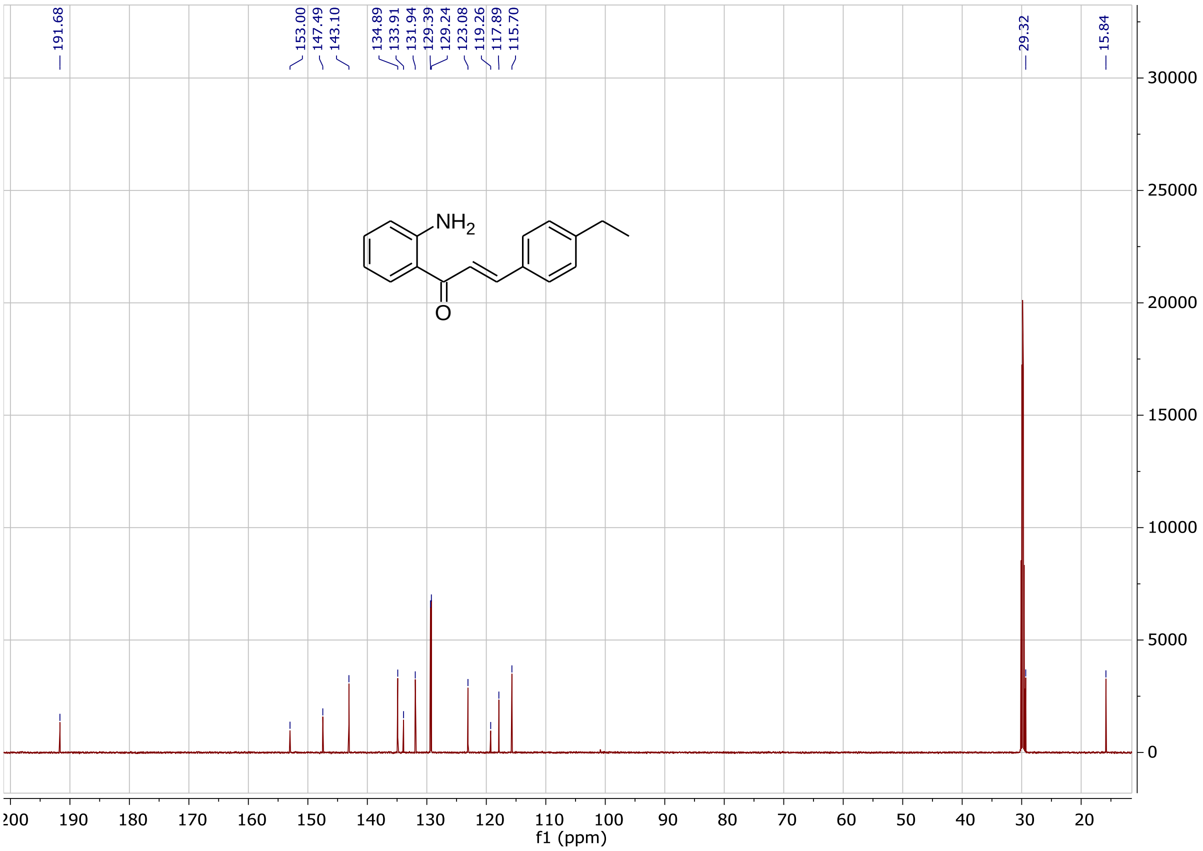

Figure S11. ^{13}C-NMR (150 MHz, acetone-d_6) spectrum of 2′-amino-4-ethylchalcone (**4**)

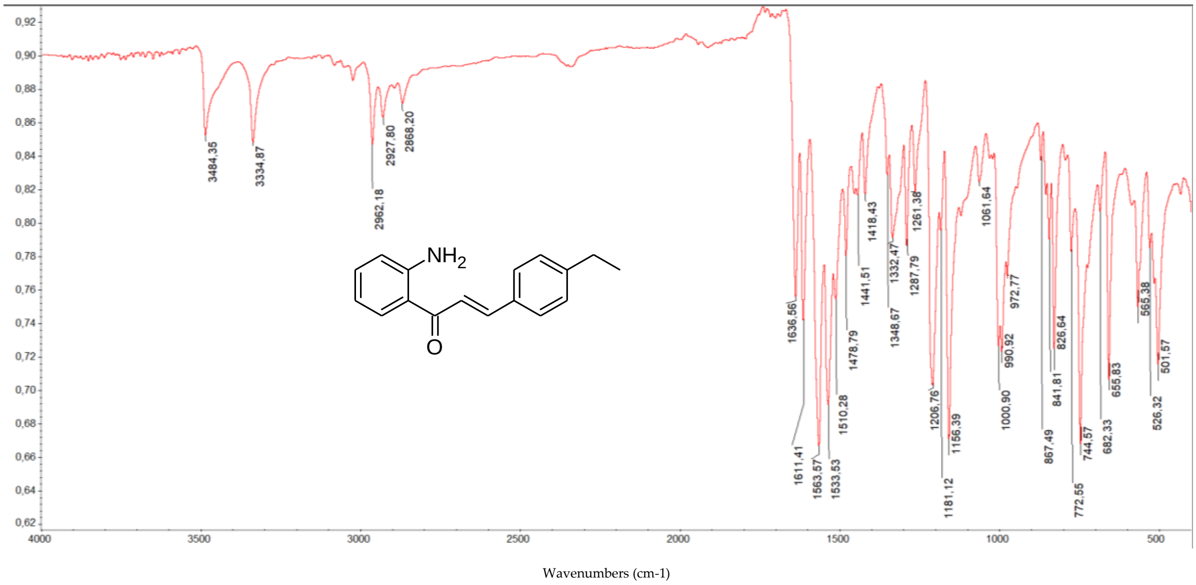

Figure S12. FTIR-ATR spectrum of 2′-amino-4-ethylchalcone (**4**)

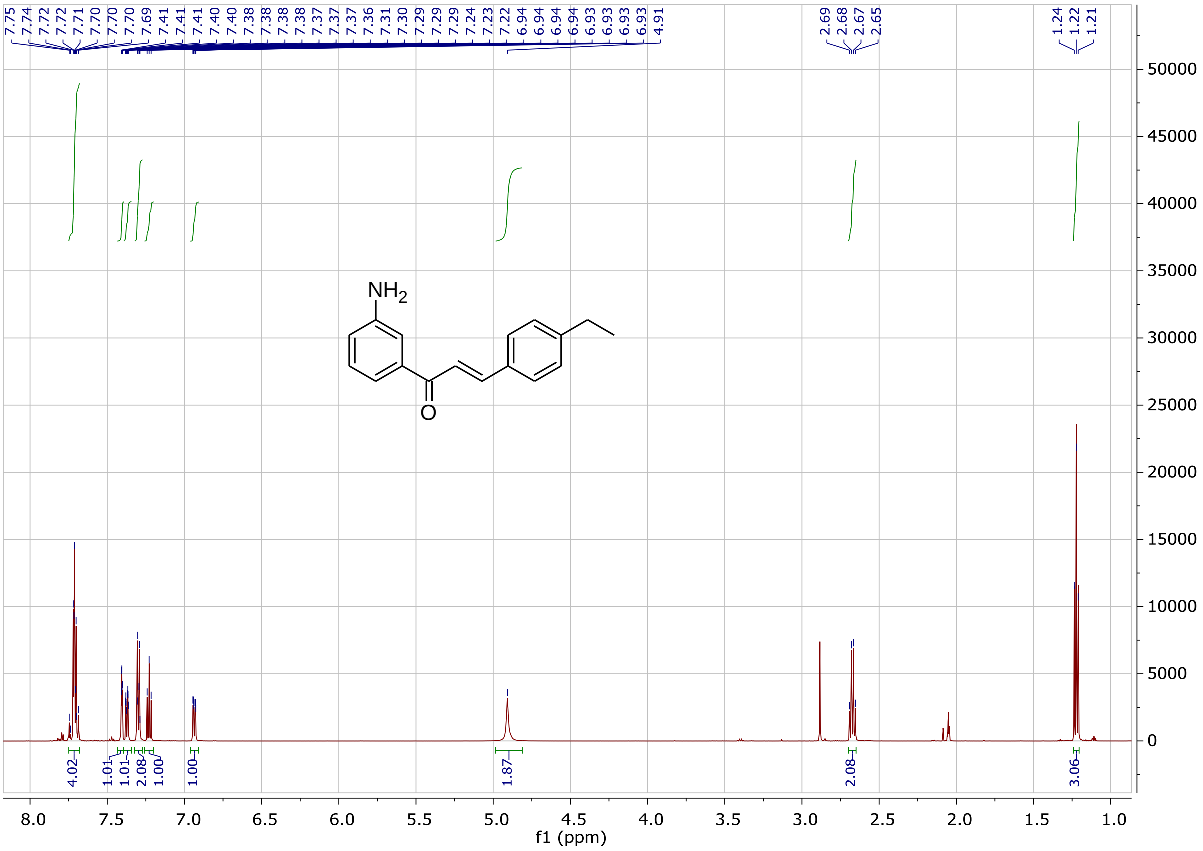

Figure S13. ¹H-NMR (600 MHz, acetone-d_6) spectrum of 3′-amino-4-ethylchalcone (**5**)

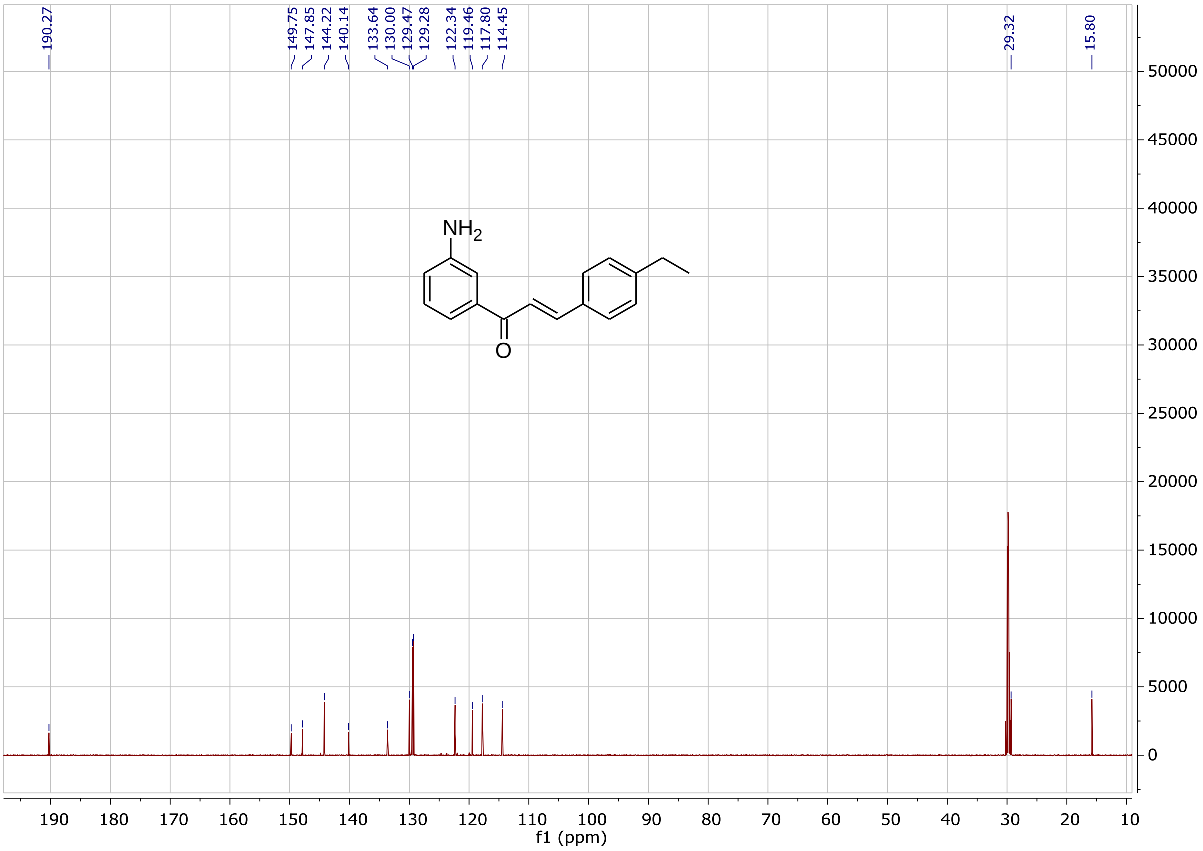

Figure S14. ^{13}C-NMR (150 MHz, acetone-d_6) spectrum of 3′-amino-4-ethylchalcone (**5**)

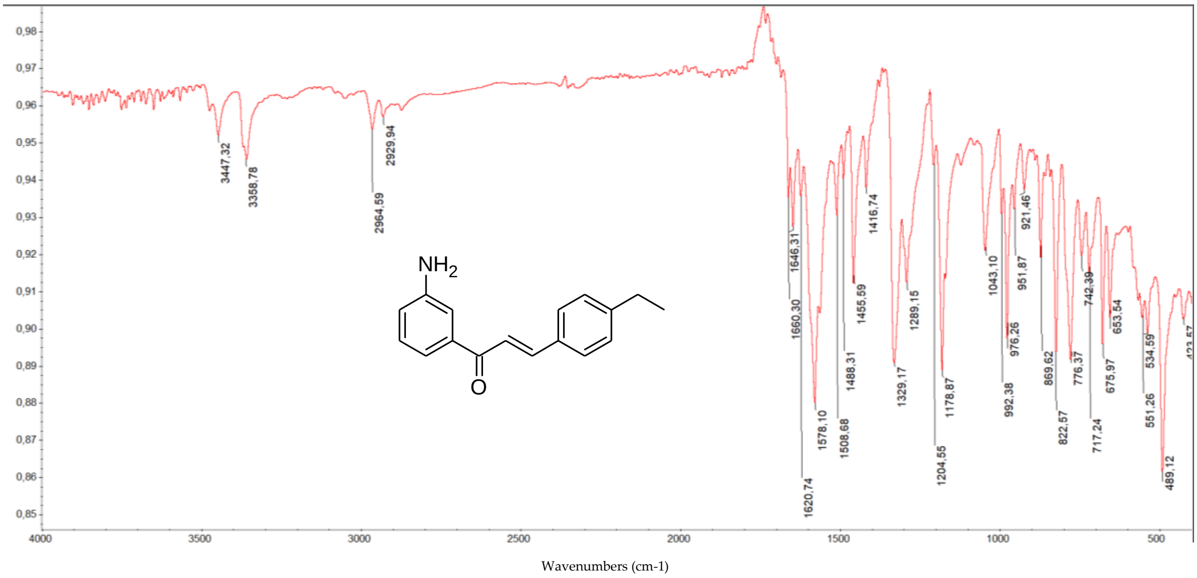

Figure S15. FTIR-ATR spectrum of 3′-amino-4-ethylchalcone (**5**)

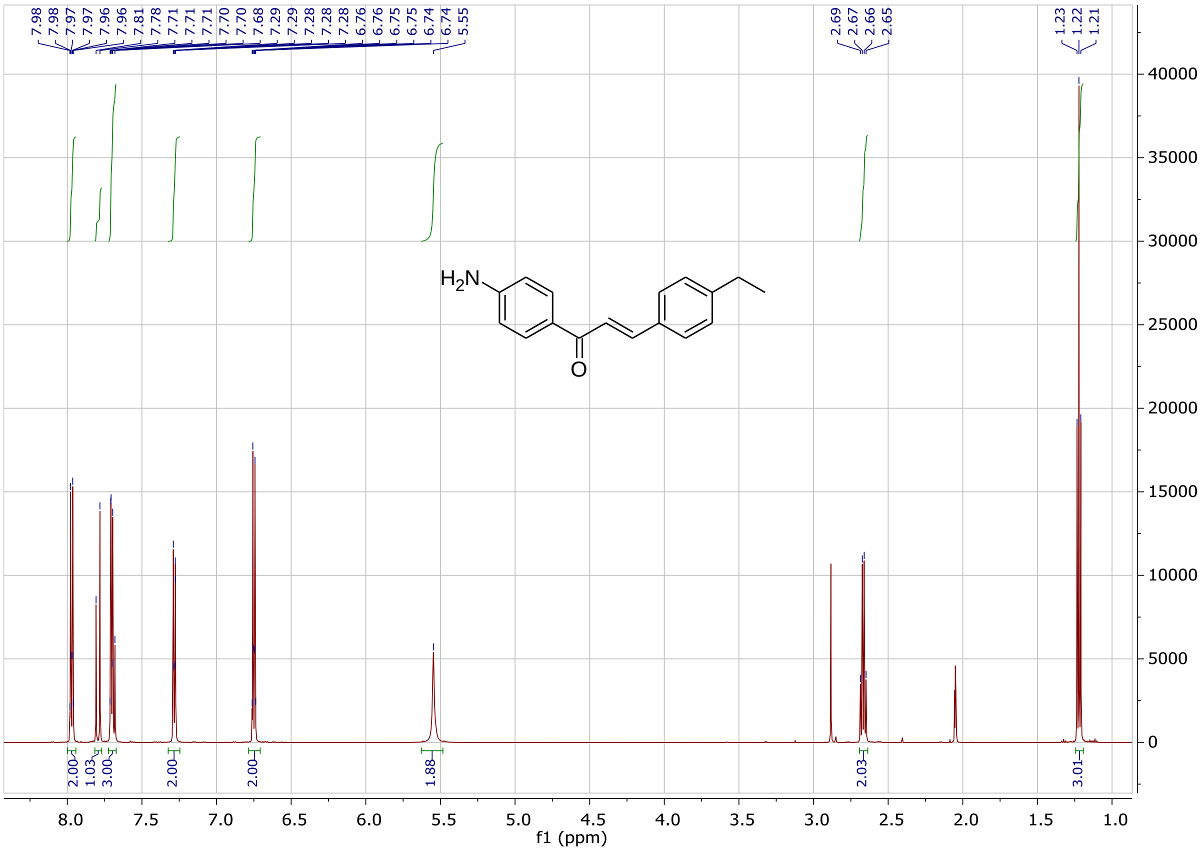

Figure S16. ^{1}H-NMR (600 MHz, acetone-*d*6) spectrum of 4′-amino-4-ethylchalcone (**6**)

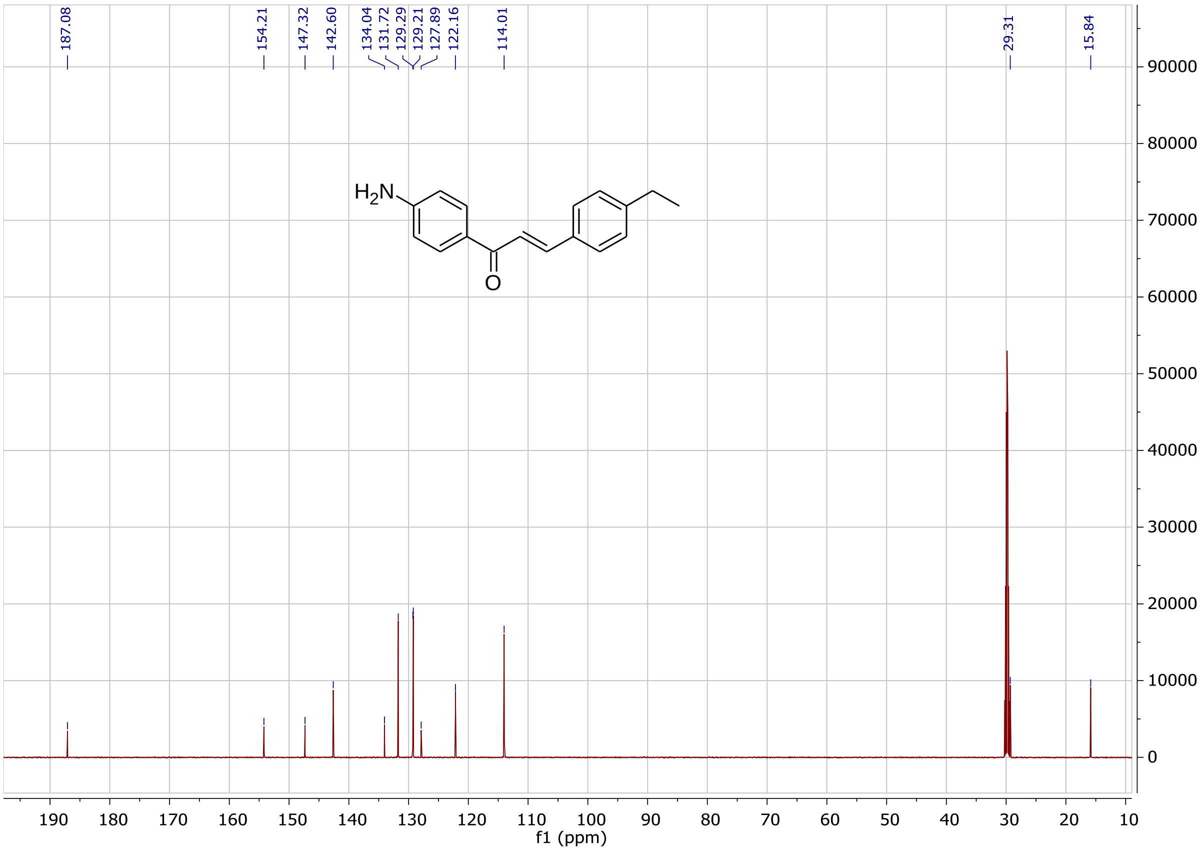

Figure S17. ^{13}C-NMR (150 MHz, acetone-*d*6) spectrum of 4′-amino-4-ethylchalcone (**6**)

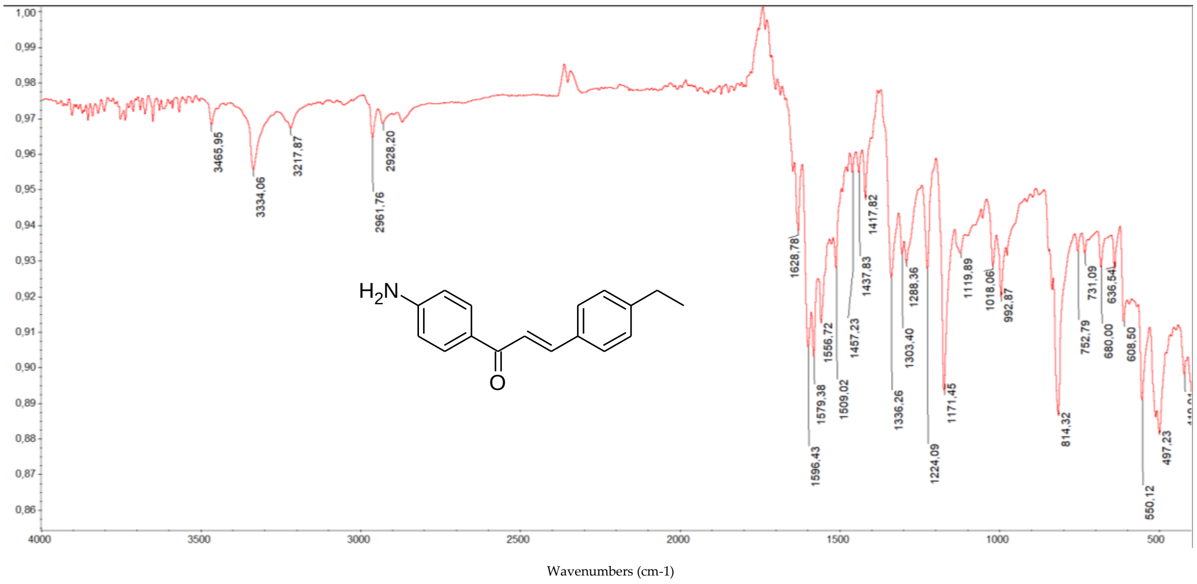

Figure S18. FTIR-ATR spectrum of 4′-amino-4-ethylchalcone (**6**)

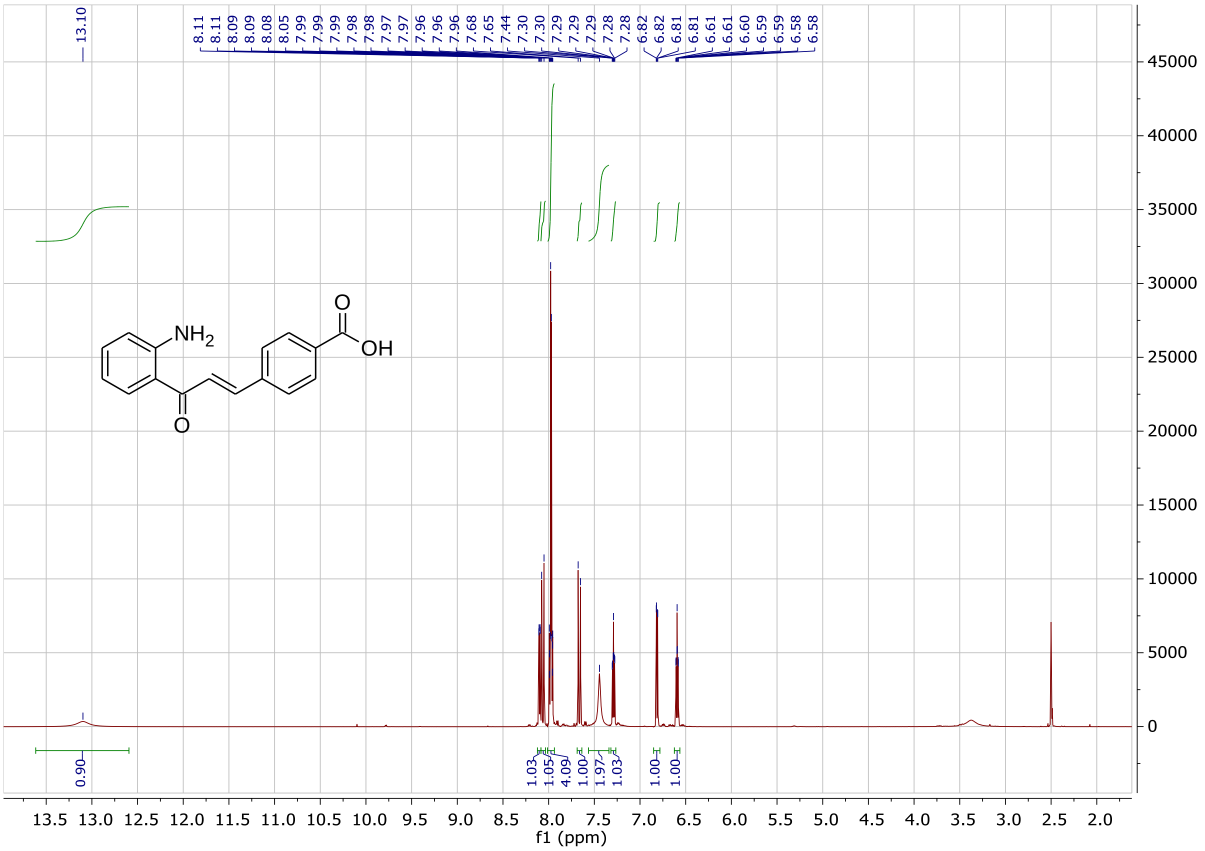

Figure S19. ^{1}H-NMR (600 MHz, DMSO-d_6) spectrum of 2′-amino-4-carboxychalcone (**7**)

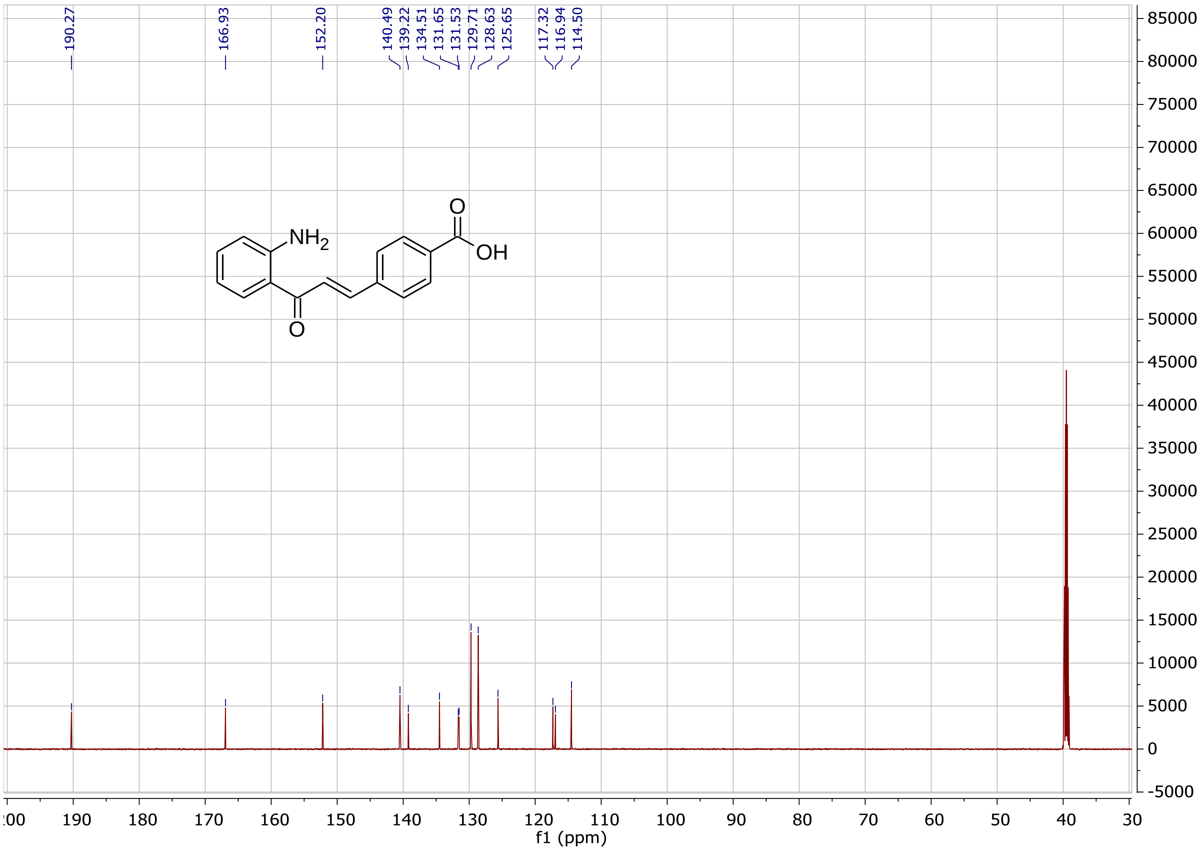

Figure S20. ^{13}C-NMR (150 MHz, DMSO-d_6) spectrum of 2′-amino-4-carboxychalcone (**7**)

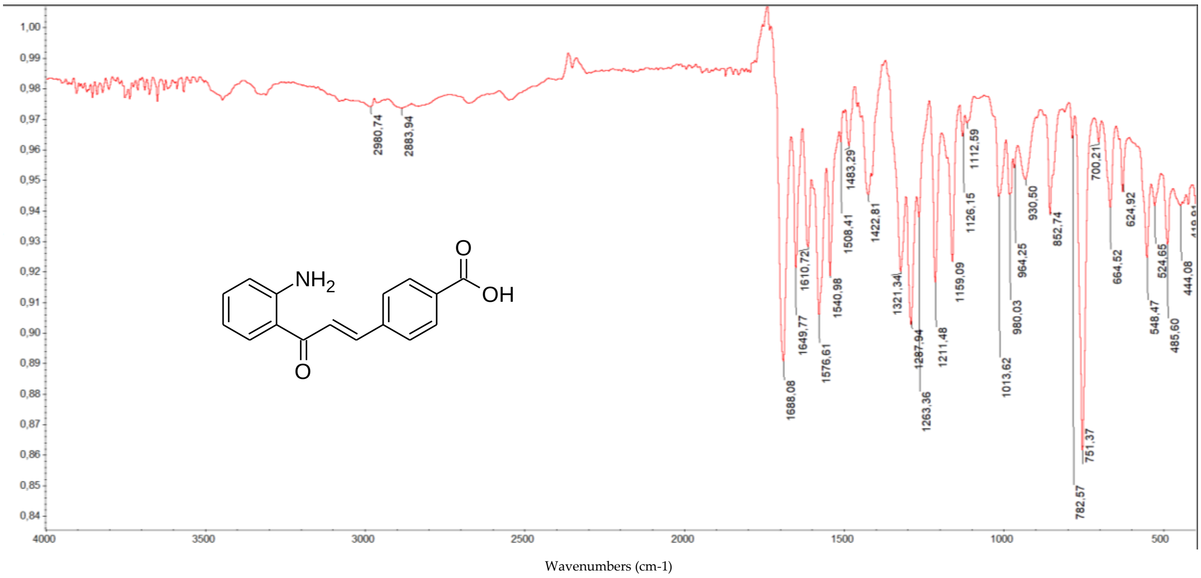

Figure S21. FTIR-ATR spectrum of 2′-amino-4-carboxychalcone (**7**)

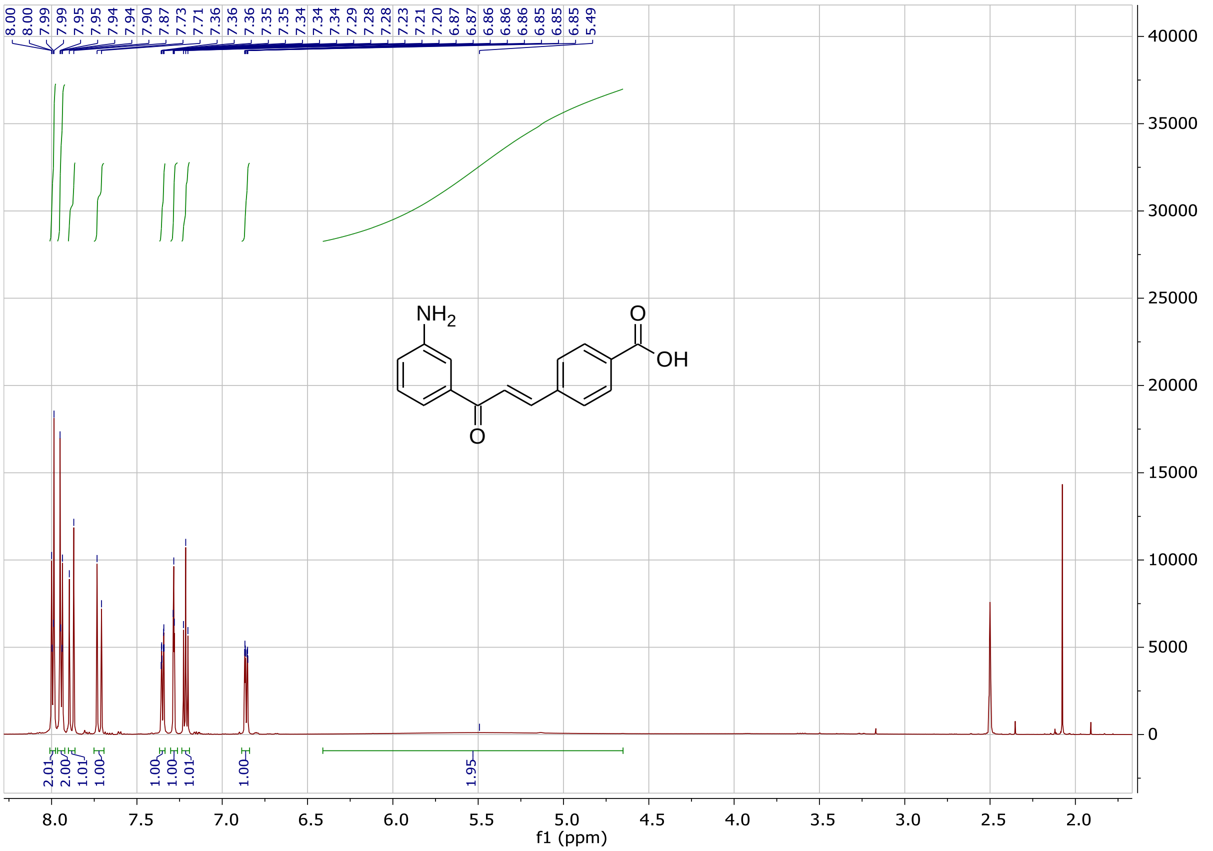

Figure S22. ^{1}H-NMR (600 MHz, DMSO-d_6) spectrum of 3′-amino-4-carboxychalcone (**8**)

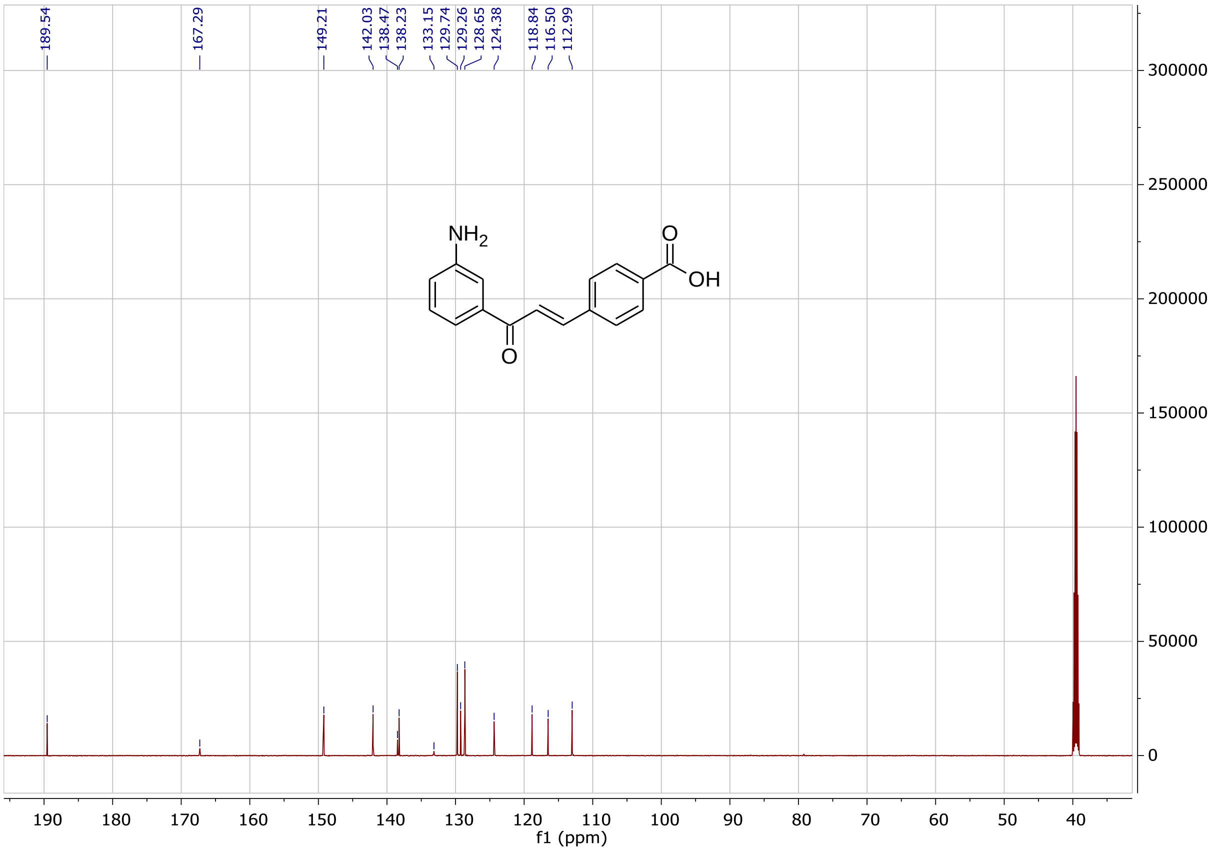

Figure S23. ^{13}C-NMR (150 MHz, DMSO-d_6) spectrum of 3′-amino-4-carboxychalcone (**8**)

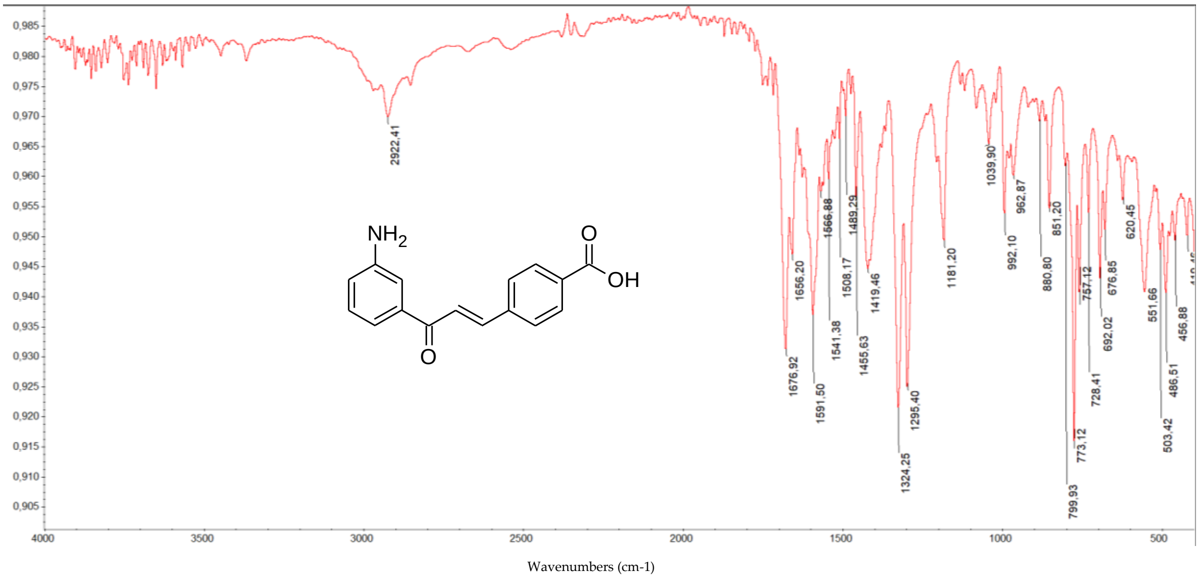

Figure S24. FTIR-ATR spectrum of 3′-amino-4-carboxychalcone (**8**)

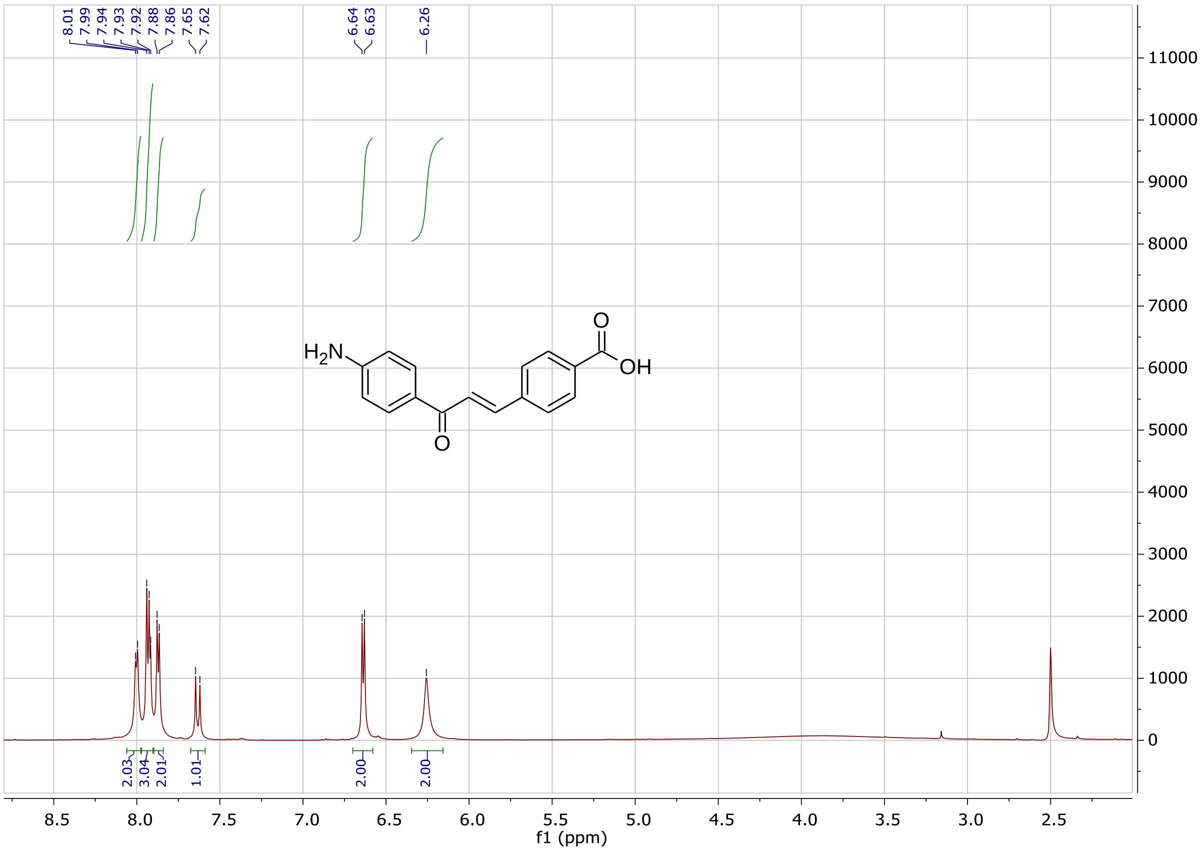

Figure S25. ^{1}H-NMR (600 MHz, DMSO-d_6) spectrum of 4′-amino-4-carboxychalcone (**9**)

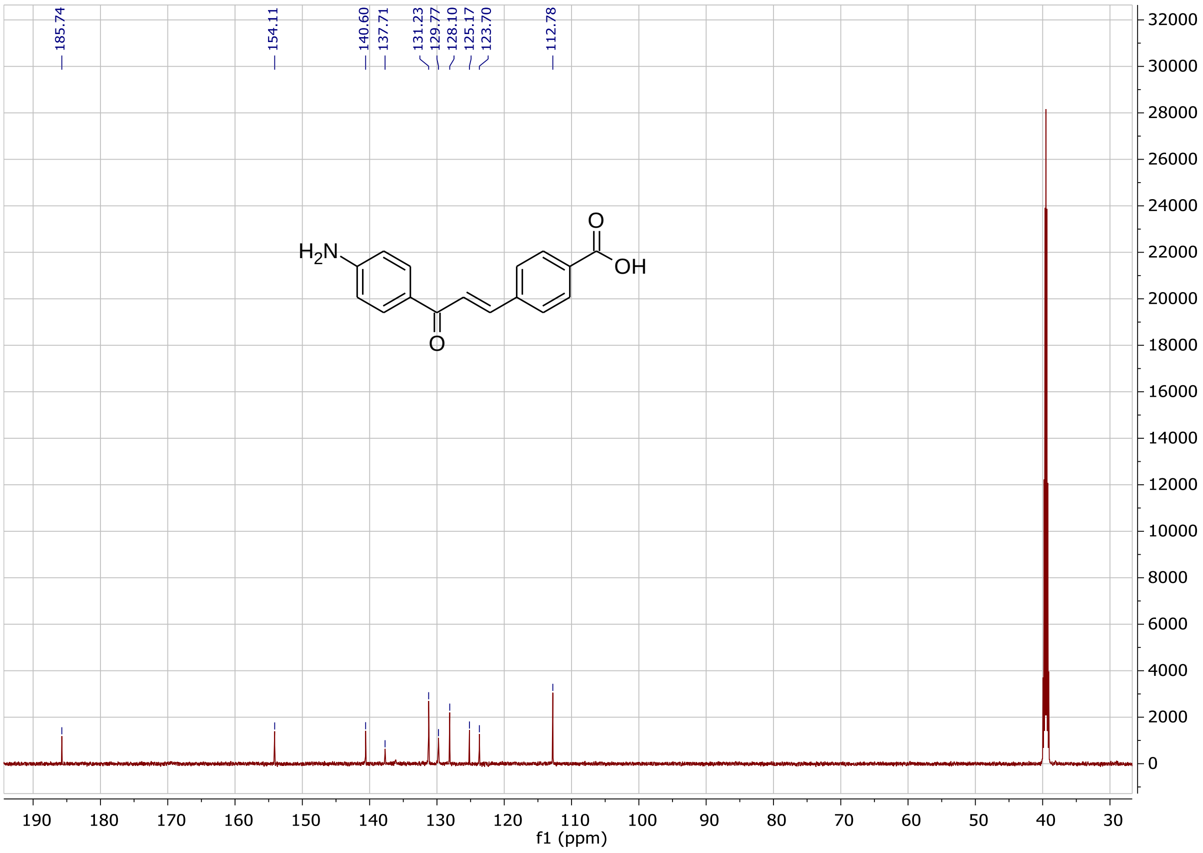

Figure S26. ^{13}C-NMR (150 MHz, DMSO-d_6) spectrum of 4′-amino-4-carboxychalcone (**9**)

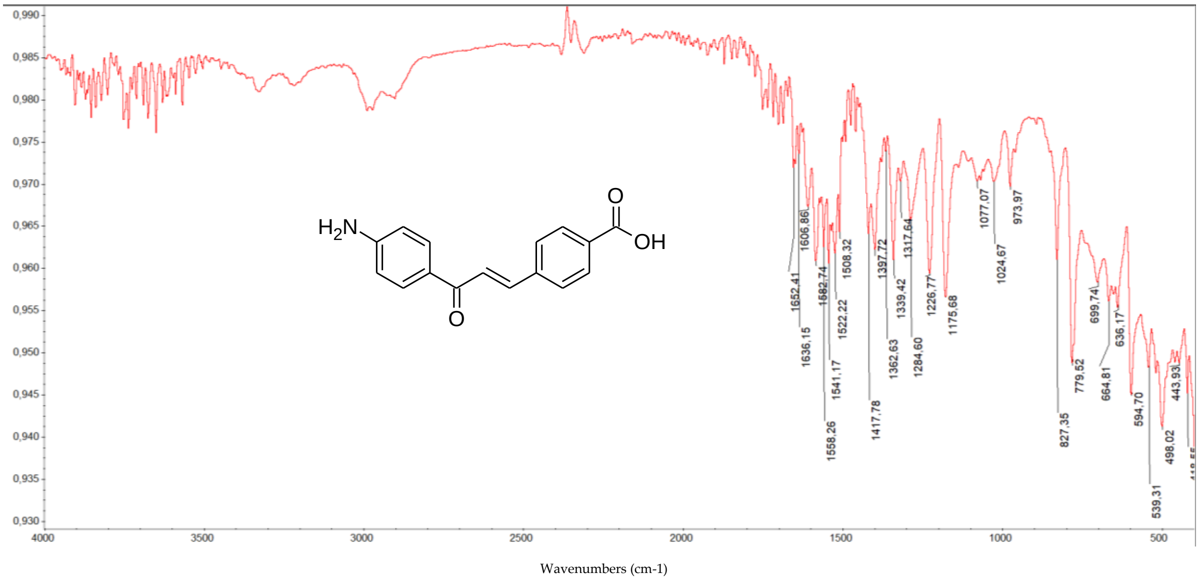

Figure S27. FTIR-ATR spectrum of 4′-amino-4-carboxychalcone (**9**)

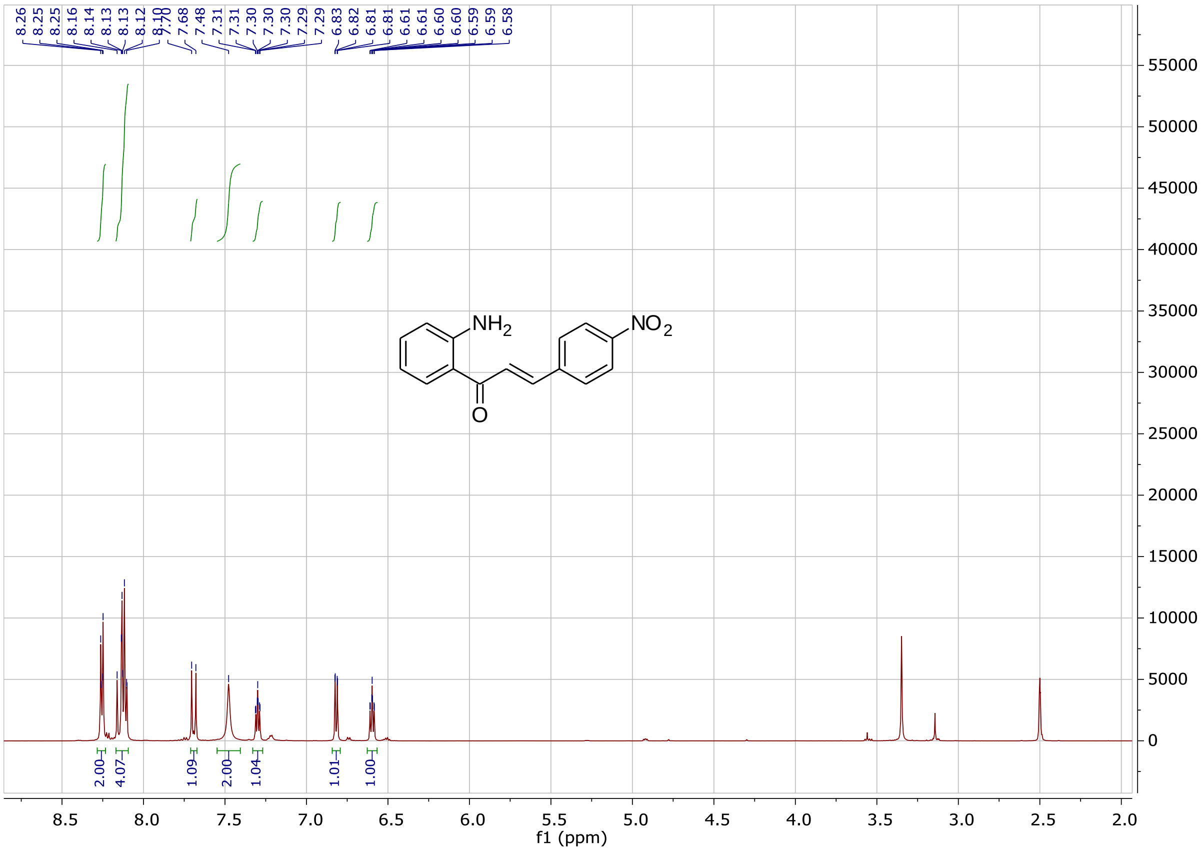

Figure S28. ^{1}H-NMR (600 MHz, DMSO-d_6) spectrum of 2′-amino-4-nitrochalcone (**10**)

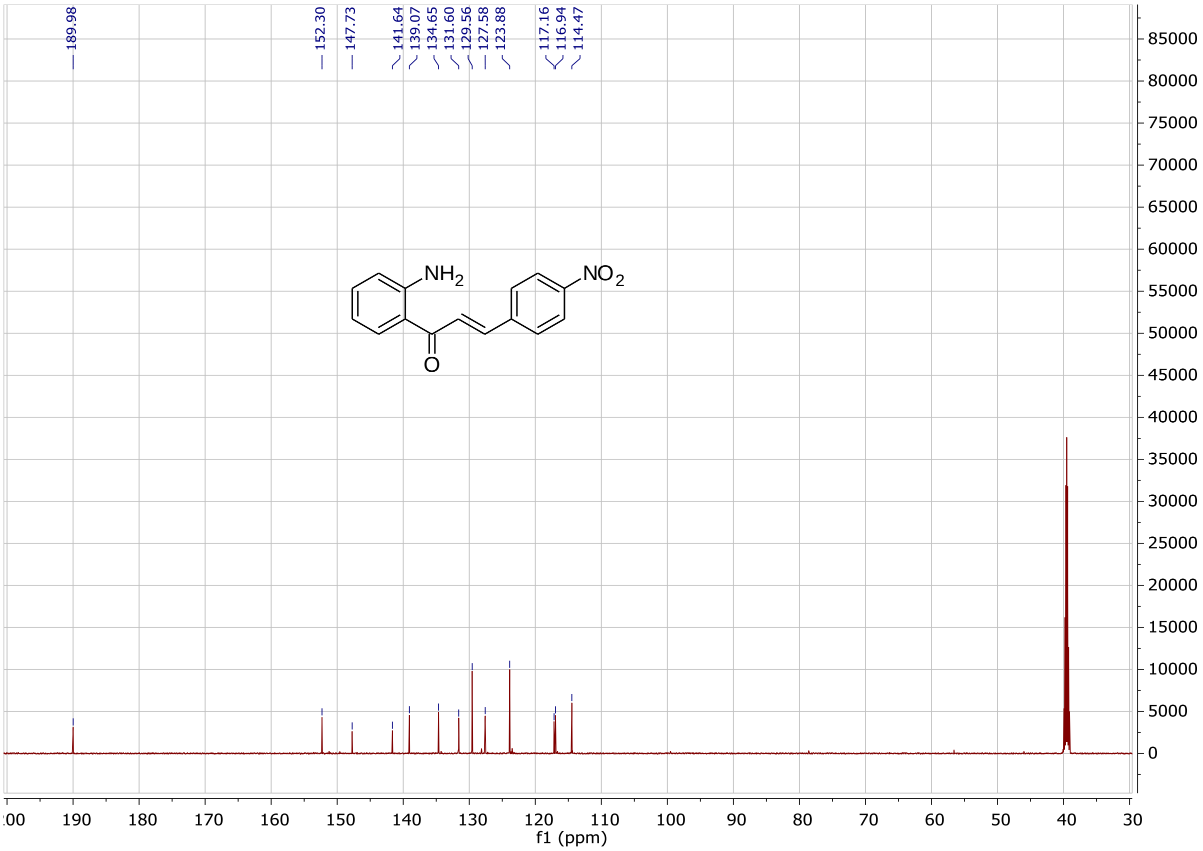

Figure S29. ^{13}C-NMR (150 MHz, DMSO-d_6) spectrum of 2′-amino-4-nitrochalcone (**10**)

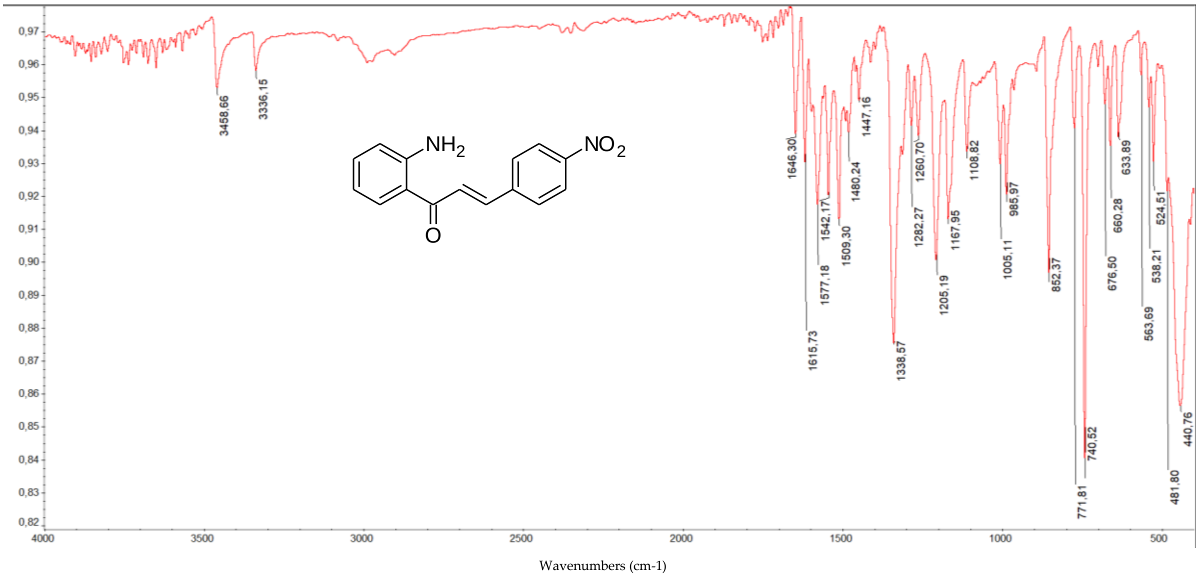

Figure S30. FTIR-ATR spectrum of 2′-amino-4-nitrochalcone (**10**)

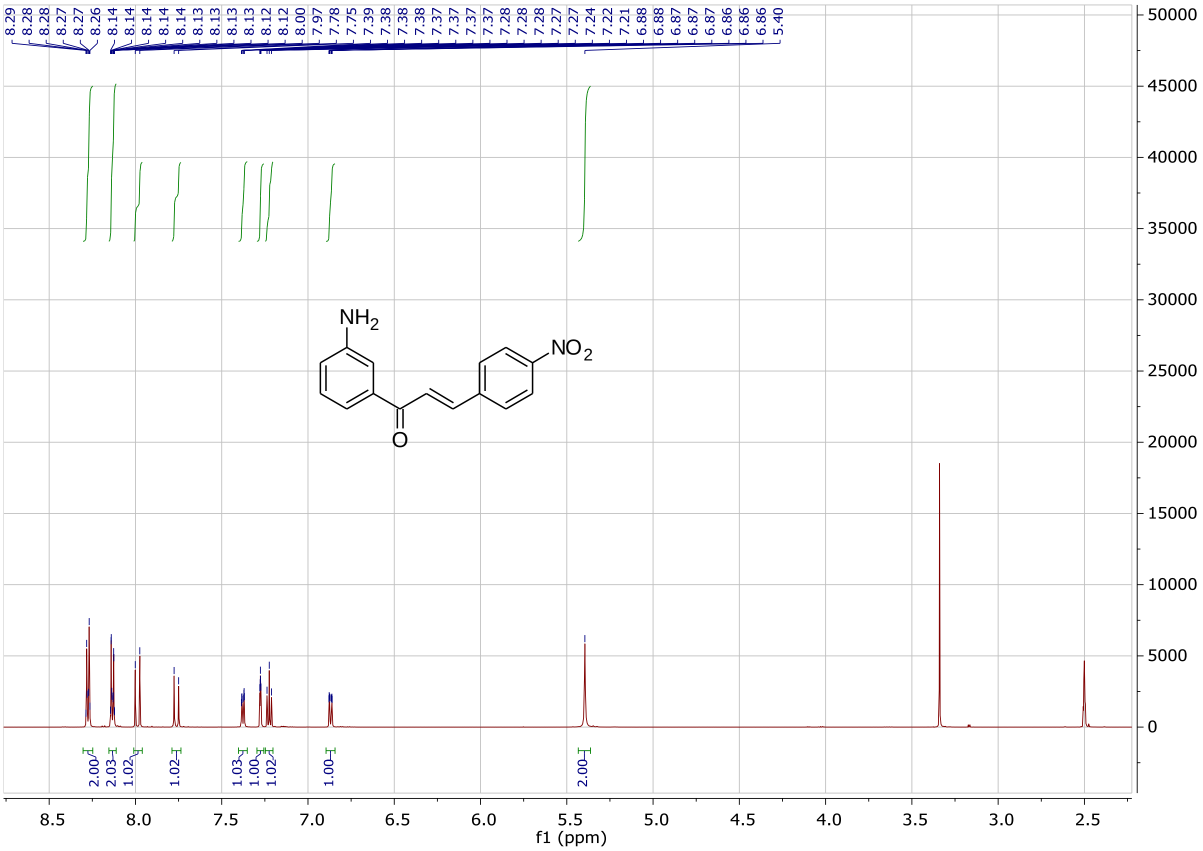

Figure S31. ^{1}H-NMR (600 MHz, DMSO-d_6) spectrum of 3′-amino-4-nitrochalcone (**11**)

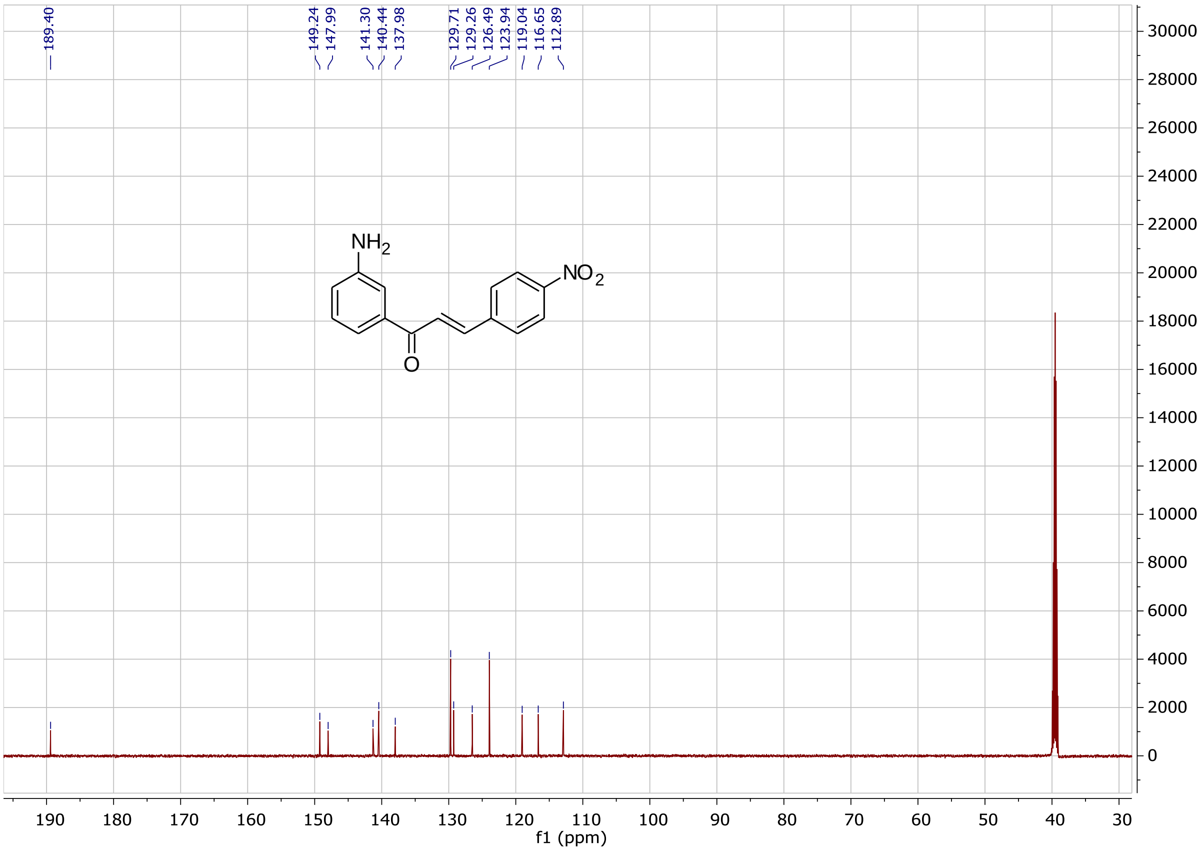

Figure S32. ^{13}C-NMR (150 MHz, DMSO-d_6) spectrum of 3′-amino-4-nitrochalcone (**11**)

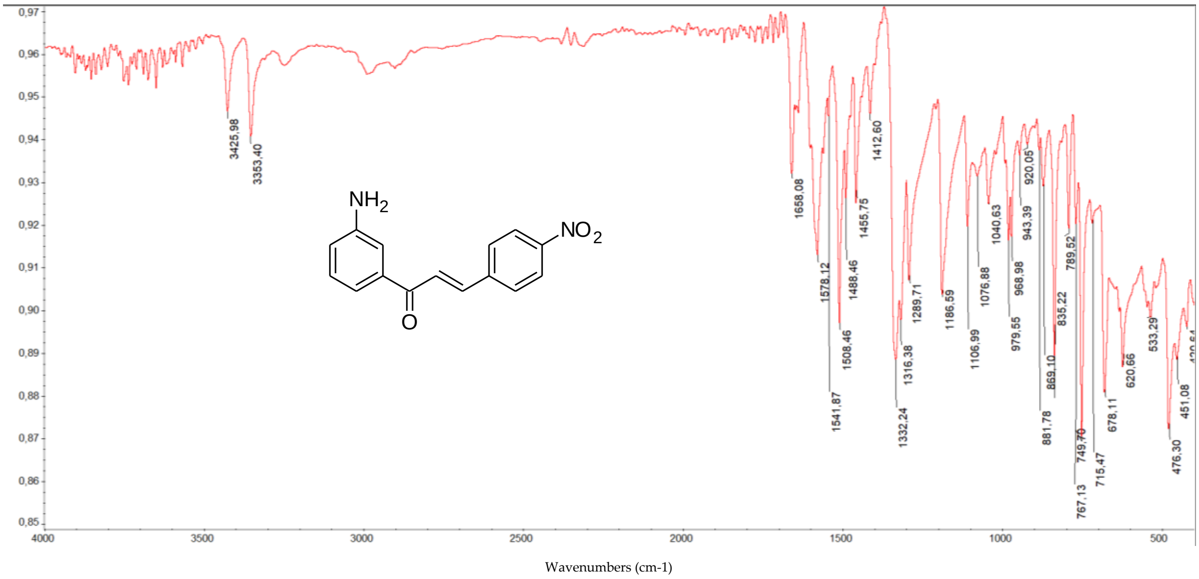

Figure S33. FTIR-ATR spectrum of 3′-amino-4-nitrochalcone (**11**)

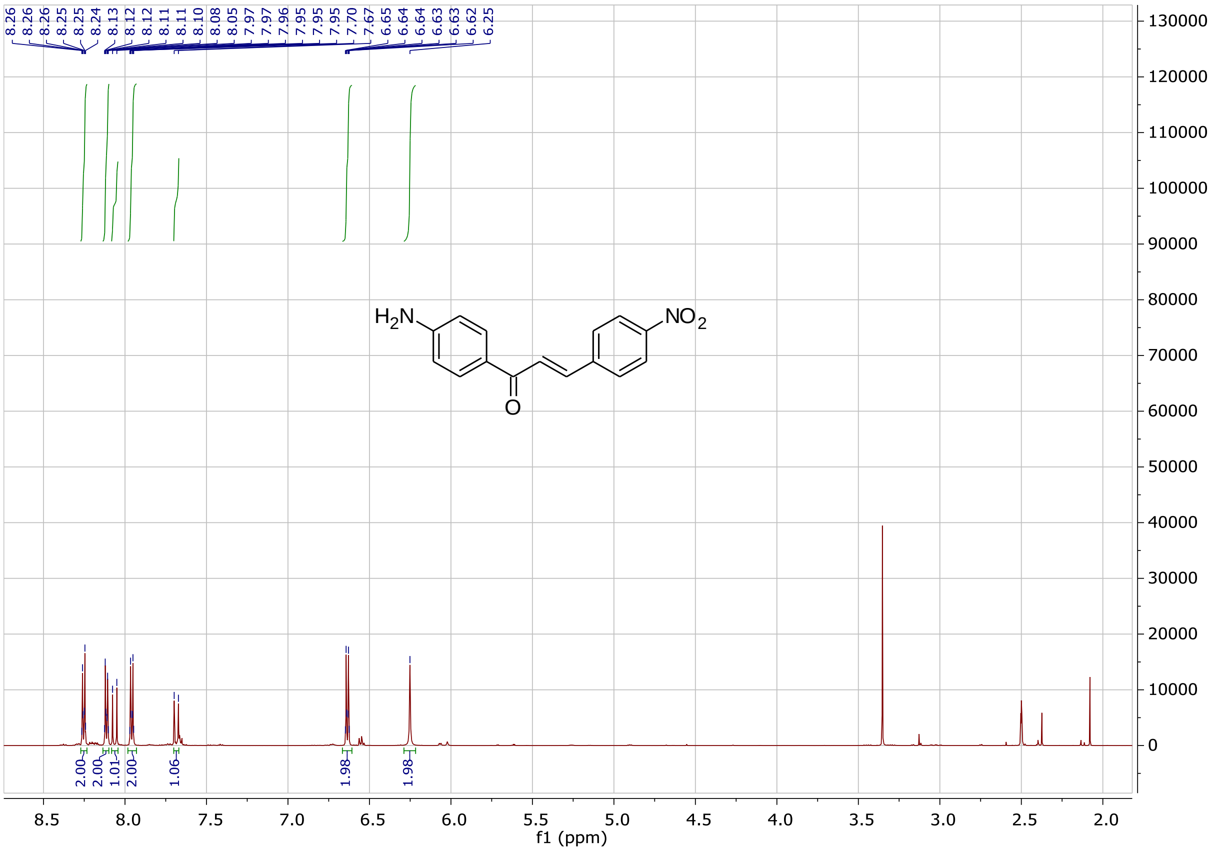

Figure S34. ^{1}H-NMR (600 MHz, DMSO-d_6) spectrum of 4′-amino-4-nitrochalcone (**12**)

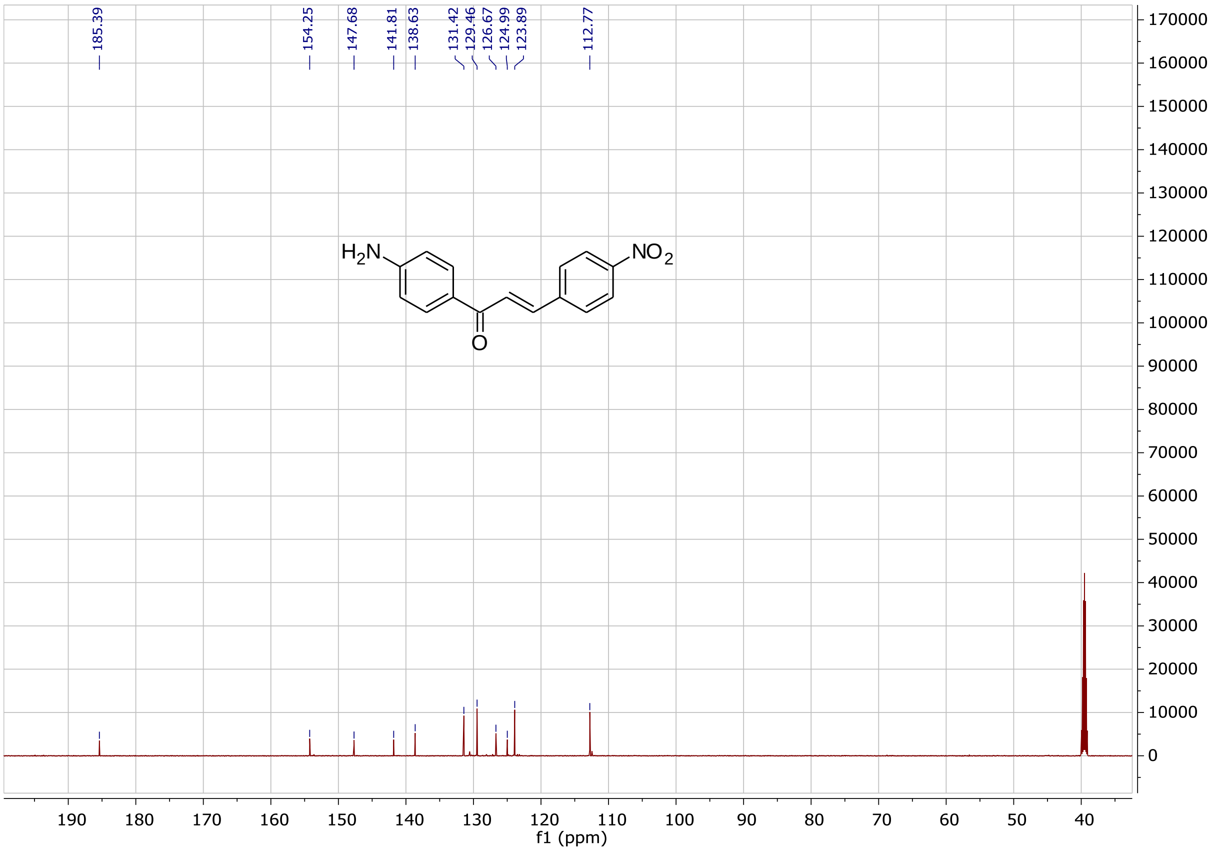

Figure S35. ^{13}C-NMR (150 MHz, DMSO-d_6) spectrum of 4′-amino-4-nitrochalcone (**12**)

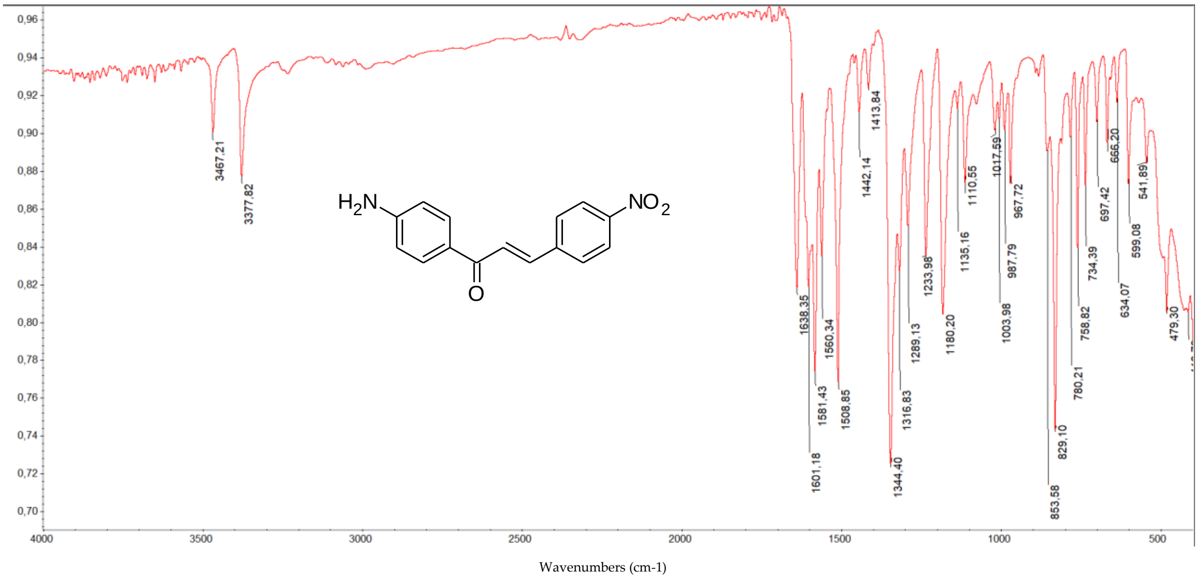

Figure S36. FTIR-ATR spectrum of 4′-amino-4-nitrochalcone (**12**)

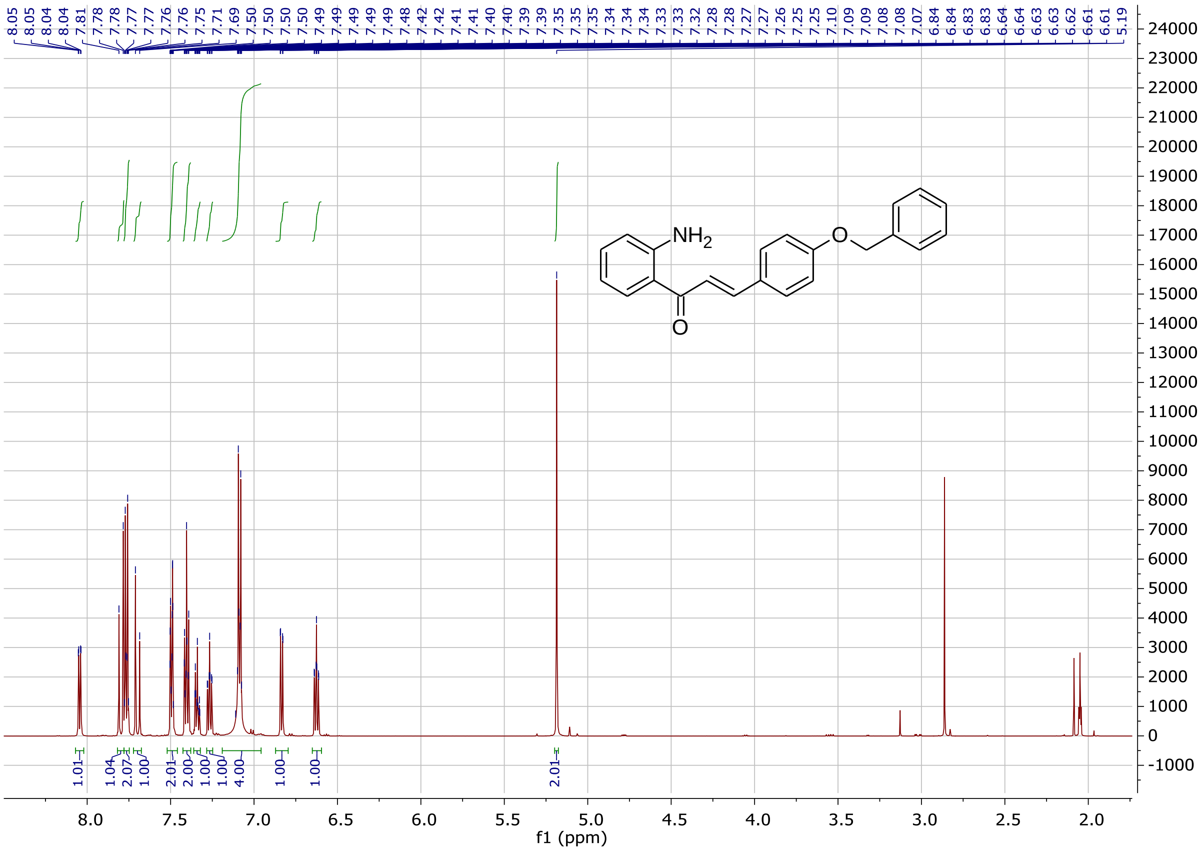

Figure S37. ^{1}H-NMR (600 MHz, acetone-d_6) spectrum of 2′-amino-4-benzyloxychalcone (**13**)

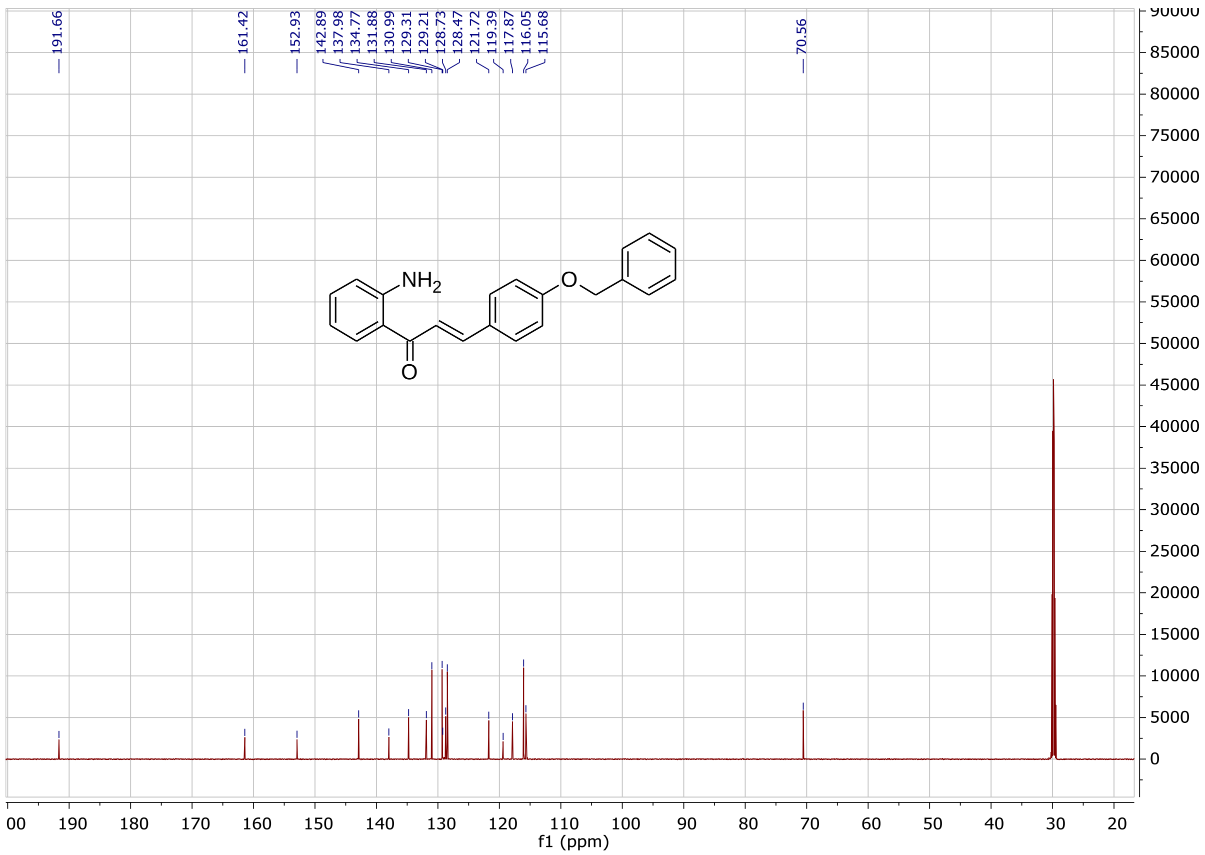

Figure S38. ^{13}C-NMR (150 MHz, acetone-d_6) spectrum of 2′-amino-4-benzyloxychalcone (**13**)

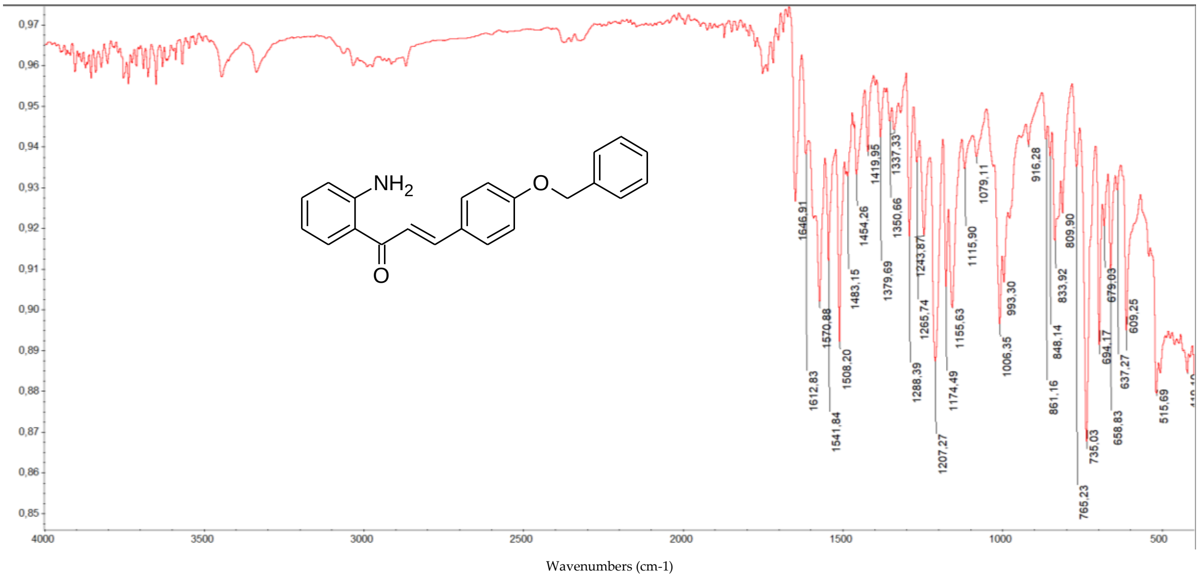

Figure S39. FTIR-ATR spectrum of 2′-amino-4-benzyloxychalcone (**13**)

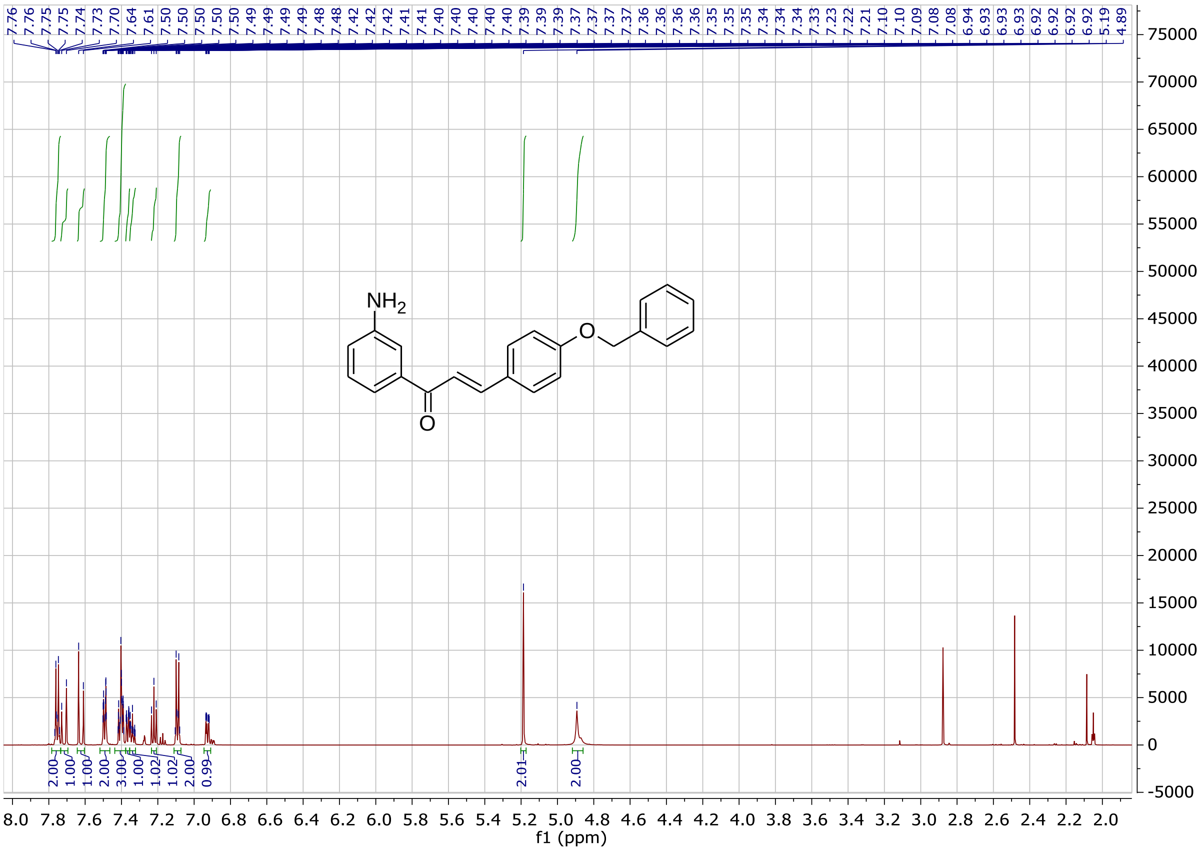

Figure S40. ^{1}H-NMR (600 MHz, acetone-d_6) spectrum of 3′-amino-4-benzyloxychalcone (**14**)

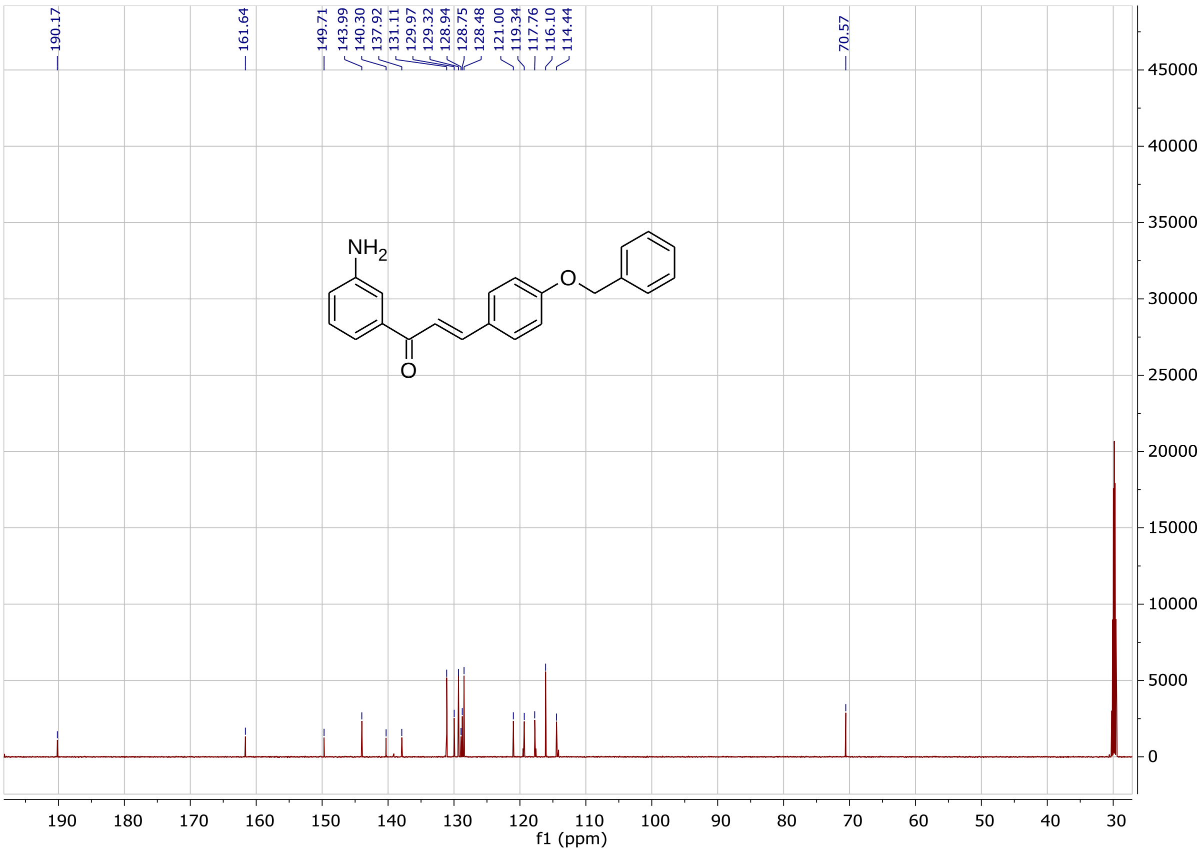

Figure S41. ^{13}C-NMR (150 MHz, acetone-d_6) spectrum of 3′-amino-4-benzyloxychalcone (**14**)

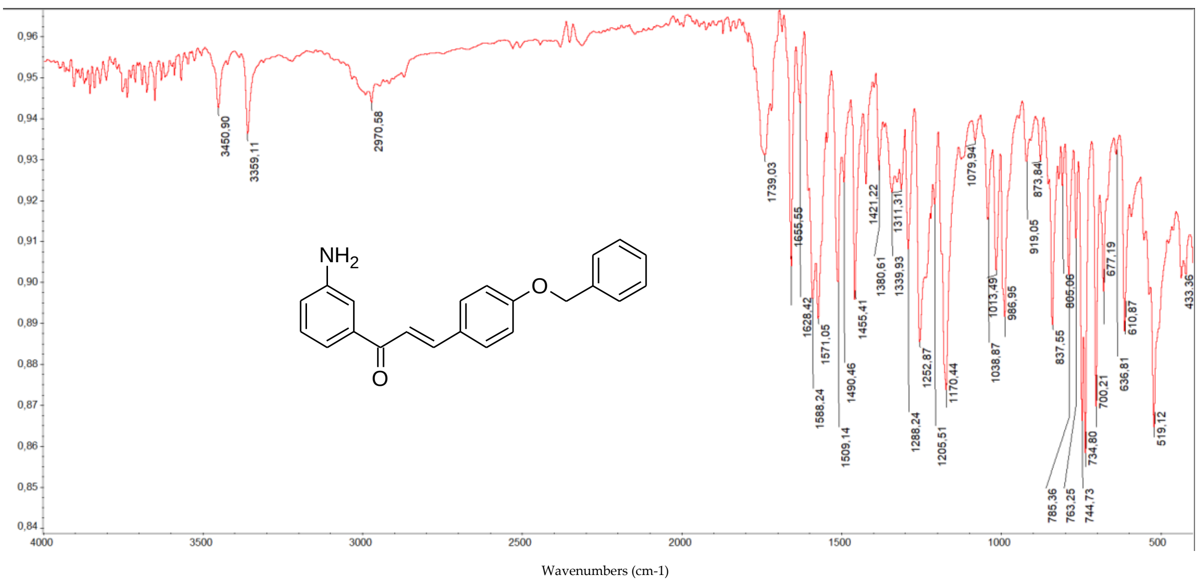

Figure S42. FTIR-ATR spectrum of 3′-amino-4-benzyloxychalcone (**14**)

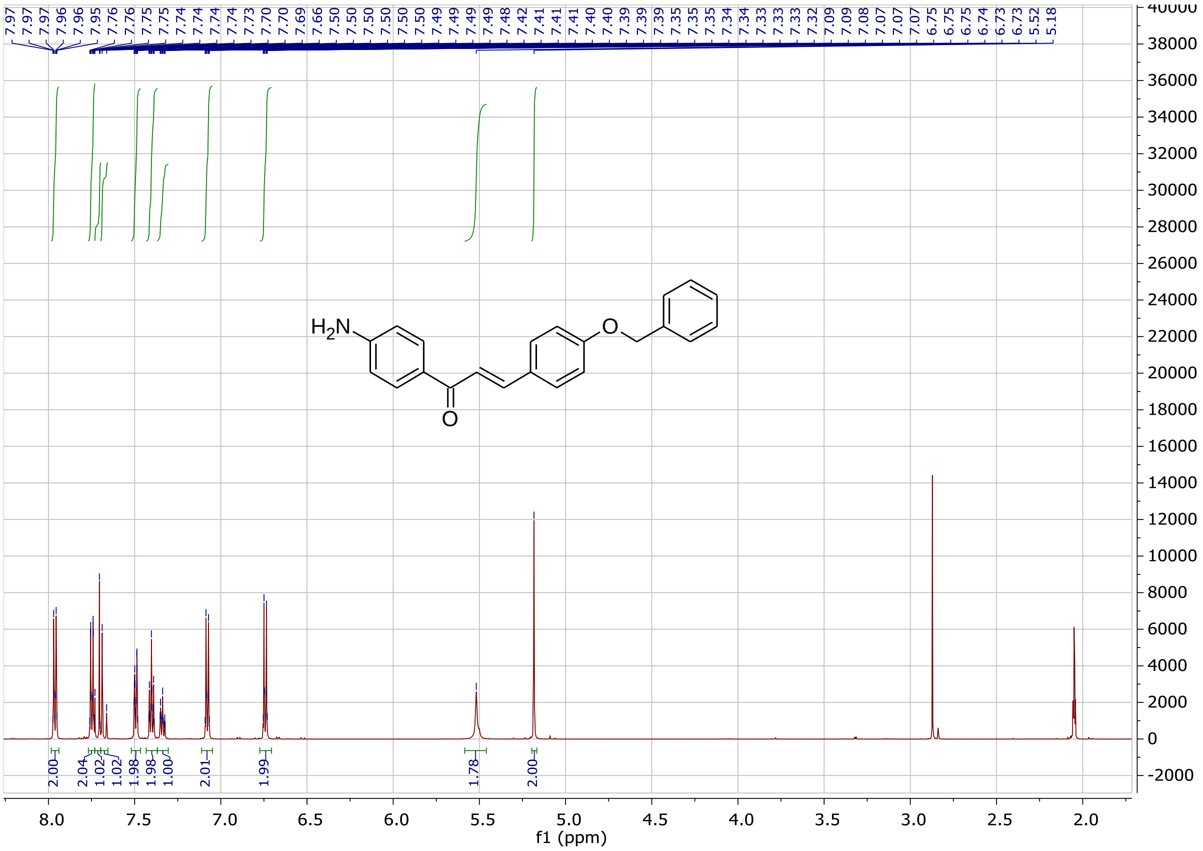

Figure S43. ^{1}H-NMR (600 MHz, acetone-d_6) spectrum of 4′-amino-4-benzyloxychalcone (**15**)

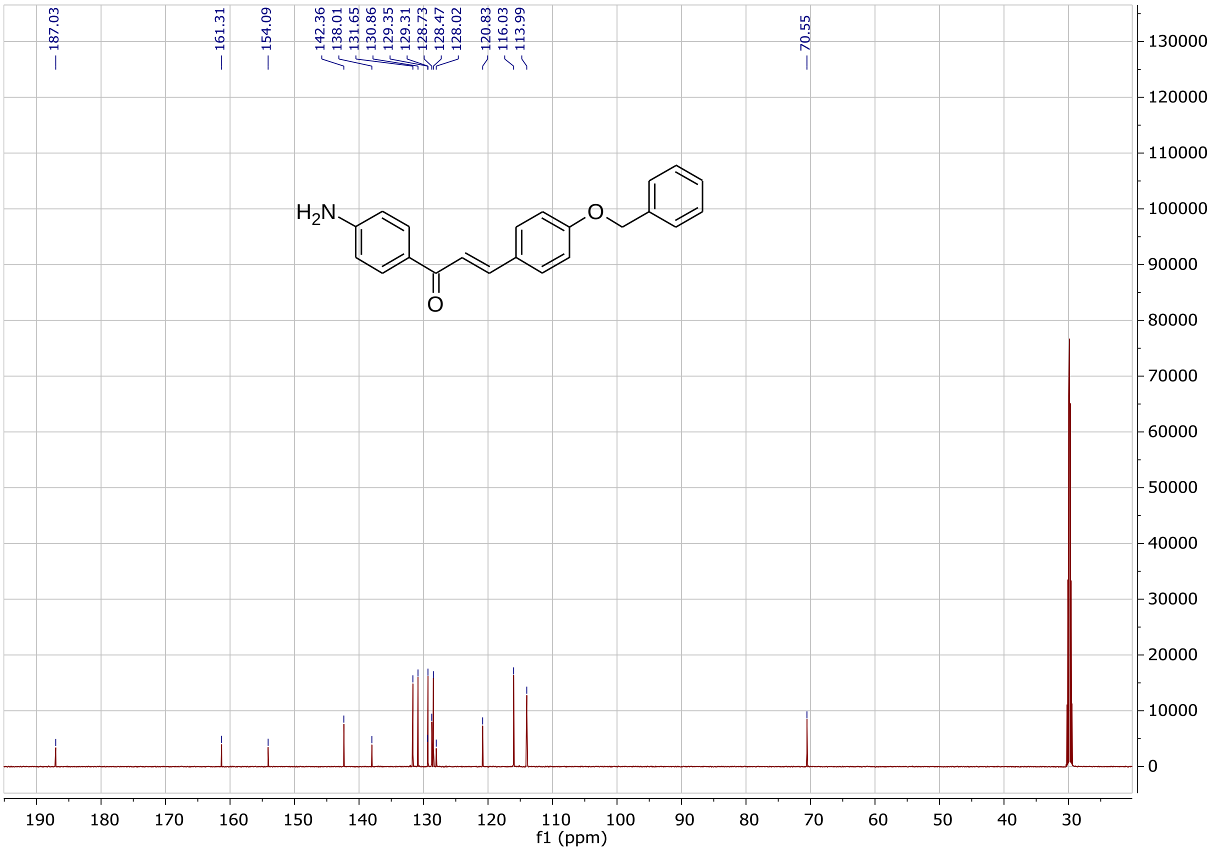

Figure S44. ^{13}C-NMR (150 MHz, acetone-d_6) spectrum of 4′-amino-4-benzyloxychalcone (**15**)

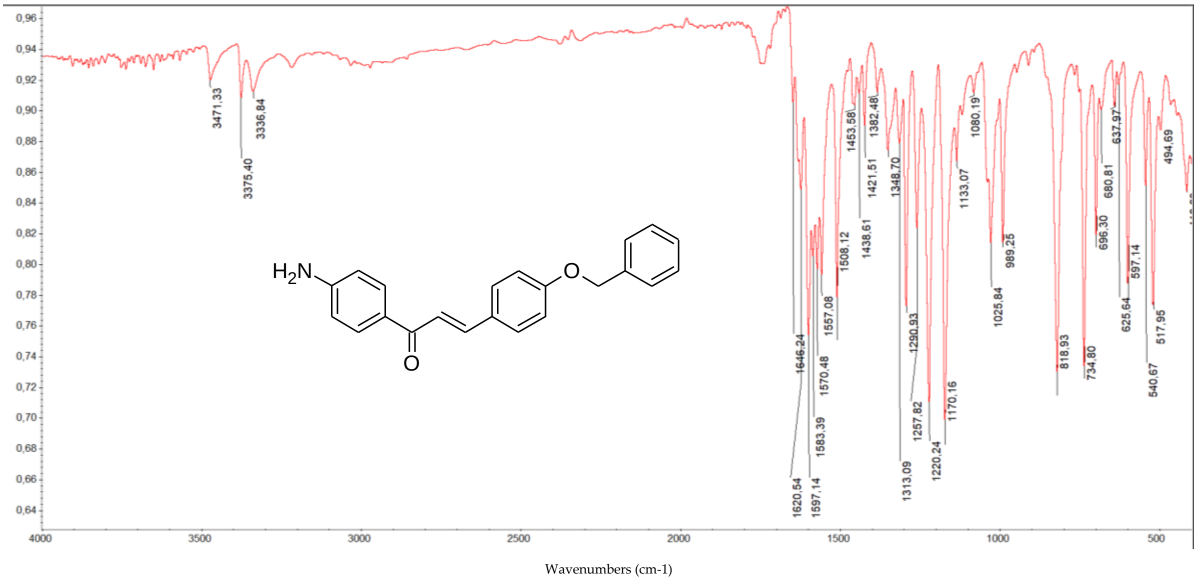

Figure S45. FTIR-ATR spectrum of 4′-amino-4-benzyloxychalcone (**15**)

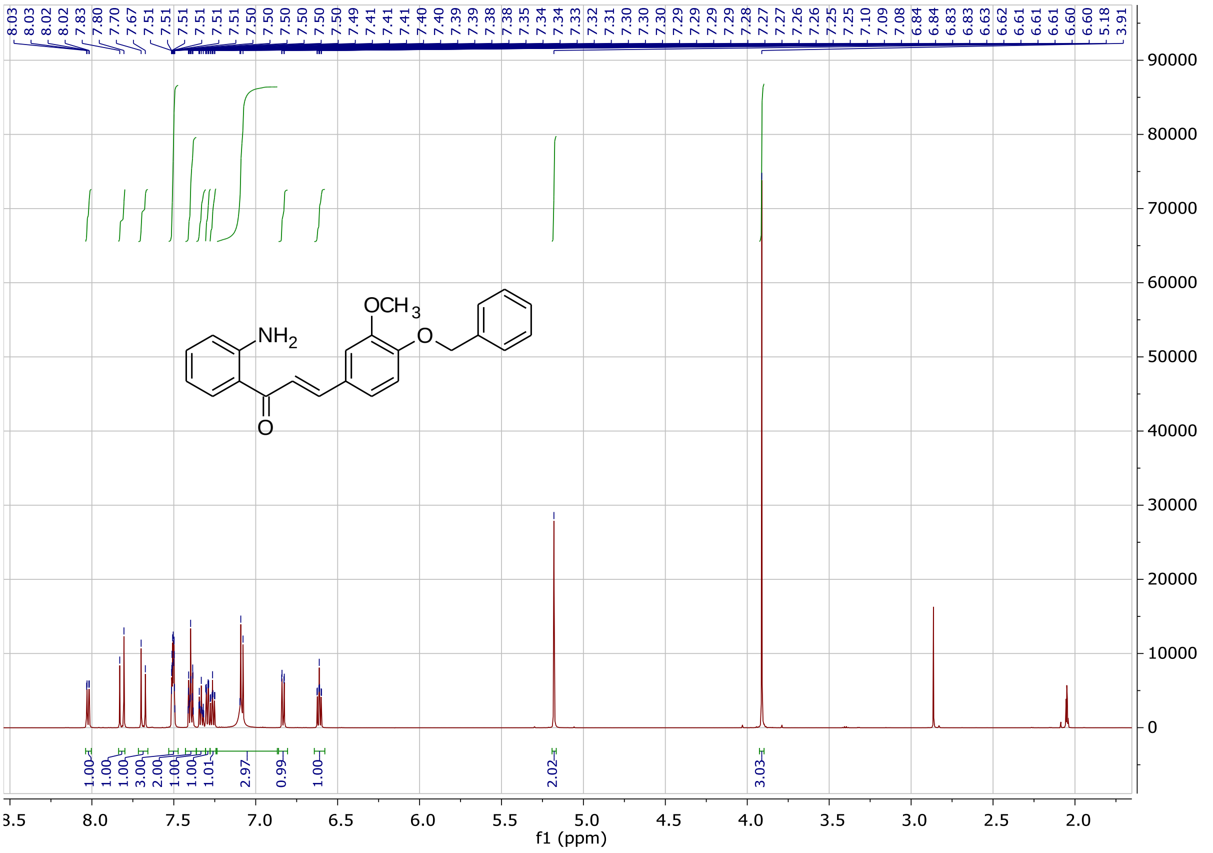

Figure S46. ^{1}H-NMR (600 MHz, acetone-d_6) spectrum of 2′-amino-4-benzyloxy-3-methoxychalcone (**16**)

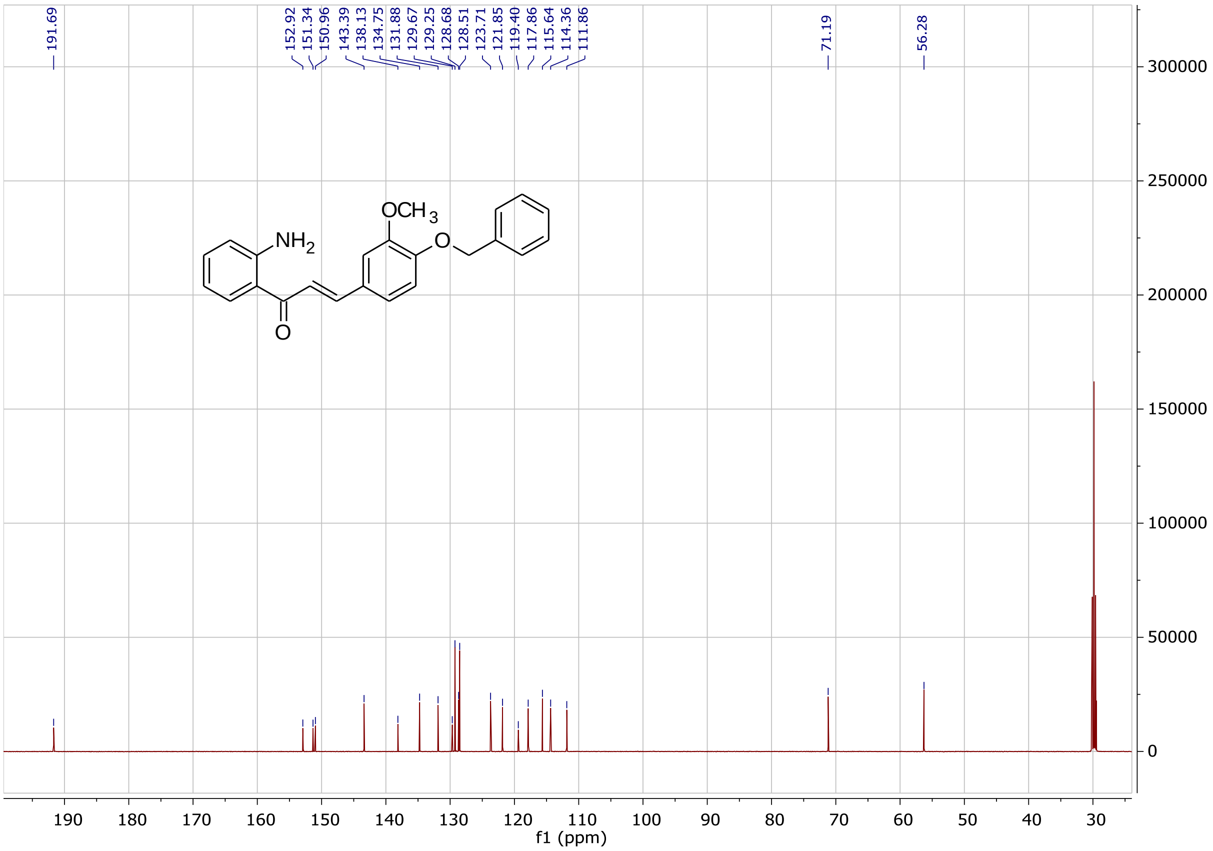

Figure S47. ^{13}C-NMR (150 MHz, acetone-d_6) spectrum of 2′-amino-4-benzyloxy-3-methoxychalcone (**16**)

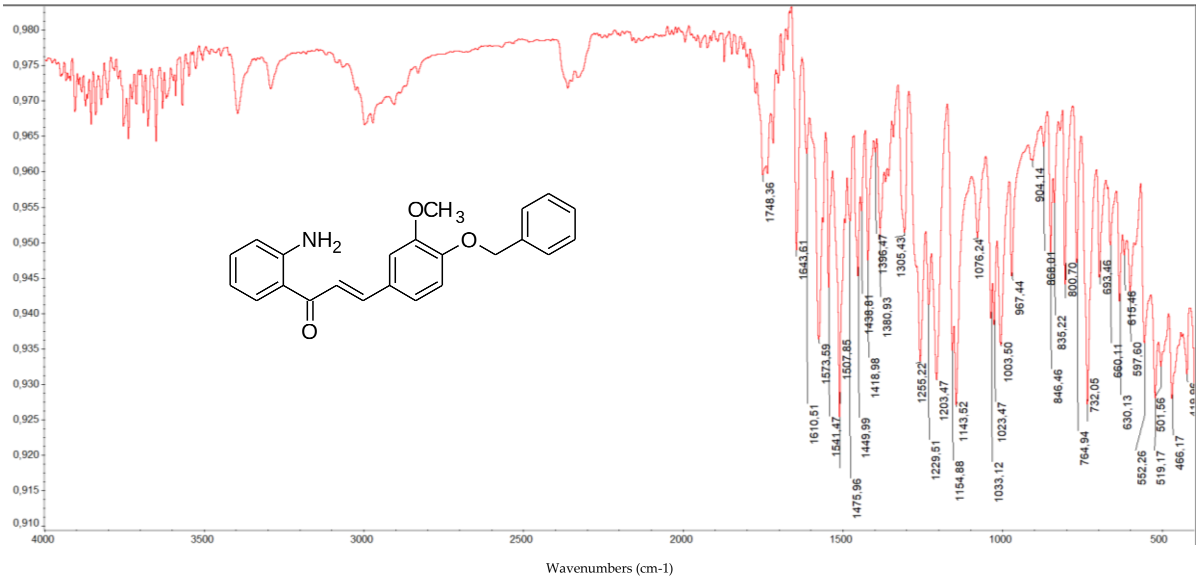

Figure S48. FTIR-ATR spectrum of 2′-amino-4-benzyloxy-3-methoxychalcone (**16**)

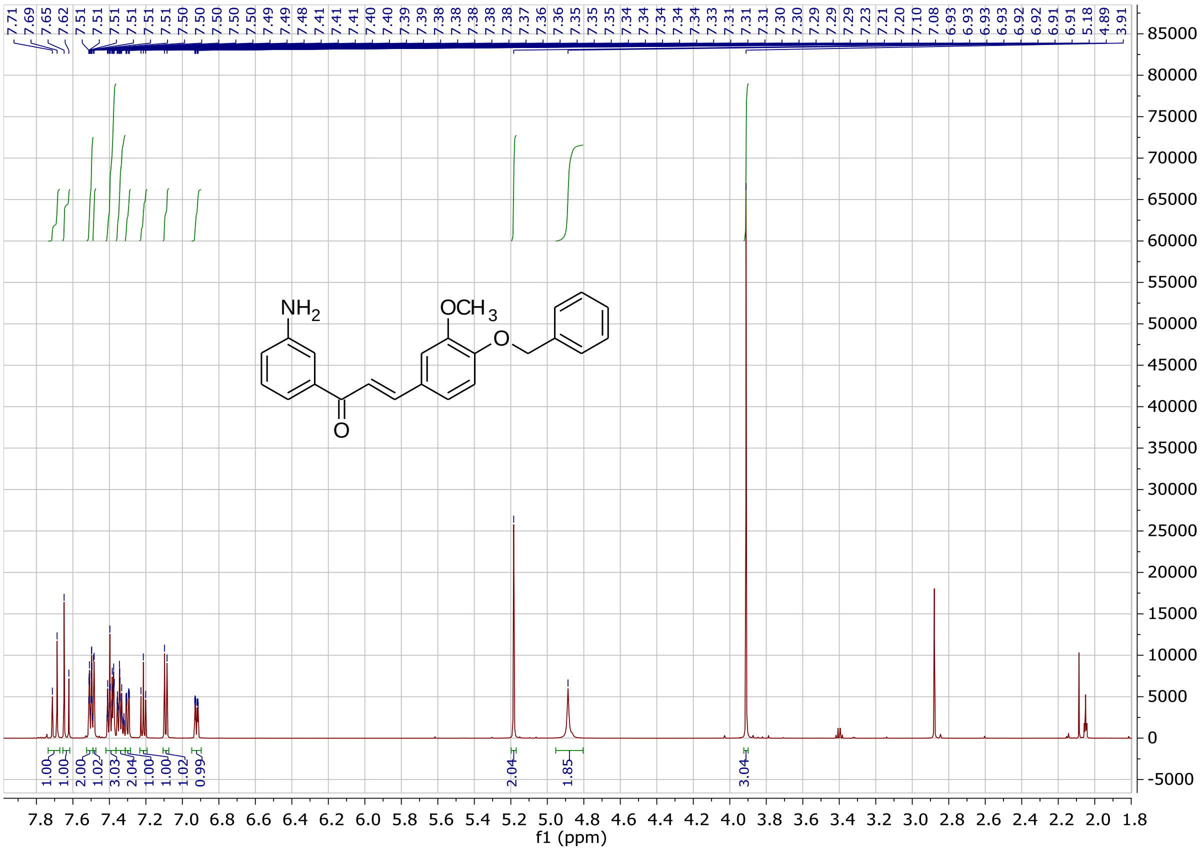

Figure S49. ^{1}H-NMR (600 MHz, acetone-*d*6) spectrum of 3′-amino-4-benzyloxy-3-methoxychalcone (**17**)

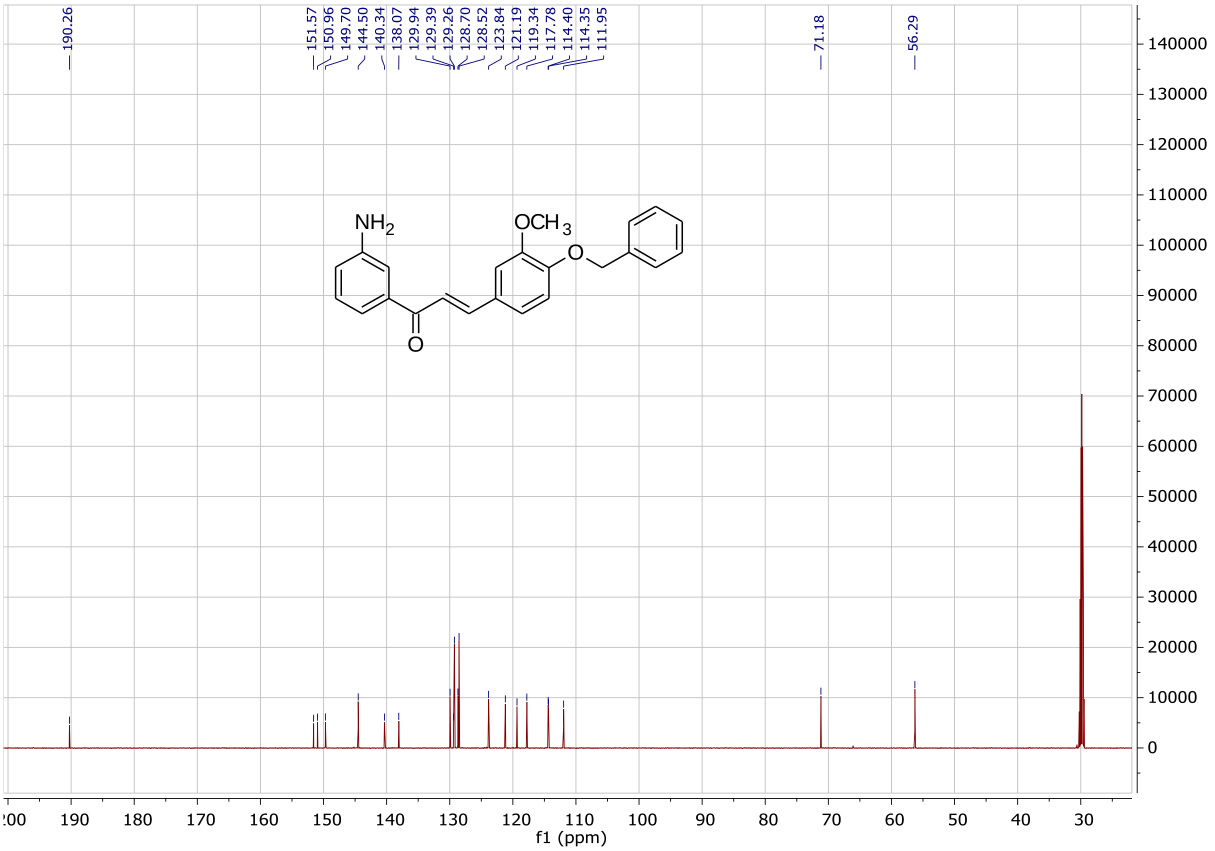

Figure S50. ^{13}C-NMR (150 MHz, acetone-d_6) spectrum of 3′-amino-4-benzyloxy-3-methoxychalcone (**17**)

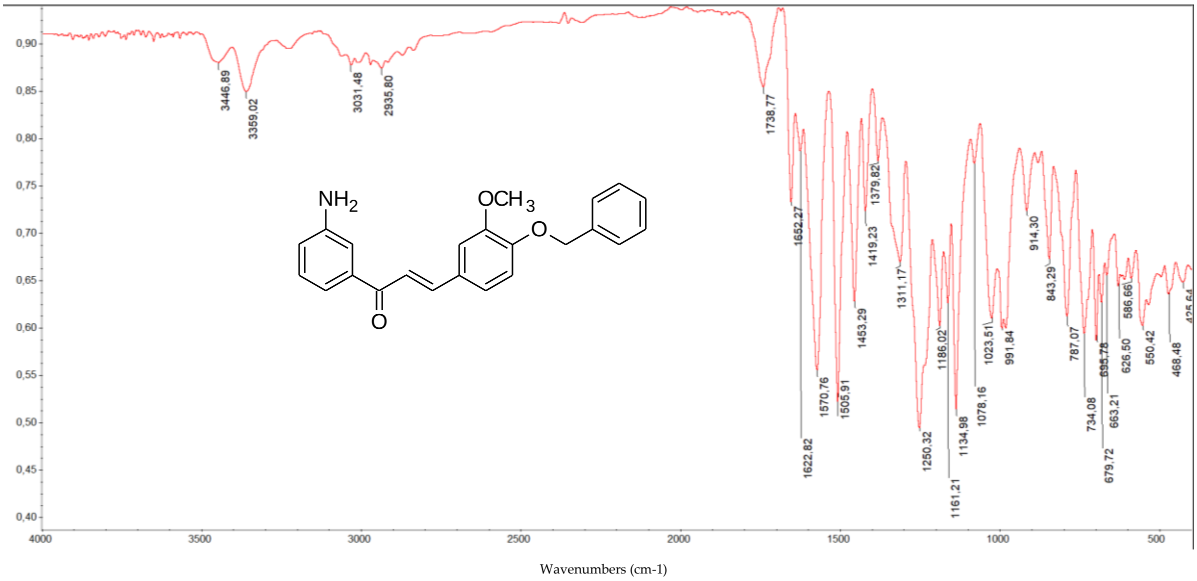

Figure S51. FTIR-ATR spectrum of 3′-amino-4-benzyloxy-3-methoxychalcone (**17**)

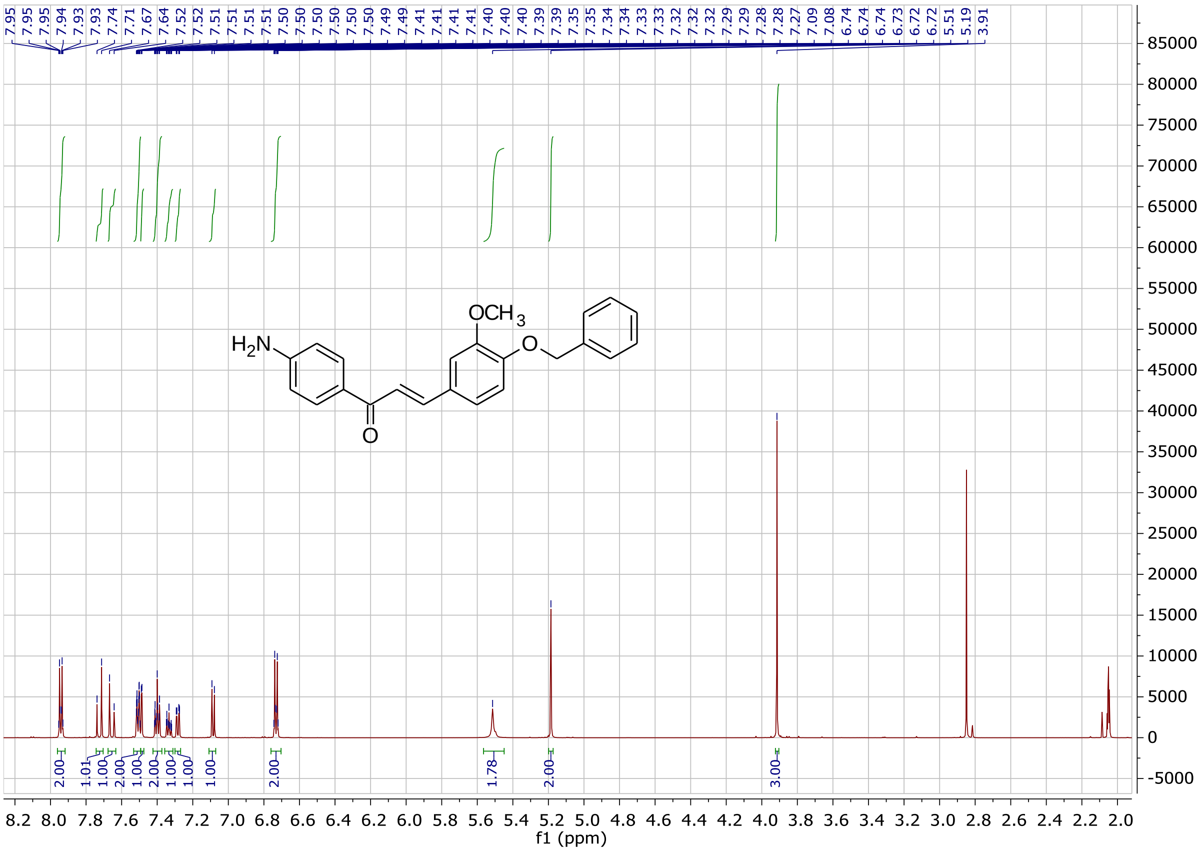

Figure S52. ^{1}H-NMR (600 MHz, acetone-d_6) spectrum of 4′-amino-4-benzyloxy-3-methoxychalcone (**18**)

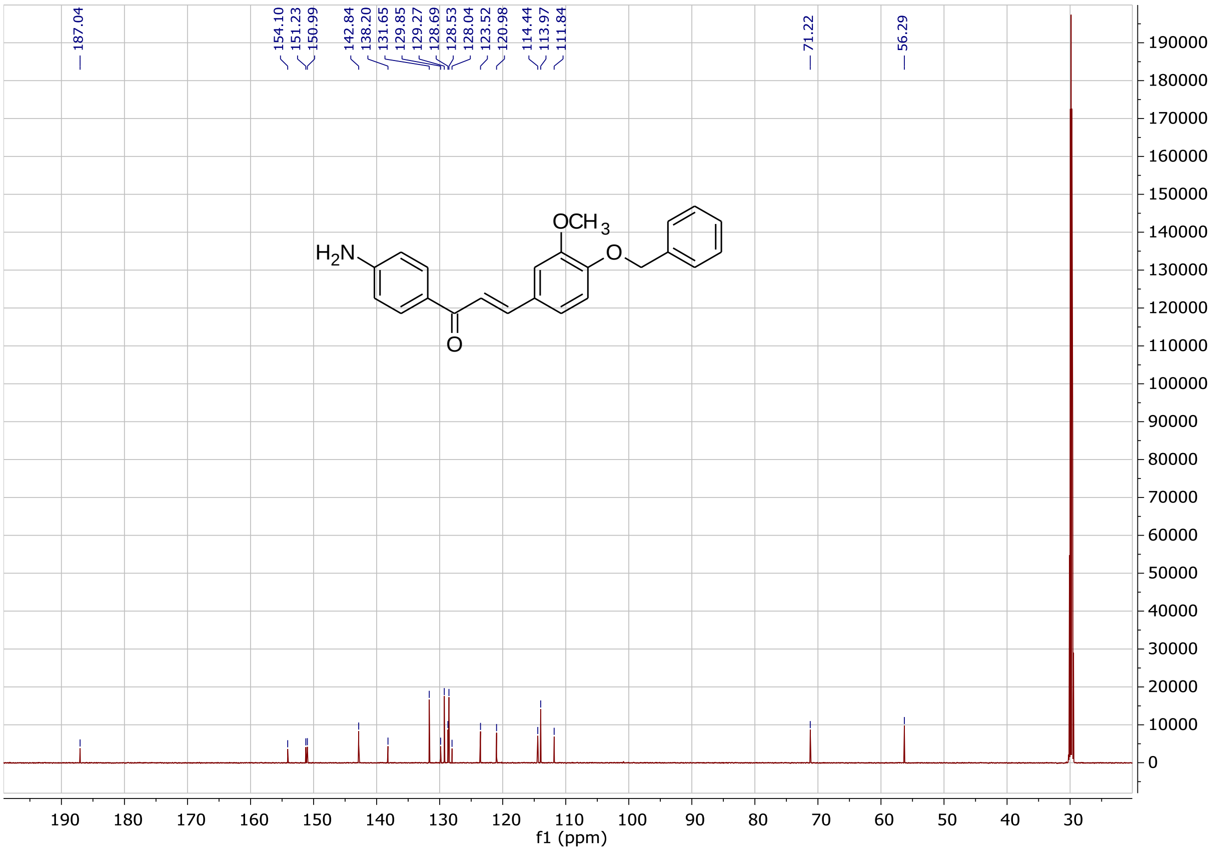

Figure S53. ^{13}C-NMR (150 MHz, acetone-d_6) spectrum of 4′-amino-4-benzyloxy-3-methoxychalcone (**18**)

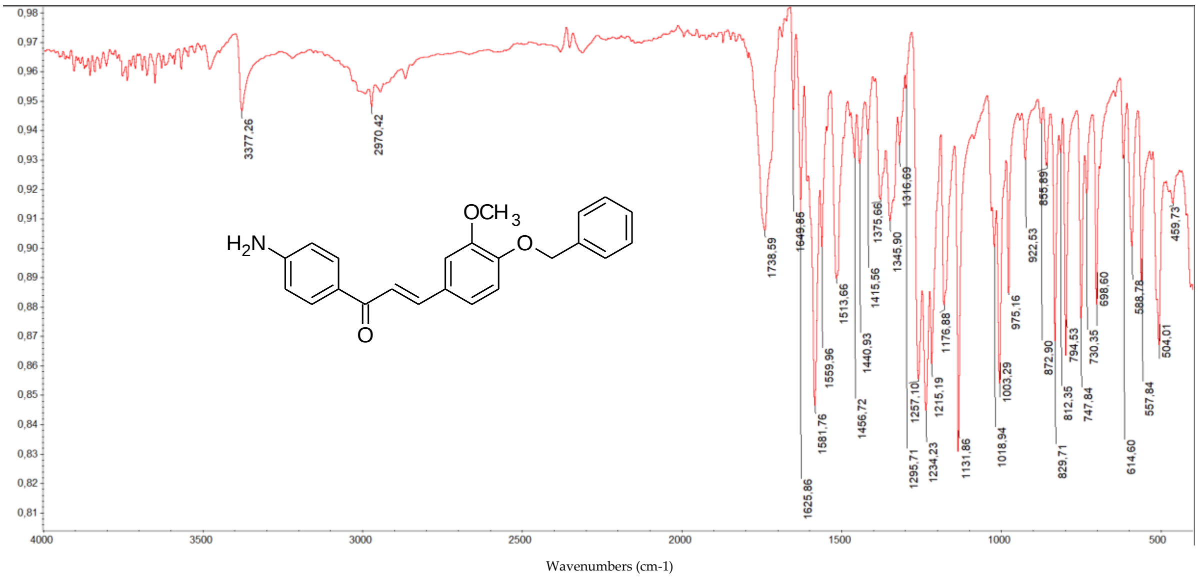

Figure S54. FTIR-ATR spectrum of 4′-amino-4-benzyloxy-3-methoxychalcone (**18**)
